# Chiral Bromonium Salt (Hypervalent Bromine(III)) with *N*-Nitrosamine as a Halogen-Bonding Bifunctional Catalyst

**DOI:** 10.3390/molecules28010384

**Published:** 2023-01-02

**Authors:** Yasushi Yoshida, Tatsuya Ao, Takashi Mino, Masami Sakamoto

**Affiliations:** Molecular Chirality Research Center, Graduate School of Engineering, Chiba University, 1-33, Yayoi-cho, Inage-ku, Chiba 263-8522, Japan

**Keywords:** halogen bonding, bromonium salt, hypervalent bromine, *N*-nitrosamine, asymmetric catalysis

## Abstract

There has been a great focus on halogen-bonding as a unique interaction between electron-deficient halogen atoms with Lewis basic moieties. Although the application of halogen-bonded atoms in organic chemistry has been eagerly researched in these decades, the development of chiral molecules with halogen-bonding functionalities and their utilization in asymmetric catalysis are still in the\ir infancy. We have previously developed chiral halonium salts with amide functionalities, which behaved as excellent catalysts albeit in only two reactions due to the lack of substrate activation abilities. In this manuscript, we have developed chiral halonium salts with an *N*-nitrosamine moiety and applied them to the Mannich reaction of isatin-derived ketimines with malonic esters. The study focused on our novel bromonium salt catalyst which provided the corresponding products in high yields with up to 80% ee. DFT calculations of the chiral catalyst structure suggested that the high asymmetric induction abilities of this catalyst are due to the Lewis basic role of the *N*-nitrosamine part. To the best of our knowledge, this is the first catalytic application of *N*-nitrosamines.

## 1. Introduction

Halogen-bonding (XB), which is the non-covalent interaction formed between electron-deficient halogen atoms and Lewis basic moieties, has been focused on in the wide field of chemistry due to its unique power [1,2,3,4]. The key feature of XB is its relatively strong yet soft character, providing higher directionality compared to hydrogen-bonding. In organic chemistry, XB has been employed for molecule recognition and for the activation of electron-rich substrates in several reactions [5,6]. In the past decade, XB has been utilized as a key interaction in metal catalysis and organocatalysis, demonstrating its characteristic reactivities and selectivities in various reactions [7,8,9,10,11,12,13,14,15,16,17]. In 2022, Fiksdahl, Erdelyi and co-workers reported the cyclopropanation of propargyl acetate with styrene derivatives under gold catalysis [8]. In their reaction, the addition of a halogen bond donor as a co-catalyst drastically increased the reaction rate.

**Figure 1:**
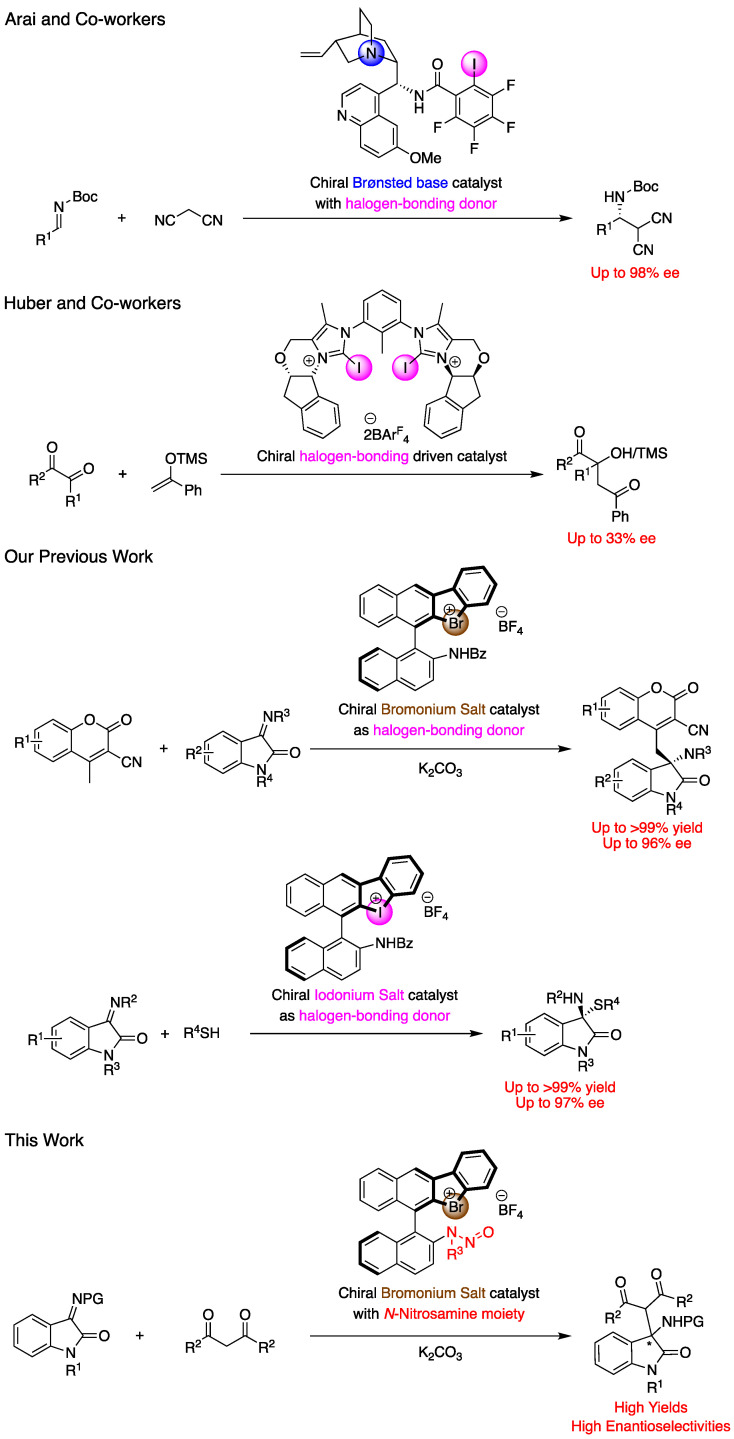
Examples of chiral halogen-bonding catalysts.

In asymmetric catalysis, the application of XB has been limited [18,19,20,21,22,23,24]. In 2014, Tan and co-workers reported the alkylation reaction of sulfenes with alkyl halides catalyzed by an ammonium salt with halogen atom, to form the corresponding products with up to 96% ee [18]. In their report, the iodine atom played a crucial role and switching to other halogen atoms provided decreased enantioselectivities. In 2018, Arai and co-workers developed the *cinchona*-derived Brønsted base catalyst with the halogen-bonding functionality of the perfluoro iodophenyl group, which effectively catalyzed the Mannich reaction of malononitrile with isatin-derived ketimines to afford products in high yields with up to 97% ee (Figure 1) [20]. Finally, in 2021, Huber and co-workers developed the bulky bis(2-iodoimidazolium) based chiral molecules, which catalyzed the Mukaiyama–aldol reaction of silyl ketene acetals with ketones, to provide the chiral products with up to 33% ee (Figure 1) [23].

Hypervalent halogen reagents, especially hypervalent iodine reagent, have been researched as highly reactive chemical species which act as substrates and as catalysts in oxidation reactions [25,26,27,28,29,30,31]. Although the iodonium and bromonium salts, which are kinds of hypervalent halogen compounds, have been found to work as XB catalysts [32,33,34,35,36,37], findings on their chiral equivalents to be used for asymmetric catalysis have been quite limited thus far [38,39,40]. In 2015, Liu, Han, and co-workers reported the syntheses of iodonium salts with a chiral phosphate counter anion, and their application as asymmetric XB catalysts in a three-component Mannich reaction to form the desired products with up to 7% ee [38]. Recently, we have developed chiral binaphthyl-based cyclic iodonium and bromonium salts with amide functionalities, which efficiently catalyzed the vinylogous Mannich reaction of cyanomethyl coumarins with imines, as well as the thiol addition reaction to isatin-derived ketimines in high yields with up to 96% and 97% ee, respectively (Figure 1) [39,40]. Although our chiral halonium salts worked as excellent catalysts, they could not be applied to other kinds of reactions due to the difficulties in introducing other functionalities except the amide group. Compounds with the *N*-nitrosamine moiety have been utilized as precursors of azomethine imines [41], as directing groups in C–H functionalization by transition metal catalysis [42,43,44], and as active substrates in several reactions [45]. Therefore, this study aimed to develop chiral bromonium salts with *N*-nitrosamine functionalities, which behaved as a stronger Lewis basic part than previous amide group, and explore their applications in the enantioselective Mannich reaction of malonic esters with ketimines. To the best of our knowledge, this is the first example of the successful enantioselective catalytic applications of *N*-nitrosamines.

## 2. Results and Discussion

### 2.1. Synthesis of Catalysts

**Scheme 1:**
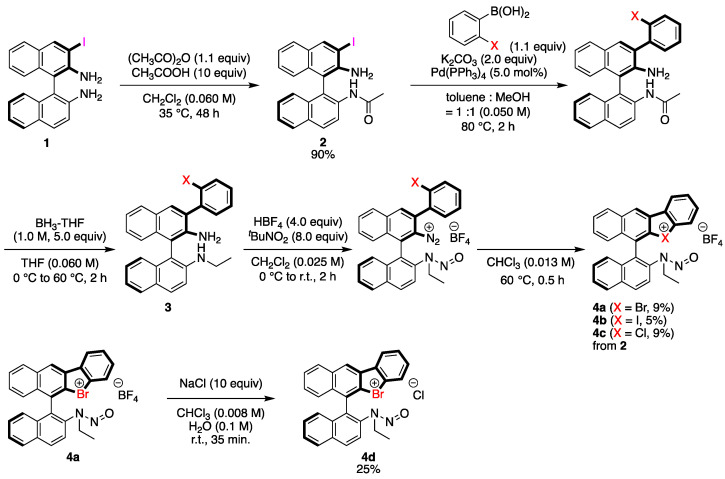
Synthesis of chiral halonium salts with *N*-nitrosamine moiety.

The chiral binathyl-based halonium salts were synthesized as follows (Scheme 1). Commercial (*R*)-BINAM was chosen as a starting material, which was converted to 3-iodo-BINAM **1** according to the reported procedure [46]. The acylation of **1** was conducted with 1.1 equivalents of an acetic anhydride under acidic condition to obtain amide **2** in 90% yield with almost perfect chemoselectivity. The 2-halophenyl group was introduced to **2** through the Suzuki coupling reaction with 1.1 equivalents of 2-halophenylboronic acid under palladium catalysis to form the intermediate, and its amide parts were reduced into amine functionalities by the treatment with 5.0 equivalents of borane in THF to give **3**. Finally, the primary and the secondary amino groups of **3** were converted to the diazonium salt and the *N*-nitrosamine moiety, respectively, by the treatments with 8.0 equivalents of *tert*-butyl nitrite and 4.0 equivalents of tetrafluoroboric acid, which were then cyclized through the pyrolysis of diazonium moieties in chloroform at 60 °C to form the desired halonium salts **4** in low to moderate yields; **4d** was synthesized through the counteranion exchange of **4a** by treatment with 10 equivalents of NaCl in 25% yield.

### 2.2. Application of Chiral Catalysts

**Scheme 2:**
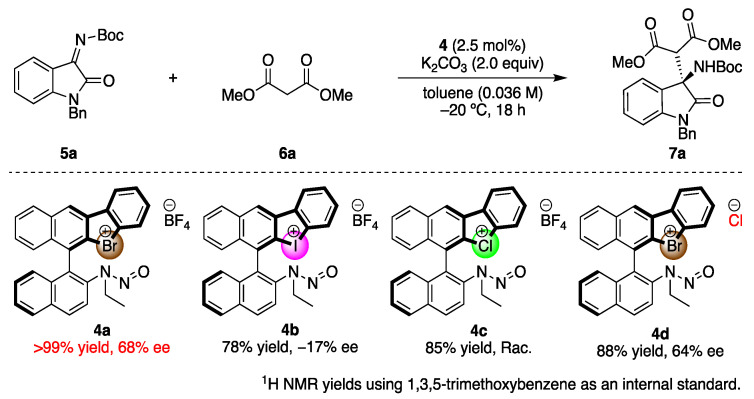
Screening of the chiral halonium salt catalysts.

The synthesized chiral halonium salts with *N*-nitrosamine moieties were applied to enantioselective reactions (Scheme 2). We chose the Mannich reaction of malonic esters to ketimines as a model reaction, as it affords valuable chiral amines in an enantioselective fashion [47,48]. The catalyst screenings were conducted in this reaction. First, the employed halogen atoms were screened, and the bromonium salt **4a** was observed to have provided the corresponding products in the highest yield with enantioselectivity. This result revealed that the *N*-nitrosamine moiety could be included to the chiral catalyst structure as a Lewis basic part, which demonstrated the high asymmetric induction abilities. The iodonium salt **4b** also worked as an asymmetric catalyst in the present reaction, but decreased enantioselectivity, and the chloronium salt **4c** catalyzed the reaction only in racemic form. These reasons can be explained by the strength of the halogen bond of the halogen atoms. The bromonium salt interacted with the substrate in its bidentate form, composed of both halonium salt and *N*-nitrosamine moiety, to construct an efficient asymmetric environment around the prochiral carbon center, and form the products in good enantioselectivities. Conversely, the iodonium salt bears a strong halogen bond donor character, which enabled the intramolecular interaction with *N*-nitrosamine part to form the monodentate catalyst and it mediated the reaction in low and reversed enantioselectivity. The chloronium salt weakly coordinated to the imine substrate, and the background reaction went faster to form the product in racemic form. To investigate the importance of the halogen bond in the present catalysis, the bromonium salt with chloride anion **4d** was synthesized and applied, which afforded the corresponding product in decreased enantioselectivity. The chloride anion is known to make a halogen bond with halogen atoms such as bromonium moieties, which weaken the halogen bond of the catalyst with a substrate. This result suggests the importance of the halogen bond for the enantioinduction to the products in the present reaction.

After the optimal catalyst was established, the reaction conditions were optimized (Table 1). Reaction in non-polar solvents such as toluene, THF, and dichloromethane provided the products in excellent yields with high enantioselectivities (Entries 1–3); however, the employment of polar solvents (diethyl ether and acetonitrile) gave diminished enantioselectivities (Entries 4 and 5). These results suggest the importance of the Lewis basic interaction of the *N*-nitrosamine moiety in the product’s enantioselectivity, which was disturbed by the hydrogen bond of the polar solvents. Subsequently, the catalyst loading, and the reaction temperatures were optimized, and the reaction in toluene at −20 °C with 2.5 mol% of **4a** was found to be optimal. The lower catalyst loading caused the shortage of the chiral catalyst due to its decomposition under basic condition, which relatively accelerated the background reaction, and the racemic product was obtained. On the other hands, the higher catalyst concentration perhaps increased the intermolecular interaction of **4a**, which formed the oligomeric catalyst species, and it worked as an inferior asymmetric catalyst, and the enantioselectivity of the product decreased [40].

**Figure 2:**
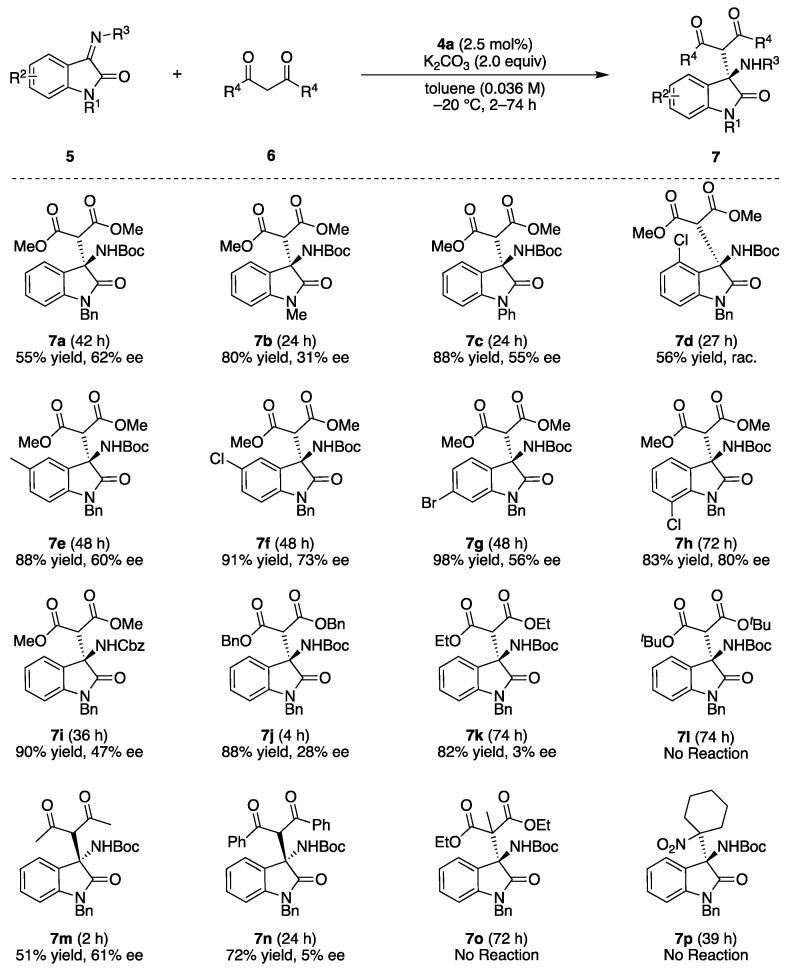
Substrate scopes. Isolated product yields are shown.

After obtaining the optimal conditions, the substrate scope for the study transformation was conducted (Figure 2). Most of the reactions were conducted with excess longer time because the products had very similar RF values to substrates on TLC, which made it difficult to judge the completion of reactions. The scopes for imines and *N*-protecting group of benzyl and phenyl moieties tolerated the reaction well, and products **7a** and **7c** were isolated in high yields with high ee, while the methyl protected product **7b** was obtained with a lower ee of 31%. The aromatic substituents of the imines were investigated, and the corresponding products **7d**–**7h** were obtained in high yields with high ee’s, irrespective of their steric and electronic natures except for 4-chlorinated **7d**, which was obtained in a racemic form. *N*-Benzyloxycarbonyl- (Cbz) protected imine **5i** could also be applied well to the present reaction to form **7i** in good enantioselectivity. Next, the scopes for nucleophiles were conducted. Scopes for malonic ester derivatives revealed that the enantioselectivities of the products were highly affected by the bulkiness of the nucleophiles, and a sterically less-hindered dimethyl malonate provided product **7a** with a high ee. However, the sterically hindered dibenzyl malonate and diethyl malonate gave **7j** and **7k** with only 28% ee and 3% ee, respectively, and the more hindered di-*tert*-butyl malonate did not provide any products. Other active methylene compounds were also employed as pre-nucleophiles. The reaction outcomes had almost same trend to that of the malonic esters, as the sterically less-hindered acetylacetone provided **7m** in 51% yield with 61% ee, and the hindered dibenzoyl methane and tertiary nucleophile gave poor results (**7n** and **7o**, respectively). Nitrocyclohexane was also applied as a pre-nucleophile, but no reaction was observed. The bulky nucleophiles were interacted with chiral catalyst weaker than that of less-bulky one, which decreased the *Si*-face selectivity in the enantioselectivity determining step. The absolute configurations of the **7b** and **7m** were determined according to the HPLC retention times of the reported values, and the other configurations were determined according to their structures [47,48].

**Figure 3:**
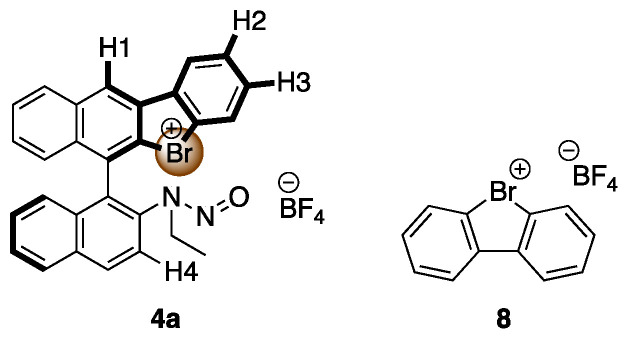
Structure of **4a** and **8**.

**Figure 4:**
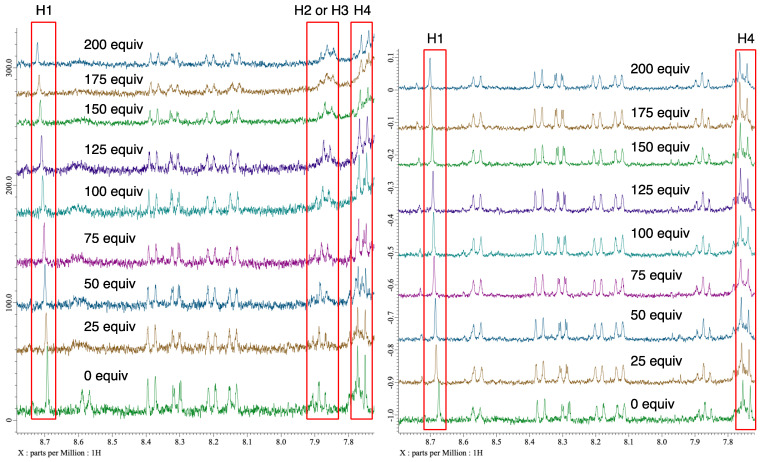
^1^H NMR charts (in CDCl_3_) of **4a** with different equivalents of **5a** (**left**), and **6a** (**right**).

**Figure 5:**
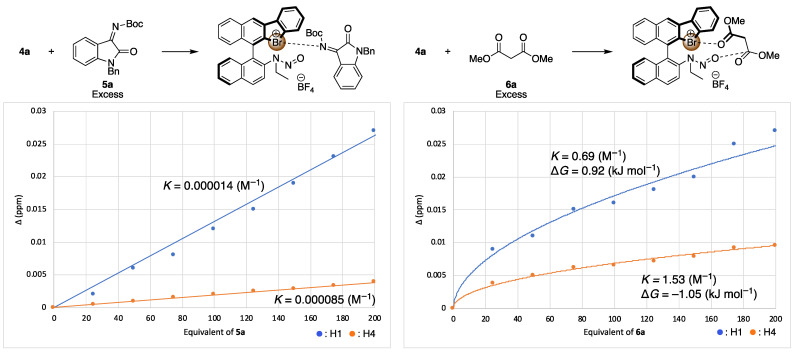
^1^H NMR titration experiments (in CDCl_3_) of **4a** with **5a** (**left**), and **6a** (**right**). The spots show the chemical shift differences from additive-free values at various equivalent of additives. Blue spot means the chemical shifts differences for H1, and the orange spot means that of H4 (Figure 4). The binding constants *K* were calculated by Bindfit.

Next, to verify the asymmetric induction mechanism of the present reaction, the interactions between the chiral halonium salt with an imine **5a** or a dimethyl malonate (**6a**) were investigated by ^1^H NMR titration experiments (Figure 3 and Figure 4) [49]. Due to the instability of **4a** under a basic condition, these titration experiments were conducted in the absence of potassium carbonate. When **4a** was mixed with different equivalents of imine **5a**, the ^1^H NMR chemical shifts of H1, H2 or H3 and H4 were significantly shifted as increasing the amount of **5a** (Figure 4, left). On the other hand, when **4a** was mixed with dimethyl malonate, the ^1^H NMR chemical shifts of only H1 and H4 were shifted (Figure 4, right). These observations suggested that catalyst **4a** had interaction with both an imine substrate and a dimethyl malonate. To get the insight into a more detailed activation pattern, binding constants (*K*) and Gibbs free energies (Δ*G*) were determined by Bindfit program (Figure 5) [50,51]. Because the protons for H2 or H3 were rather overlapped, the binding constants were calculated only using H1 and H4 protons. The spots in Figure 5 shows the absolute values for chemical shift differences of **4a** by mixing with **5a** (left) or **6a** (right). In both cases, the chemical shifts were highly affected by the quantity of additives, although, their binding constants were significantly different from each other. When the binding constants for the cases of mixing **4a** with **5a** were calculated, the *K* values were almost 0 for both H1 and H4. On the other hands, the *K* values for mixing **4a** with **6a** were 0.69 and 1.53 M^−1^ for H1 and H4, respectively. These results suggested that **4a** did not interact with **5a** but only **6a**. Furthermore, the titration curve indicated that both H1 and H4 of **4a** were affected by the binding with **6a**, which located close to the bromonium salt moiety and the *N*-nitrosamine part, respectively. These observations suggests that **4a** binds and activates dimethyl malonate with both a bromonium and a *N*-nitrosamine part even under the neutral condition.

**Figure 6:**
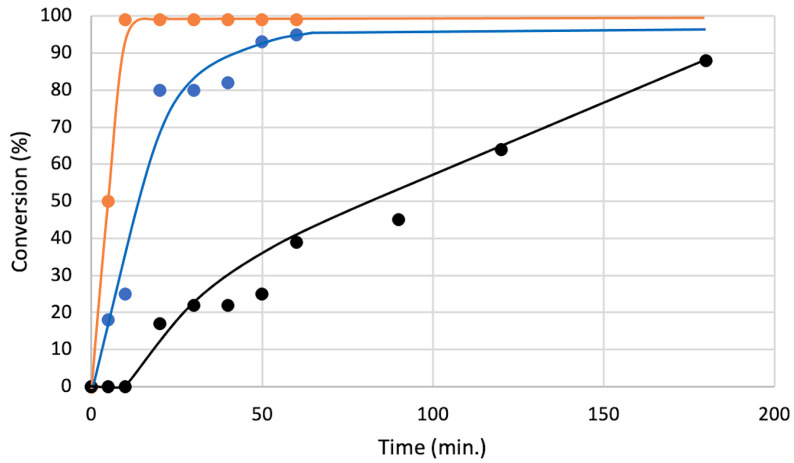
Conversions of **5a** in an optimal condition (Table 1, Entry 1) with different catalyst (black: without catalyst, blue: with **4a** (2.5 mol%), orange: with **8** (2.5 mol%)) monitored by ^1^H NMR.

Finally, to check the reaction acceleration ability of our chiral catalyst, the reaction conversions were monitored by ^1^H NMR (Figure 6). When the reaction of **5a** with **6a** was conducted under the optimized condition without catalyst, the reaction was very slow and considerable amount of **5a** was remained even after 180 min (black line). On the other hands, when the same reaction was carried out in the presence of 2.5 mol% of **4a**, the reaction was drastically accelerated and most of **5a** was consumed after 60 min (blue line). Interestingly, the reaction was more efficiently catalyzed by the achiral bromonium salt **8**, which gave the almost complete conversion of **5a** only within 10 min (orange line). These results suggest that **4a** has lower catalytic activity than **8** due to its bulkiness and the half number of sigma holes, which can activate a substrate.

**Figure 7:**
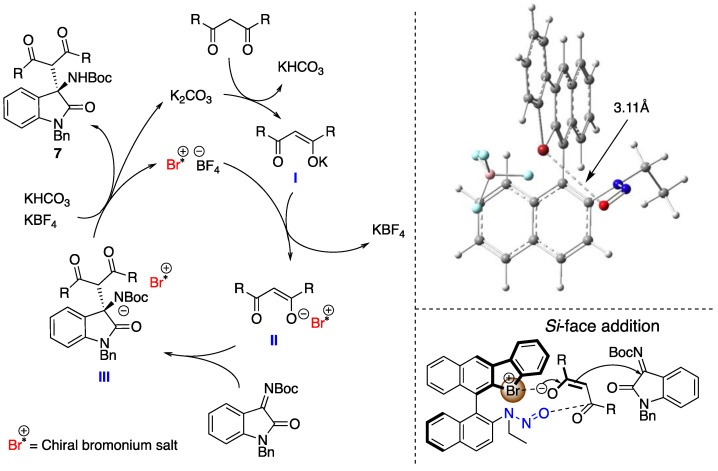
Plausible reaction mechanism (**left**), the DFT-calculated optimized structure of **4a** (M06-2X/6-31G(d)) generated by *GaussView 6* (**right**, upper), and the plausible transition state structure (**right**, lower). The Br···O distance of the calculated structure is 3.11 Å.

From mechanistic studies, the plausible reaction mechanism for the present transformation was proposed (Figure 7). First, the proton of the active methylene moiety was deprotonated by potassium carbonate to form an ionic intermediate. Then, the counteranion of the latter was exchanged by the chiral halonium salt catalyst to form chiral intermediate **II**, which worked as a nucleophile with imines to form **III**. Finally, protonation of **III** provided desired product **7** together with the regeneration of the catalyst. In this catalytic cycle, the step from intermediate **II** to **III** is the enantioselectivity determining step. In this step, the plausible transition structure is constructed according to the ^1^H NMR titration experiment results of mixing the chiral catalyst with an imine or a methyl malonate. The halonium salt activated an enolate anion of the active methylene species-derived molecule by halogen-bonding, and the *N*-nitrosamine part recognized another carbonyl carbon atom in the same molecule. The structure of **4a** was optimized at the M06-2X/6-31G(d) level of theory in gas phase within Gaussian 16 [52], with structures generated by *GaussView 6* [53]. The Br···O distance of the optimized structure was 3.11 Å, which suggested the independently existence of both the bromonium salt and the *N*-nitrosamine part [1].

## 3. Materials and Methods

### 3.1. General Information

^1^H-, ^13^C-NMR spectra were recorded with Bruker AVANCE III-400M (^1^H-NMR 400 MHz, ^13^C-NMR 100 MHz, ^19^F-NMR 376 MHz). ^1^H-NMR spectra are reported as follows: chemical shift in ppm (δ) relative to the chemical shift of CHCl_3_ at 7.26 ppm or tetramethylsilane at 0 ppm, integration, multiplicities (s = singlet, d = doublet, t = triplet, q = quartet, m = multiplet), and coupling constants (Hz). ^13^C-NMR spectra reported in ppm (δ) relative to the central line of triplet for CDCl_3_ at 77 ppm. CF_3_CO_2_H used as external standards for ^19^F. ESI-MS spectra were obtained with Thermo Fisher, Exactive. FT-IR spectra were recorded on a JASCO FT-IR system (FT/IR-460 Plus). Mp was measured with AS ONE ATM-02. Column chromatography on SiO_2_ and neutral SiO_2_ was performed with Kanto Silica Gel 60 (40–50 μm). All reactions were carried out under Ar atmosphere unless otherwise noted. Commercially available organic and inorganic compounds were purchased from TCI, Kanto Chemical CO., INC. Wako Pure Chemical Industries, Ltd. or Nacalai Tesque, Inc., which had >95% purities, and used them without further purification. All dehydrated solvents were purchased from Wako Pure Chemical Industries, Ltd. or Nacalai Tesque, Inc., and were used without further purification. Calculation of binding constants were conducted by Bindfit program.

Imine substrates were synthesized according to the reported procedures [39,47,54,55,56,57]. Malonic esters and other active methylene species were purchased from the commercial source.

The raw data for the determination of binding constants (Table S1), ^1^H, ^13^C, and ^19^F NMR charts of the catalyst and their synthetic intermediate, and the products, HPLC charts of the products, DFT calculation results are available in the Supplementary Materials.

### 3.2. Synthesis of Chiral Bromonium Salts

#### 3.2.1. Synthesis of **2**

To a stirred solution of **1** (1.0 equiv) in CH_2_Cl_2_ (0.1 M) was added AcOH (10 equiv) and Ac_2_O (1.1 equiv) at 0 °C. The reaction was refluxed for 48 h before quenching with the addition of excess amount of NaOH aq. (2.0 M) at 0 °C. The reaction mixture was extracted with CH_2_Cl_2_, dried over Na_2_SO_4_ and filtered. After the removal of solvent by evaporation, the crude product was obtained, which was purified by column chromatography (Silica-gel, hexane/ethyl acetate) to give **2**.

##### (*R*)-*N*-(2’-amino-3’-iodo-[1,1’-binaphthalen]-2-yl)acetamide (**2**)

White solid, 317.3 mg, 0.70 mmol, 90% yield.

m.p. = 188–190 °C; ^1^H-NMR (400 MHz, CHLOROFORM-D) δ 8.58 (d, *J* = 9.0 Hz, 1H), 8.47 (s, 1H), 8.00 (d, *J* = 9.0 Hz, 1H), 7.90 (d, *J* = 8.1 Hz, 1H), 7.69–7.72 (m, 1H), 7.40–7.44 (m, 1H), 7.18–7.28 (m, 3H), 7.10 (d, *J* = 8.4 Hz, 1H), 6.93 (s, 1H), 6.86 (d, *J* = 8.2 Hz, 1H), 4.08 (s, 2H), 1.85 (s, 3H); ^13^C-NMR (101 MHz, CHLOROFORM-D) δ 168.8, 142.1, 140.0, 134.9, 133.3, 131.9, 131.3, 129.6, 129.2, 128.3, 127.8, 127.15, 127.06, 125.3, 125.1, 123.7, 123.3, 121.1, 120.6, 110.6, 87.7, 24.7; HRMS (ESI^+^ in MeCN) calcd for C_22_H_18_ON_2_I [M + H] 453.0458 found 453.0453; IR (KBr) ν 2962, 1653, 1595, 1265, 819 cm^−1^; [α]^20^_D_ = +43.9 (*c =* 0.2, CHCl_3_).

#### 3.2.2. Synthesis of **4a**–**c**

**2** (1.0 equiv), 2-halophenyl boronic acid (1.1 equiv), K_2_CO_3_ (2.0 equiv), and Pd(PPh_3_)_4_ (5 mol%) were dissolved in degassed toluene/MeOH = 1/1 (0.1 M), which was refluxed for 2 h. The reaction was quenched by the addition of water, extracted with CH_2_Cl_2_, dried over Na_2_SO_4_ and filtered. After the removal of solvent by evaporation, the crude product was obtained, which was purified by column chromatography (Silica-gel, hexane/ethyl acetate) to give coupling product, which was used without further purification.

To a stirred solution of above obtained coupling product in THF (0.067 M) was added BH_3_ (1.0 M in THF, 5.0 equiv) at 0 °C, which was stirred at 60 °C for 2 h. The reaction was quenched by the addition of NaOH aq. (2.0 M) at 0 °C. The reaction mixture was evaporated and extracted with CH_2_Cl_2_, dried over Na_2_SO_4_ and filtered. After the removal of solvent by evaporation, the crude product was obtained, which was purified by column chromatography (Silica-gel, hexane/ethyl acetate) to give **3**. **3** was obtained as a diastereomeric mixture, which was used in next step without isolation of both isomers.

To a stirred solution of **3** in CH_2_Cl_2_ (0.025 M) was added HBF_4_ (42% aq., 4.0 equiv) at 0 °C and stirred for 5 min at the same temperature. After that, ^t^BuNO_2_ (8.0 equiv) was added at 0 °C, which was warmed to room temperature and stirred for 2 h. The reaction was quenched by the addition of water at 0 °C. The reaction mixture was extracted with CH_2_Cl_2_, dried over Na_2_SO_4_ and filtered. After the removal of solvent by evaporation at less than room temperature, the crude product was dissolved in CHCl_3_ (0.13 M) and stirred at 60 °C for 30 min. After the removal of the solvent by evaporation, crude product was obtained, which was purified by recrystallization (hexane/toluene) to give **4**.

##### (*R*)-6-(2-(ethyl(nitroso)amino)naphthalen-1-yl)benzo[*b*]naphtho [2,3-*d*]bromol-5-ium Tetrafluoroborate (**4a**)

Pink solid, 9.9 mg, 0.017 mmol, 9% yield (from **2**).

m.p. = 156–158 °C; ^1^H-NMR (400 MHz, CHLOROFORM-D) δ 8.73 (s, 1H), 8.54 (d, *J* = 8.5 Hz, 1H), 8.37 (d, *J* = 8.5 Hz, 1H), 8.32 (d, *J* = 1.6 Hz, 1H), 8.22 (d, *J* = 8.1 Hz, 1H), 8.13 (d, *J* = 8.1 Hz, 1H), 7.85–7.89 (m, 1H), 7.70–7.79 (m, 3H), 7.64–7.68 (m, 1H), 7.55–7.59 (m, 1H), 7.38–7.45 (m, 2H), 7.04 (dd, *J* = 8.5, 0.9 Hz, 1H), 4.02 (qd, *J* = 14.0 Hz, 7.2 Hz, 1H), 3.79 (qd, *J* = 14.0 Hz, 7.2 Hz, 1H), 1.03 (t, *J* = 7.2 Hz, 3H); ^13^C-NMR (101 MHz, CHLOROFORM-D) δ 138.6, 138.0, 137.5, 135.3, 134.0, 133.7, 133.5, 133.21, 133.15, 132.2, 131.8, 131.3, 130.8, 129.68, 129.61, 129.58, 129.37, 129.3, 128.1, 126.8, 126.4, 126.14, 125.96, 124.5, 121.6, 42.3, 12.3 (1 peak is overlapped with the other peak); ^19^F-NMR (377 MHz, CHLOROFORM-D) δ −149.9; HRMS (ESI^+^ in MeCN) calcd for C_28_H_20_ON_2_Br^+^ [M-BF_4_^-^] 479.0754 found 479.0754; IR (KBr) ν 2963, 1458, 1261, 1019, 800 cm^−1^; [α]^20^_D_ = −52.2 (*c =* 0.2, CHCl_3_).

##### (*R*)-6-(2-(ethyl(nitroso)amino)naphthalen-1-yl)benzo[*b*]naphtho [2,3-*d*]iodol-5-ium Tetrafluoroborate (**4b**)

Pink solid, 10.6 mg, 0.017 mmol, 5% yield (from **2**).

m.p. = 160–162 °C; ^1^H-NMR (400 MHz, CHLOROFORM-D) δ 8.67 (d, *J* = 9.6 Hz, 1H), 8.61 (s, 1H), 8.34 (d, *J* = 8.8 Hz, 1H), 8.25 (dd, *J* = 7.9, 1.5 Hz, 1H), 8.14 (d, *J* = 8.4 Hz, 2H), 7.82 (td, *J* = 7.5, 0.6 Hz, 1H), 7.65–7.70 (m, 4H), 7.45–7.50 (m, 1H), 7.40–7.44 (m, 1H), 7.23–7.26 (m, 1H), 7.13 (dd, *J* = 8.6, 0.5 Hz, 1H), 3.76 (qd, *J* = 13.6 Hz, 7.2 Hz, 1H), 3.65 (qd, *J* = 13.6 Hz, 7.2 Hz, 1H), 0.91 (t, *J* = 7.2 Hz, 3H); ^13^C-NMR (101 MHz, CHLOROFORM-D) δ 138.7, 138.0, 137.5, 135.3, 134.0, 133.7, 133.5, 133.22, 133.15, 132.16, 131.8, 131.3, 130.8, 129.7, 129.61, 129.59, 129.4, 129.3, 128.1, 126.8, 126.4, 126.14, 125.96, 124.5, 121.6, 42.3, 12.3 (1 peak is overlapped with the other peak); ^19^F-NMR (377 MHz, CHLOROFORM-D) δ −149.3; HRMS (ESI^+^ in MeCN) calcd for C_28_H_20_ON_2_I^+^ [M-BF_4_^-^] 527.0615 found 527.0603; IR (KBr) ν 2963, 1559, 1261, 1020, 800 cm^−1^; [α]^20^_D_ = −20.3 (*c =* 0.2, CHCl_3_).

##### (*R*)-6-(2-(ethyl(nitroso)amino)naphthalen-1-yl)benzo[*b*]naphtho [2,3-*d*]chlorol-5-ium Tetrafluoroborate (**4c**)

Pink solid, 7.6 mg, 0.015 mmol, 9% yield (from **2**).

m.p. = 152–154 °C; ^1^H-NMR (400 MHz, CHLOROFORM-D) δ 8.83 (s, 1H), 8.53 (d, *J* = 8.8 Hz, 1H), 8.36–8.41 (m, 2H), 8.27 (d, *J* = 8.2 Hz, 1H), 8.12 (d, *J* = 8.2 Hz, 1H), 7.87–7.92 (m, 1H), 7.76–7.82 (m, 3H), 7.59–7.67 (m, 2H), 7.51 (dd, *J* = 8.2, 0.5 Hz, 1H), 7.40–7.45 (m, 1H), 7.02 (d, *J* = 8.5 Hz, 1H), 4.08 (qd, *J* = 14.0 Hz, 6.8 Hz, 1H), 3.85 (qd, *J* = 14.0 Hz, 6.8 Hz, 1H), 1.06 (t, *J* = 6.8 Hz, 3H); ^13^C-NMR (101 MHz, CHLOROFORM-D) δ 140.6, 139.0, 138.6, 134.1, 133.8, 133.6, 133.06, 133.03, 132.4, 132.1, 131.5, 130.00, 129.99, 129.89, 129.7, 129.28, 129.25, 128.0, 127.4, 126.8, 125.44, 125.31, 124.6, 123.99, 123.86, 121.4, 42.4, 12.3; ^19^F-NMR (377 MHz, CHLOROFORM-D) δ −151.2; HRMS (ESI^+^ in MeCN) calcd for C_28_H_20_ON_2_Cl^+^ [M-BF_4_^-^] 435.1259 found 435.1259; IR (KBr) ν 3068, 1458, 1174, 1062, 831 cm^−1^; [α]^20^_D_ = −17.4 (*c =* 0.2, CHCl_3_).

#### 3.2.3. Synthesis of **4d**

To a stirred solution of **4a** (13.0 mg, 0.023 mmol, 1.0 equiv) in CHCl_3_ (3 mL) and water (0.2 mL) was added NaCl (1.3 mg, 0.023 mmol, 10 equiv) at room temperature and stirred for 35 min at the same temperature. The reaction was quenched by the addition of water. The reaction mixture was extracted with CH_2_Cl_2_, washed with brine, dried over Na_2_SO_4_ and filtered. After the removal of solvent by evaporation to give **4d**.

##### (*R*)-6-(2-(ethyl(nitroso)amino)naphthalen-1-yl)benzo[*b*]naphtho [2,3-*d*]bromol-5-ium chloride (**4d**)

Yellow solid, 3.0 mg, 0.006 mmol, 25% yield.

m.p. = 137–139 °C; ^1^H-NMR (400 MHz, CHLOROFORM-D) δ 9.63 (dd, *J* = 8.8, 0.7 Hz, 1H), 8.64 (s, 1H), 8.33 (d, *J* = 8.5 Hz, 1H), 8.27 (dd, *J* = 7.7, 1.7 Hz, 1H), 8.15 (d, *J* = 8.2 Hz, 1H), 8.11 (d, *J* = 8.2 Hz, 1H), 7.83–7.87 (m, 1H), 7.68–7.75 (m, 3H), 7.63–7.67 (m, 1H), 7.47–7.51 (m, 1H), 7.38–7.42 (m, 1H), 7.29 (d, *J* = 8.7 Hz, 1H), 7.09 (dd, *J* = 8.5, 0.8 Hz, 1H), 3.94 (qd, *J* = 14.0 Hz, 7.2 Hz, 1H), 3.70 (qd, *J* = 14.0 Hz, 7.2 Hz, 1H), 0.99 (t, *J* = 7.2 Hz, 3H); ^13^C-NMR (101 MHz, CHLOROFORM-D) δ 139.8, 139.1, 138.7, 135.3, 133.7, 133.5, 133.2, 133.0, 132.2, 131.7, 131.5, 131.3, 130.9, 129.3, 129.16, 129.12, 129.09, 128.95, 128.4, 128.0, 127.4, 126.2, 125.1, 124.93, 124.83, 121.94, 42.3, 12.3; HRMS (ESI^+^ in MeCN) calcd for C_28_H_20_ON_2_Br^+^ [M-Cl] 479.0754 found 479.0748; IR (KBr) ν 2962, 1560, 1261, 1019, 801 cm^−1^; [α]^20^_D_ = −22.1 (*c =* 0.2, CHCl_3_).

### 3.3. General Procedure for Mannich Reaction of Imines with Active Methylenes

Imine **5** (1.0 equiv), **4** (appropriate amount), and K_2_CO_3_ (2.0 equiv) were put into the test tube, which was cooled to –30 °C. After 5 min, the solvent (0.036 M) was slowly added and stirred for 5 min, which was added **6** (2.5 equiv) at the same temperature. The test tube was warmed to the appropriate temperature and stirred for the scheduled time, which was quenched by the adition of water. The reaction mixture was extracted with CH_2_Cl_2_, dried over Na_2_SO_4_ and filtered. After the removal of solvent by evaporation, the crude product was obtained, which was purified by column chromatography (Silica-gel, hexane/ethyl acetate) to give **7**.

#### 3.3.1. dimethyl (*R*)-2-(1-benzyl-3-((*tert*-butoxycarbonyl)amino)-2-oxoindolin-3-yl)malonate (**7a**)

White solid, 12.9 mg, 0.028 mmol, 55% yield, 62% ee.

m.p. = 114–116 °C; ^1^H-NMR (400 MHz, CHLOROFORM-D) δ 7.41–7.43 (m, 2H), 7.31–7.38 (m, 3H), 7.24–7.28 (m, 1H), 7.19 (td, *J* = 7.8, 1.3 Hz, 1H), 6.99 (td, *J* = 7.8, 1.3 Hz, 1H), 6.71 (d, *J* = 7.8 Hz, 1H), 6.42 (s, 1H), 5.01 (d, *J* = 15.8 Hz, 1H), 4.87 (d, *J* = 15.8 Hz, 1H), 3.96 (s, 1H), 3.70 (s, 3H), 3.69 (s, 3H), 1.31 (s, 9H); ^13^C-NMR (101 MHz, CHLOROFORM-D) δ 174.0, 166.3, 166.1, 153.9, 143.1, 135.6, 129.5, 128.7, 127.9, 127.6, 123.9, 122.7, 109.3, 80.5, 60.9, 55.3, 52.98, 52.95, 44.4, 28.1; HRMS (ESI^+^ in MeCN) calcd for C_25_H_28_O_7_N_2_Na [M + Na^+^] 491.1789 found 491.1783; IR (KBr) ν 2964, 1261, 1094, 800, 702 cm^−1^; [α]^20^_D_ = +9.6 (*c =* 0.2, CHCl_3_); HPLC (CHIRALPAK IA column, hexane/2-propanol = 80/20, flow rate 1.0 mL/min, 25 °C, 254 nm) first peak: t_R_ = 8.3 min (major), second peak: t_R_ = 11.4 min (minor).

#### 3.3.2. dimethyl (*R*)-2-(3-((*tert*-butoxycarbonyl)amino)-1-methyl-2-oxoindolin-3-yl)malonate (**7b**) [48]

White solid, 15.7 mg, 0.040 mmol, 80% yield, 31% ee.

^1^H-NMR (400 MHz, CHLOROFORM-D) δ 7.37 (d, *J* = 7.4 Hz, 1H), 7.32 (td, *J* = 7.7, 1.3 Hz, 1H), 7.03 (td, *J* = 7.7, 1.0 Hz, 1H), 6.83 (d, *J* = 7.4 Hz, 1H), 6.32 (s, 1H), 3.96 (s, 1H), 3.70 (s, 3H), 3.70 (s, 3H), 3.25 (s, 3H), 1.27 (s, 9H); ^13^C-NMR (101 MHz, CHLOROFORM-D) δ 173.8, 166.4, 166.1, 153.8, 144.1, 129.7, 127.7, 123.9, 122.7, 108.3, 80.4, 60.8, 55.2, 53.0, 28.1, 26.6; HPLC (CHIRALCEL OD-H column, hexane/2-propanol = 80/20, flow rate 1.0 mL/min, 25 °C, 254 nm) first peak: t_R_ = 6.8 min (major), second peak: t_R_ = 10.7 min (minor).

#### 3.3.3. dimethyl (*R*)-2-(3-((*tert*-butoxycarbonyl)amino)-2-oxo-1-phenylindolin-3-yl)malonate (**7c**)

White solid, 19.9 mg, 0.044 mmol, 88% yield, 55% ee.

m.p. = 129–131 °C; ^1^H-NMR (400 MHz, CHLOROFORM-D) δ 7.48–7.53 (m, 4H), 7.38–7.45 (m, 2H), 7.23 (td, *J* = 7.8, 1.3 Hz, 1H), 7.06 (td, *J* = 7.5, 0.9 Hz, 1H), 6.80 (d, *J* = 8.0 Hz, 1H), 6.37 (s, 1H), 4.14 (s, 1H), 3.72 (s, 3H), 3.70 (s, 3H), 1.32 (s, 9H); ^13^C-NMR (101 MHz, CHLOROFORM-D) δ 173.4, 166.5, 166.1, 153.9, 144.5, 134.4, 129.6, 128.1, 127.4, 126.6, 124.2, 123.1, 109.5, 80.6, 60.8, 55.6, 53.06, 53.00, 28.2; HRMS (ESI^+^ in MeCN) calcd for C_24_H_26_O_7_N_2_Na [M + Na^+^] 477.1632 found 477.1627; IR (KBr) ν 2963, 1261, 1018, 798, 702 cm^−1^; [α]^20^_D_ = +17.1 (*c =* 0.2, CHCl_3_); HPLC (CHIRALPAK IA column, hexane/2-propanol = 80/20, flow rate 1.0 mL/min, 25 °C, 254 nm) first peak: t_R_ = 7.2 min (major), second peak: t_R_ = 8.2 min (minor).

#### 3.3.4. dimethyl (*R*)-2-(1-benzyl-3-((*tert*-butoxycarbonyl)amino)-4-chloro-2-oxoindolin-3-yl)malonate (**7d**)

White solid, 14.3 mg, 0.028 mmol, 56% yield, racemic.

m.p. = 140–142 °C; ^1^H-NMR (400 MHz, CHLOROFORM-D) δ 7.48–7.41 (m, 2H), 7.34–7.31 (m, 2H), 7.28–7.24 (m, 1H), 7.10 (t, *J* = 8.0 Hz, 1H), 6.90 (dd, *J* = 8.3, 0.8 Hz, 1H), 6.79 (s, 1H), 6.57 (d, *J* = 7.8 Hz, 1H), 5.10 (d, *J* = 16.1 Hz, 1H), 4.81 (d, *J* = 16.1 Hz, 1H), 4.44 (s, 1H), 3.86 (s, 3H), 3.49 (s, 3H), 1.29 (s, 9H); ^13^C-NMR (101 MHz, CHLOROFORM-D) δ 173.4, 166.9, 165.4, 153.2, 146.1, 135.2, 130.8, 130.0, 128.7, 127.57, 127.52, 123.5, 107.8, 80.5, 61.2, 53.8, 53.6, 52.8, 44.7, 28.0; HRMS (ESI^+^ in MeCN) calcd for C_25_H_27_O_7_N_2_ClNa [M + Na^+^] 525.1399 found 525.1403; IR (KBr) ν 2980, 1729, 1498, 1257, 912 cm^−1^; HPLC (CHIRALPAK IA column, hexane/2-propanol = 80/20, flow rate 1.0 mL/min, 25 °C, 220 nm) first peak: t_R_ = 7.2 min, second peak: t_R_ = 11.0 min.

#### 3.3.5. dimethyl (*R*)-2-(1-benzyl-3-((*tert*-butoxycarbonyl)amino)-5-methyl-2-oxoindolin-3-yl)malonate (**7e**)

White solid, 21.2 mg, 0.044 mmol, 88% yield, 60 % ee.

m.p. = 128–130 °C; ^1^H-NMR (400 MHz, CHLOROFORM-D) δ 7.39–7.41 (m, 2H), 7.27–7.34 (m, 3H), 7.17 (d, *J* = 0.9 Hz, 1H), 6.97 (dd, *J* = 8.0, 0.9 Hz, 1H), 6.58 (d, *J* = 8.0 Hz, 1H), 6.43 (s, 1H), 4.97 (d, *J* = 16.5 Hz, 1H), 4.86 (d, *J* = 16.5 Hz, 1H), 3.93 (s, 1H), 3.70 (s, 3H), 3.69 (s, 3H), 2.26 (s, 3H), 1.32 (s, 9H); ^13^C-NMR (101 MHz, CHLOROFORM-D) δ 174.0, 166.4, 166.2, 153.9, 140.7, 135.8, 132.2, 129.8, 128.7, 127.9, 127.5, 124.7, 109.1, 80.4, 61.0, 55.3, 52.96, 52.90, 44.4, 28.2, 21.1; HRMS (ESI^+^ in MeCN) calcd for C_26_H_30_O_7_N_2_K [M + K^+^] 521.1685 found 521.1676; IR (KBr) ν 2963, 1719, 1497, 1261, 803 cm^−1^; [α]^20^_D_ = +19.8 (*c =* 0.2, CHCl_3_); HPLC (CHIRALPAK IA column, hexane/2-propanol = 80/20, flow rate 1.0 mL/min, 25 °C, 254 nm) first peak: t_R_ = 7.8 min (major), second peak: t_R_ = 11.0 min (minor).

#### 3.3.6. dimethyl (*R*)-2-(1-benzyl-3-((*tert*-butoxycarbonyl)amino)-5-chloro-2-oxoindolin-3-yl)malonate (**7f**)

White solid, 22.9 mg, 0.046 mmol, 91% yield, 73% ee.

m.p. = 125–127 °C; ^1^H-NMR (400 MHz, CHLOROFORM-D) δ 7.38–7.40 (m, 2H), 7.31–7.36 (m, 3H), 7.25–7.29 (m, 1H), 7.15 (dd, *J* = 8.3, 2.1 Hz, 1H), 6.60 (d, *J* = 8.3 Hz, 1H), 6.41 (s, 1H), 4.96 (d, *J* = 16.3 Hz, 1H), 4.89 (d, *J* = 16.3 Hz, 1H), 3.92 (s, 1H), 3.74 (s, 3H), 3.70 (s, 3H), 1.34 (s, 9H); ^13^C-NMR (101 MHz, CHLOROFORM-D) δ 173.6, 166.2, 165.8, 153.8, 141.8, 135.2, 129.5, 128.8, 128.1, 127.7, 127.5, 124.5, 110.3, 80.8, 60.8, 55.2, 53.15, 53.08, 44.6, 28.1; HRMS (ESI^+^ in MeCN) calcd for C_25_H_27_O_7_N_2_ClNa [M + Na^+^] 525.1399 found 525.1396; IR (KBr) ν 2963, 1734, 1261, 1020, 801 cm^−1^; [α]^20^_D_ = +15.7 (*c =* 0.2, CHCl_3_); HPLC (CHIRALPAK IA column, hexane/2-propanol = 80/20, flow rate 1.0 mL/min, 25 °C, 254 nm) first peak: t_R_ = 8.4 min (major), second peak: t_R_ = 10.8 min (minor).

#### 3.3.7. dimethyl (*R*)-2-(1-benzyl-6-bromo-3-((*tert*-butoxycarbonyl)amino)-2-oxoindolin-3-yl)malonate (**7g**)

White solid, 26.9 mg, 0.049 mmol, 98% yield, 56% ee.

m.p. = 130–132 °C; ^1^H-NMR (400 MHz, CHLOROFORM-D) δ 7.39–7.41 (m, 2H), 7.33–7.37 (m, 2H), 7.28–7.31 (m, 1H), 7.24 (d, *J* = 8.1 Hz, 1H), 7.13 (dd, J = 8.1, 1.8 Hz, 1H), 6.84 (d, *J* = 1.8 Hz, 1H), 6.39 (s, 1H), 4.96 (d, *J* = 16.3 Hz, 1H), 4.86 (d, *J* = 16.3 Hz, 1H), 3.93 (s, 1H), 3.72 (s, 3H), 3.68 (s, 3H), 1.33 (s, 9H); ^13^C-NMR (101 MHz, CHLOROFORM-D) δ 173.9, 166.2, 165.9, 153.8, 144.5, 135.0, 128.8, 127.8, 127.4, 126.7, 125.6, 125.2, 123.3, 112.7, 80.7, 60.5, 55.0, 53.14, 53.08, 44.5, 28.1; HRMS (ESI^+^ in MeCN) calcd for C_25_H_27_O_7_N_2_BrNa [M + Na^+^] 569.0894 found 569.0892; IR (KBr) ν 2962, 1735, 1261, 1020, 800 cm^−1^; [α]^20^_D_ = +14.5 (*c =* 0.2, CHCl_3_); HPLC (CHIRALPAK IA column, hexane/2-propanol = 80/20, flow rate 1.0 mL/min, 25 °C, 254 nm) first peak: t_R_ = 7.5 min (major), second peak: t_R_ = 10.0 min (minor).

#### 3.3.8. dimethyl (*R*)-2-(1-benzyl-3-((*tert*-butoxycarbonyl)amino)-7-chloro-2-oxoindolin-3-yl)malonate (**7h**)

White solid, 20.9 mg, 0.042 mmol, 83% yield, 80% ee.

m.p. = 150–152 °C; ^1^H-NMR (400 MHz, CHLOROFORM-D) δ 7.40 (d, *J* = 7.4 Hz, 2H), 7.29–7.33 (m, 2H), 7.22–7.27 (m, 2H), 7.18 (dd, *J* = 8.2, 1.1 Hz, 1H), 6.94 (t, *J* = 7.8 Hz, 1H), 6.54 (s, 1H), 5.38 (d, *J* = 16.3 Hz, 1H), 5.33 (d, *J* = 16.3 Hz, 1H), 3.86 (s, 1H), 3.72 (s, 3H), 3.71 (s, 3H), 1.33 (s, 9H); ^13^C-NMR (101 MHz, CHLOROFORM-D) δ 174.8, 166.0, 165.9, 153.8, 139.3, 137.5, 132.2, 130.9, 128.4, 127.01, 126.90, 123.6, 122.2, 115.7, 80.8, 60.5, 55.3, 53.13, 53.08, 45.6, 28.1; HRMS (ESI^+^ in MeCN) calcd for C_25_H_27_O_7_N_2_ClNa [M + Na^+^] 525.1399 found 525.1393; IR (KBr) ν 2962, 1734, 1457, 1261, 802 cm^−1^; [α]^20^_D_ = +6.7 (*c =* 0.2, CHCl_3_); HPLC (CHIRALPAK IA column, hexane/2-propanol = 80/20, flow rate 1.0 mL/min, 25 °C, 254 nm) first peak: t_R_ = 7.8 min (major), second peak: t_R_ = 11.3 min (minor).

#### 3.3.9. dimethyl (*R*)-2-(1-benzyl-3-(((benzyloxy)carbonyl)amino)-2-oxoindolin-3-yl)malonate (**7i**)

White solid, 22.6 mg, 0.041 mmol, 90% yield, 47% ee

m.p. = 137–139 °C; ^1^H-NMR (400 MHz, CHLOROFORM-D) δ 7.28–7.39 (m, 2H), 7.25–7.35 (m, 9H), 7.20 (td, *J* = 7.8, 1.2 Hz, 1H), 7.00 (td, *J* = 7.8, 1.2 Hz, 1H), 6.81 (s, 1H), 6.68 (d, *J* = 7.7 Hz, 1H), 4.95–5.03 (m, 4H), 3.93 (s, 1H), 3.73 (s, 3H), 3.67 (s, 3H); ^13^C-NMR (101 MHz, CHLOROFORM-D) δ 173.6, 166.3, 166.0, 154.4, 143.2, 135.7, 135.5, 129.9, 128.7, 128.5, 128.20, 128.15, 127.6, 127.4, 127.2, 123.9, 122.9, 109.6, 67.2, 60.5, 55.4, 53.14, 53.03, 44.5; HRMS (ESI^+^ in MeCN) calcd for C_28_H_26_O_7_N_2_Na [M + Na^+^] 525.1632 found 525.1633; IR (KBr) ν 2962, 1733, 1260, 1021, 799 cm^−1^; [α]^20^_D_ = +4.8 (*c =* 0.2, CHCl_3_); HPLC (CHIRALPAK IA column, hexane/2-propanol = 80/20, flow rate 1.0 mL/min, 25 °C, 254 nm) first peak: t_R_ = 20.2 min (major), second peak: t_R_ = 22.9 min (minor).

#### 3.3.10. dibenzyl (*R*)-2-(1-benzyl-3-((*tert*-butoxycarbonyl)amino)-2-oxoindolin-3-yl)malonate (**7j**)

White solid, 27.4 mg, 0.044 mmol, 88% yield, 28% ee.

m.p. = 131–133 °C; ^1^H-NMR (400 MHz, CHLOROFORM-D) δ 7.20–7.35 (m, 16H), 7.14 (td, *J* = 7.8, 1.2 Hz, 1H), 6.88 (td, *J* = 7.8, 1.2 Hz, 1H), 6.61 (d, *J* = 7.8 Hz, 1H), 6.43 (s, 1H), 5.14 (d, *J* = 14.0 Hz, 1H), 5.11 (d, *J* = 14.0 Hz, 1H), 5.06 (d, *J* = 14.0 Hz, 1H), 5.04 (d, *J* = 14.0 Hz, 1H), 4.77–4.75 (m, 2H), 4.03 (s, 1H), 1.28 (s, 9H); ^13^C-NMR (101 MHz, CHLOROFORM-D) δ 173.9, 165.6, 153.8, 143.0, 135.6, 134.61, 134.59, 129.5, 128.65, 128.62, 128.54, 128.52, 128.48, 127.8, 127.5, 124.0, 122.7, 109.3, 80.4, 67.98, 67.91, 61.0, 55.5, 44.2, 28.1; HRMS (ESI^+^ in MeCN) calcd for C_37_H_36_O_7_N_2_Na [M + Na^+^] 643.2415 found 643.2411; IR (KBr) ν 2926, 1734, 1507, 1168, 741 cm^−1^; [α]^20^_D_ = −2.6 (*c =* 0.2, CHCl_3_); HPLC (CHIRALPAK IA column, hexane/2-propanol = 80/20, flow rate 1.0 mL/min, 25 °C, 254 nm) first peak: t_R_ = 10.1 min (major), second peak: t_R_ = 17.2 min (minor).

#### 3.3.11. diethyl (*R*)-2-(1-benzyl-3-((*tert*-butoxycarbonyl)amino)-2-oxoindolin-3-yl)malonate (**7k**) [47]

White solid, 20.3 mg, 0.041 mmol, 82% yield, 3% ee.

^1^H-NMR (400 MHz, CHLOROFORM-D) δ 7.38–7.44 (m, 3H), 7.31–7.35 (m, 2H), 7.24–7.28 (m, 1H) 7.18 (td, *J* = 7.7, 1.3 Hz, 1H), 6.98 (td, *J* = 7.8, 1.3 Hz, 1H), 6.70 (d, *J* = 7.8 Hz, 1H), 6.46 (s, 1H), 5.00 (d, *J* = 15.9 Hz, 1H), 4.86 (d, *J* = 15.9 Hz, 1H), 4.20–4.12 (m, 4H), 3.91 (s, 1H), 1.31 (s, 9H), 1.187 (t, *J* = 7.2 Hz, 3H), 1.185 (t, *J* = 7.2 Hz, 3H); ^13^C-NMR (101 MHz, CHLOROFORM-D) δ 174.1, 165.9, 165.7, 153.9, 143.2, 135.7, 129.5, 128.7, 128.0, 127.6, 124.1, 122.6, 109.2, 80.4, 62.15, 62.09, 60.9, 55.6, 44.4, 28.1, 13.85, 13.80; HPLC (CHIRALPAK IA column, hexane/2-propanol = 80/20, flow rate 1.0 mL/min, 25 °C, 254 nm) first peak: t_R_ = 7.0 min (major), second peak: t_R_ = 9.9 min (minor).

#### 3.3.12. *tert*-butyl (*S*)-(1-benzyl-3-(2,4-dioxopentan-3-yl)-2-oxoindolin-3-yl)carbamate (**7m**) [48]

White solid, 11.2 mg, 0.026 mmol, 51% yield, 61% ee.

^1^H-NMR (400 MHz, CHLOROFORM-D) δ 7.42 (d, *J* = 8.8 Hz, 2H), 7.35 (t, *J* = 8.8 Hz, 2H), 7.26–7.28 (m, 2H), 7.18 (td, J = 7.8, 1.2 Hz, 1H), 6.98 (td, *J* = 7.8, 0.9 Hz, 1H), 6.72 (d, J = 7.8 Hz, 1H), 6.58 (s, 1H), 5.05 (d, *J* = 17.1 Hz, 1H), 4.82 (d, *J* = 17.1 Hz, 1H), 4.07 (s, 1H), 2.31 (s, 3H), 2.17 (s, 3H), 1.31 (s, 9H); ^13^C-NMR (101 MHz, CHLOROFORM-D) δ 201.7, 201.3, 174.2, 153.9, 142.5, 135.6, 129.5, 128.8, 128.3, 127.7, 127.5, 123.6, 122.9, 109.5, 80.5, 68.6, 62.8, 44.4, 32.33, 32.26, 28.1; HRMS (ESI^+^ in MeCN) calcd for C_25_H_28_O_7_N_2_Na [M + Na^+^] 491.1789 found 491.1783; HPLC (CHIRALPAK IC column, hexane/2-propanol = 95/5, flow rate 1.0 mL/min, 25 °C, 210 nm) first peak: t_R_ = 196.5 min (major), second peak: tR = 172.7 min (minor), or (CHIRALPAK IA column, hexane/2-propanol = 80/20, flow rate 1.0 mL/min, 25 °C, 254 nm) first peak: t_R_ = 7.4 min (minor), second peak: t_R_ = 15.8 min (major).

#### 3.3.13. *tert*-butyl (*S*)-(1-benzyl-3-(1,3-dioxo-1,3-diphenylpropan-2-yl)-2-oxoindolin-3-yl)carbamate (**7n**) [58]

White solid, 20.3 mg, 0.021 mmol, 72% yield, 5% ee.

^1^H-NMR (400 MHz, CHLOROFORM-D) δ 7.79–7.81 (m, 2H), 7.65–7.66 (m, 2H), 7.47–7.54 (m, 2H), 7.30–7.39 (m, 10H), 7.16 (td, *J* = 7.7, 1.1 Hz, 1H), 7.07 (s, 1H), 6.90 (td, *J* = 7.7, 1.1 Hz, 1H), 6.75 (d, *J* = 7.7 Hz, 1H), 5.80 (s, 1H), 4.86 (d, *J* = 17.1 Hz, 1H), 4.69 (d, *J* = 17.1 Hz, 1H), 1.31 (s, 9H); ^13^C-NMR (101 MHz, CHLOROFORM-D) δ 192.1, 191.8, 174.4, 153.8, 142.4, 136.7, 136.5, 136.0, 133.8, 133.7, 129.2, 128.8, 128.7, 128.52, 128.46 127.61, 127.55, 125.1, 122.9, 109.1, 80.1, 64.1, 55.9, 44.2, 28.2; HPLC (CHIRALPAK IA column, hexane/2-propanol = 80/20, flow rate 1.0 mL/min, 25 °C, 254 nm) first peak: t_R_ = 10.2 min (minor), second peak: t_R_ = 14.5 min (major).

### 3.4. Synthesis of **8**

Dibenzo[*b*,*d*]bromol-5-ium chloride [26] (1.0 equiv) and NaBF*_4_* (10 equiv) were dissolved in CHCl*_3_* (0.025 M) and H*_2_*O (0.05 M), which was stirred for 1.5 h at room temperature. The reaction was quenched by the addition of water. The reaction mixture was extracted with CH_2_Cl_2_, washed with brine, dried over Na_2_SO_4_ and filtered. After the removal of solvent by evaporation to give crude **8**, which was washed by hexane to give ***8***.

#### dibenzo[*b*,*d*]bromol-5-ium Tetrafluoroborate (**8**) [28]

White solid, 11.1 mg, 0.035 mmol, 70% yield.

^1^H-NMR (400 MHz, DMSO-D_6_) δ 8.59 (d, *J* = 8.5 Hz, 2H), 8.49 (d, *J* = 8.5 Hz, 2H), 7.95 (t, *J* = 8.5 Hz, 2H), 7.83–7.87 (dd, *J* = 8.5 Hz, *J* = 1.6 Hz, 2H); ^13^C-NMR (101 MHz, DMSO-D_6_) δ 136.5, 135.3, 131.7, 131.1, 126.2, 125.5; ^19^F-NMR (377 MHz, DMSO-D_6_) δ −148.42.

### 3.5. Calculation Methods

All calculations were carried out with the Gaussian16 (Revision B.01). The M06-2X functional was employed, which was appropriate for the evaluation of weak non-covalent interactions. The 6–31G(d) was used as basis sets for all atoms. The calculations were carried out in gas phase at 25 °C. The nature of the calculated minimum was confirmed by the expected number of imaginary frequencies (N_imag_ = 0).

## 4. Conclusions

In conclusion, we have developed a chiral halonium salt with the *N*-nitrosamine moiety, which was applied to the Mannich reaction of malonic esters with ketimines to afford corresponding products with up to 80% ee. The catalyst screening, ^1^H NMR study, and DFT calculation of the catalyst suggested that the halogen bond from the halonium salt moiety and the *N*-nitrosamine part had a crucial effect on the product’s enantioselectivity. To the best of our knowledge, this is the first catalytic application of *N*-nitrosamines. The development of a novel reaction, which is to be achieved by the cooperative activation of both the halonium salt and the *N*-nitrosamine part is ongoing.

## Figures and Tables

**Table 1:** Reaction condition optimizations.

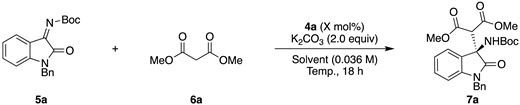					
Entry	Solvent	Temp. (°C)	X (mol%)	Yield (%) ^a^	Ee (%)
1	toluene	−20	2.5	>99	68
2	THF	−20	2.5	90	62
3	CH_2_Cl_2_	−20	2.5	97	43
4	Et_2_O	−20	2.5	92	2
5	CH_3_CN	−20	2.5	96	Rac.
6	toluene	−10	2.5	>99	36
7	toluene	−30	2.5	91	68
8	toluene	−20	1.0	92	Rac.
9	toluene	−20	5.0	91	59
10	toluene	−20	-	88	-

^a 1^H NMR yields using 1,3,5-trimethoxybenzene as an internal standard.

## Data Availability

Data are included within the manuscript or the supplementary data file. All known compounds are prepared according to the reported procedures.

## Supplementary Materials

The following supporting information can be downloaded at: https://www.mdpi.com/article/10.3390/molecules28010384/s1. The raw data for the determination of binding constants (Table S1), ^1^H, ^13^C, and ^19^F NMR charts of the catalyst and their synthetic intermediate, and the products, HPLC charts of the products, DFT calculation results.

## Sample Availability

Samples of the compounds are not available from the authors.
