# Supplementary material for: Chiral Bromonium Salt (Hypervalent Bromine(III)) with N-Nitrosamine as a Halogen-Bonding Bifunctional Catalyst
Source: Molecules. 2023 Jan 2;28(1):384. doi: 10.3390/molecules28010384 (PMC9822295; doi:10.3390/molecules28010384)
Supplement: Supplementary file 1 [file molecules-28-00384-s001.zip › molecules-2122153-supplementary.pdf]

Table S1. Results for the titration reactions shown in Figure 3 and 4.

Concentration of **4a** / M	Concentration of **5a** / M	Equivalent of **5a**	Chemical shift for H1	Chemical shift for H4
8.80E-03	0	0	8.693	7.7614
8.80E-03	0.22	25	8.695	7.7609
8.80E-03	0.44	50	8.699	7.7604
8.80E-03	0.66	75	8.701	7.7599
8.80E-03	0.88	100	8.705	7.7594
8.80E-03	1.1	125	8.708	7.7589
8.80E-03	1.32	150	8.712	7.7585
8.80E-03	1.54	175	8.716	7.758
8.80E-03	1.76	200	8.72	7.7575

Concentration of **4a** / M	Concentration of **6a** / M	Equivalent of **6a**	Chemical shift for H1	Chemical shift for H4
8.80E-03	0	0	8.692	7.7621
8.80E-03	0.22	25	8.701	7.76595
8.80E-03	0.44	50	8.703	7.76705
8.80E-03	0.66	75	8.707	7.76835
8.80E-03	0.88	100	8.708	7.7687
8.80E-03	1.1	125	8.71	7.7693
8.80E-03	1.32	150	8.712	7.77
8.80E-03	1.54	175	8.717	7.77125
8.80E-03	1.76	200	8.719	7.7717

^{1}H, ^{13}C, and ^{19}F-NMR spectra and HPLC charts.

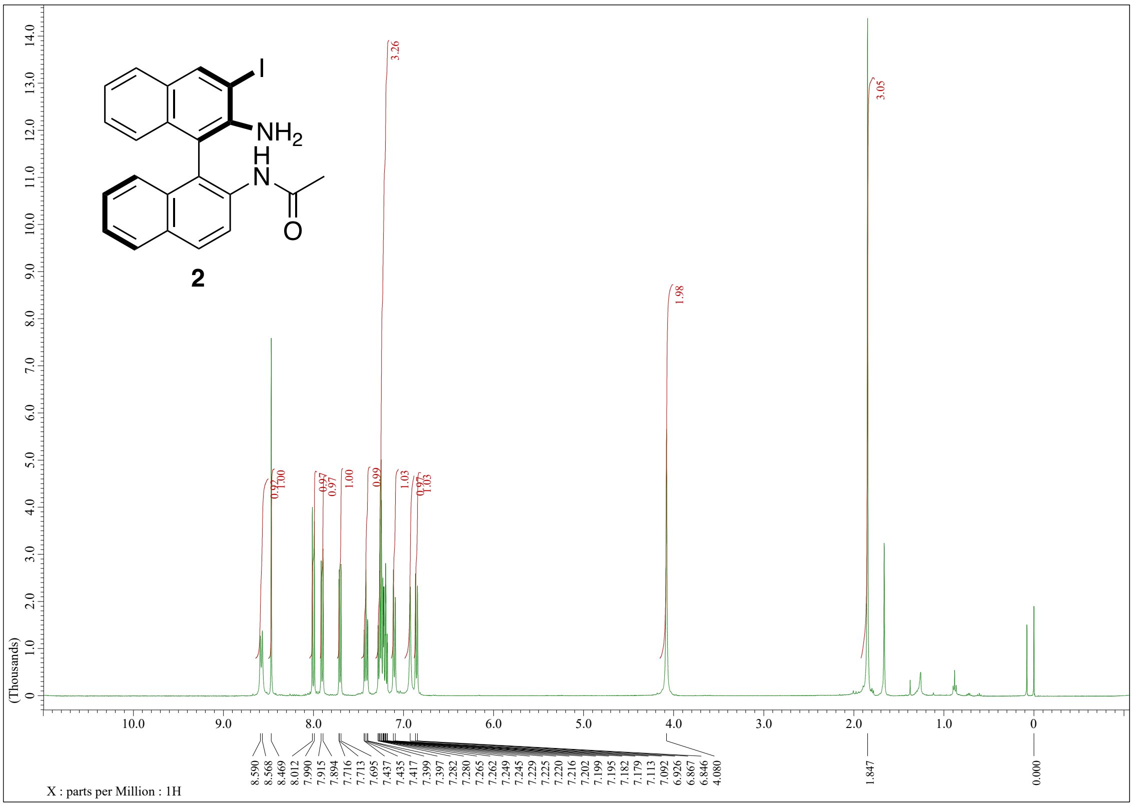

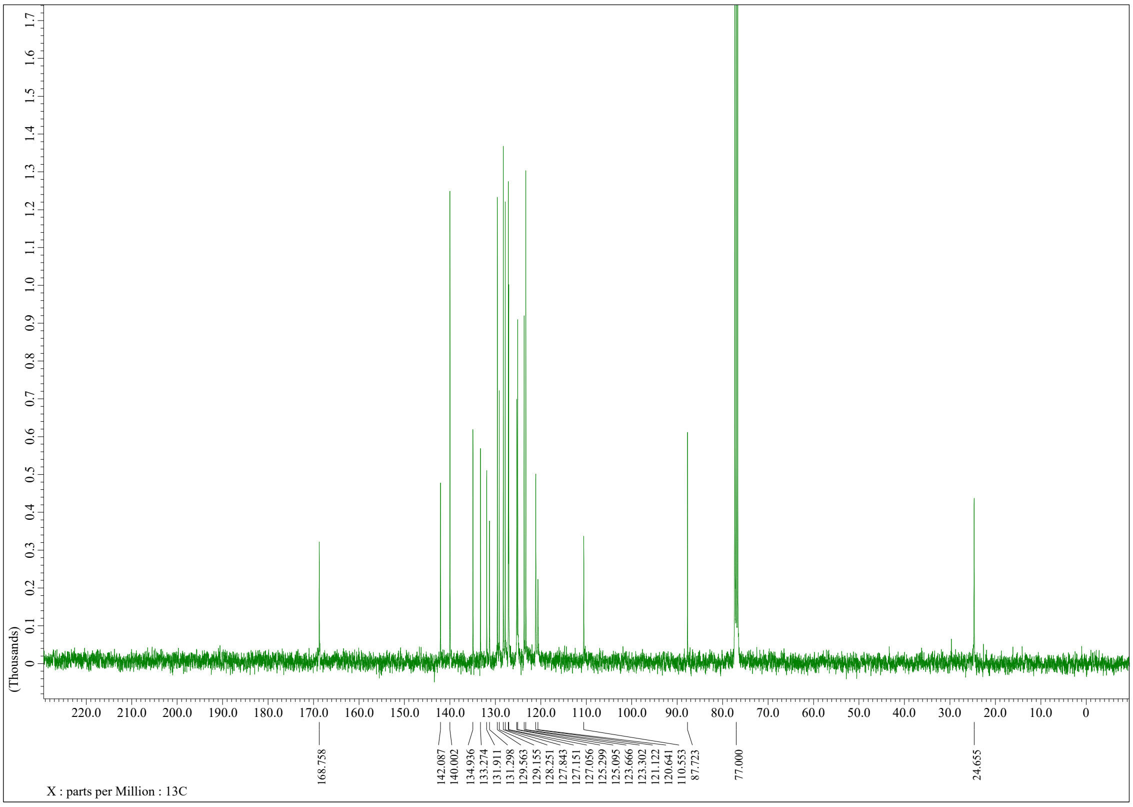

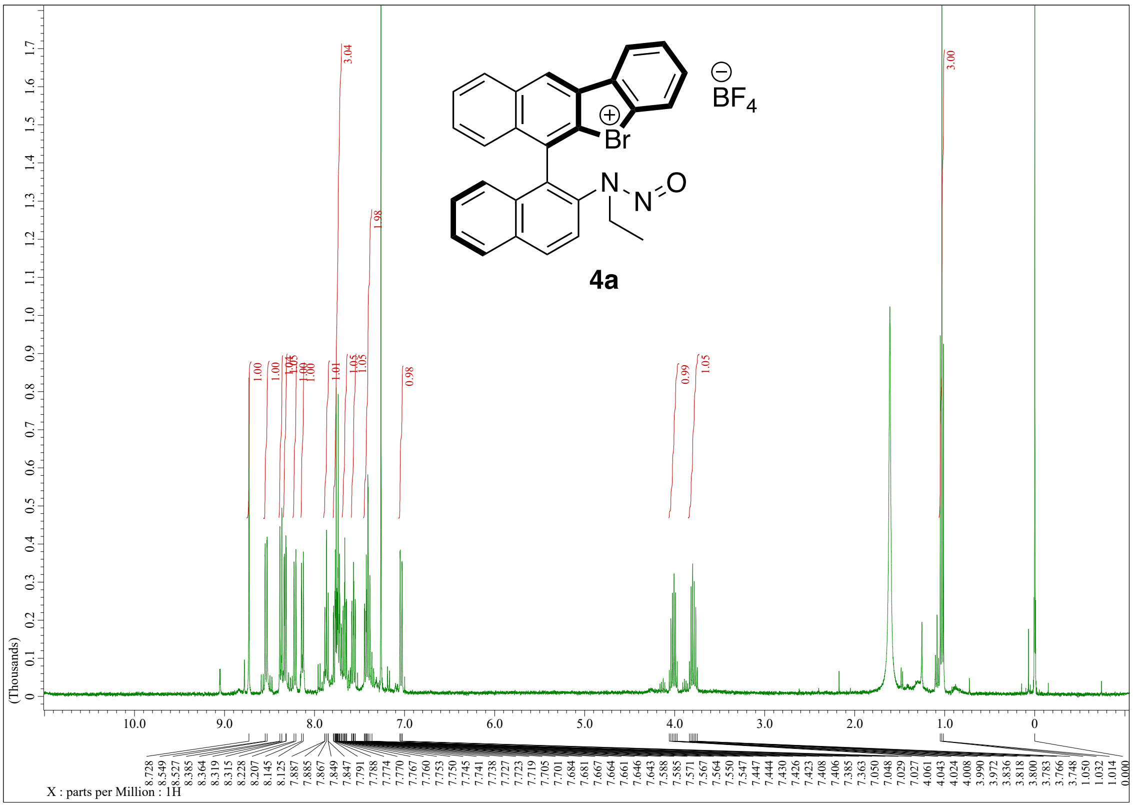

BF4
Br
N
N
O
4a
(Thousands)
1.00 1.00 1.04 1.05 1.00 1.00 1.01 3.04 1.05 1.05 1.98 0.98 0.99 1.05 3.00
8.728 8.549 8.527 8.385 8.364 8.319 8.315 8.228 8.207 8.145 8.125 7.887 7.885 7.867 7.849 7.847 7.791 7.788 7.774 7.770 7.767 7.760 7.753 7.750 7.745 7.741 7.738 7.727 7.723 7.719 7.705 7.701 7.684 7.681 7.667 7.664 7.661 7.646 7.643 7.588 7.585 7.571 7.567 7.564 7.550 7.547 7.444 7.430 7.426 7.423 7.408 7.406 7.385 7.363 7.050 7.048 7.029 7.027 4.061 4.043 4.024 4.008 3.990 3.972 3.836 3.818 3.800 3.783 3.766 3.748 1.050 1.032 1.014 0.000
X : parts per Million : 1H

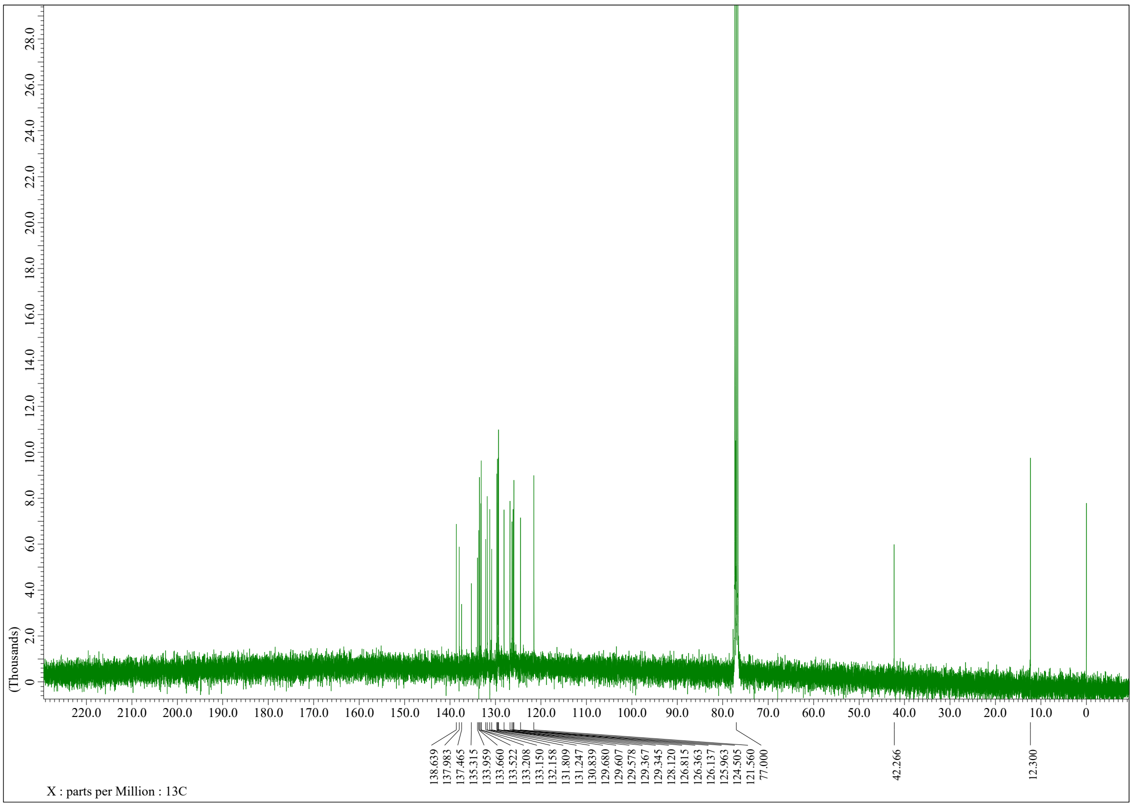

(Thousands)
138.639 137.983 137.465 135.315 133.959 133.660 133.522 133.208 133.150 132.158 131.809 131.247 130.839 129.680 129.607 129.578 129.367 129.345 128.120 126.815 126.363 126.137 125.963 124.505 121.560 77.000
42.266
12.300
X : parts per Million : 13C

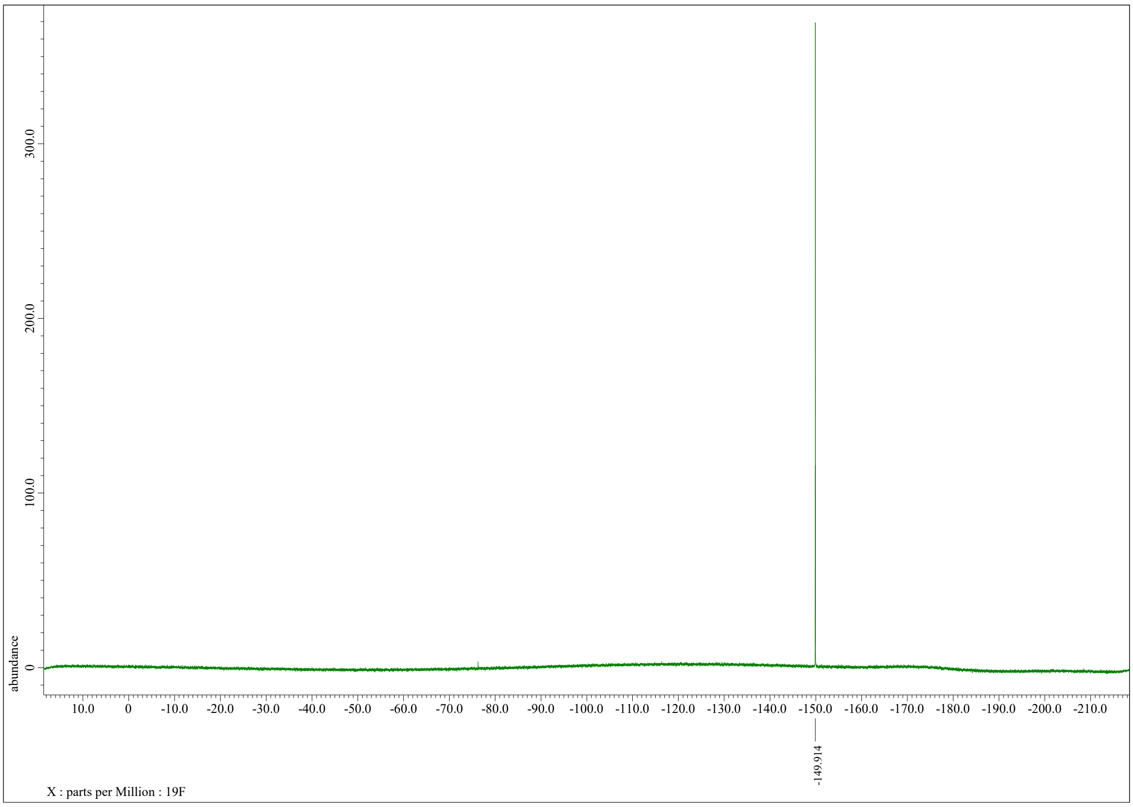

abundance
300.0
200.0
100.0
0
10.0 0 -10.0 -20.0 -30.0 -40.0 -50.0 -60.0 -70.0 -80.0 -90.0 -100.0 -110.0 -120.0 -130.0 -140.0 -150.0 -160.0 -170.0 -180.0 -190.0 -200.0 -210.0
-149.914
X : parts per Million : 19F

H-H COSY of **4a**

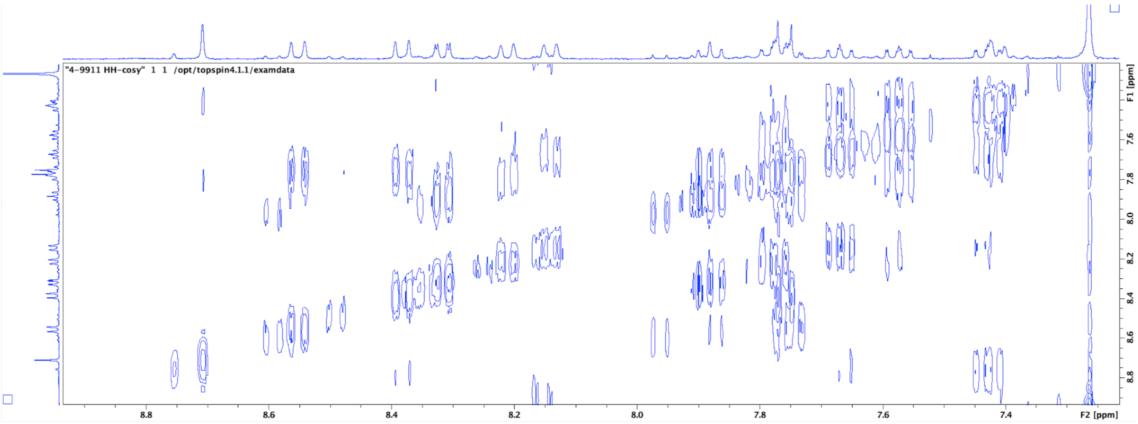

"4-9911 HH-cosy" 1 1 /opt/topspin4.1.1/examdata
F1 [ppm]
7.6
7.8
8.0
8.2
8.4
8.6
8.8
8.8 8.6 8.4 8.2 8.0 7.8 7.6 7.4
F2 [ppm]

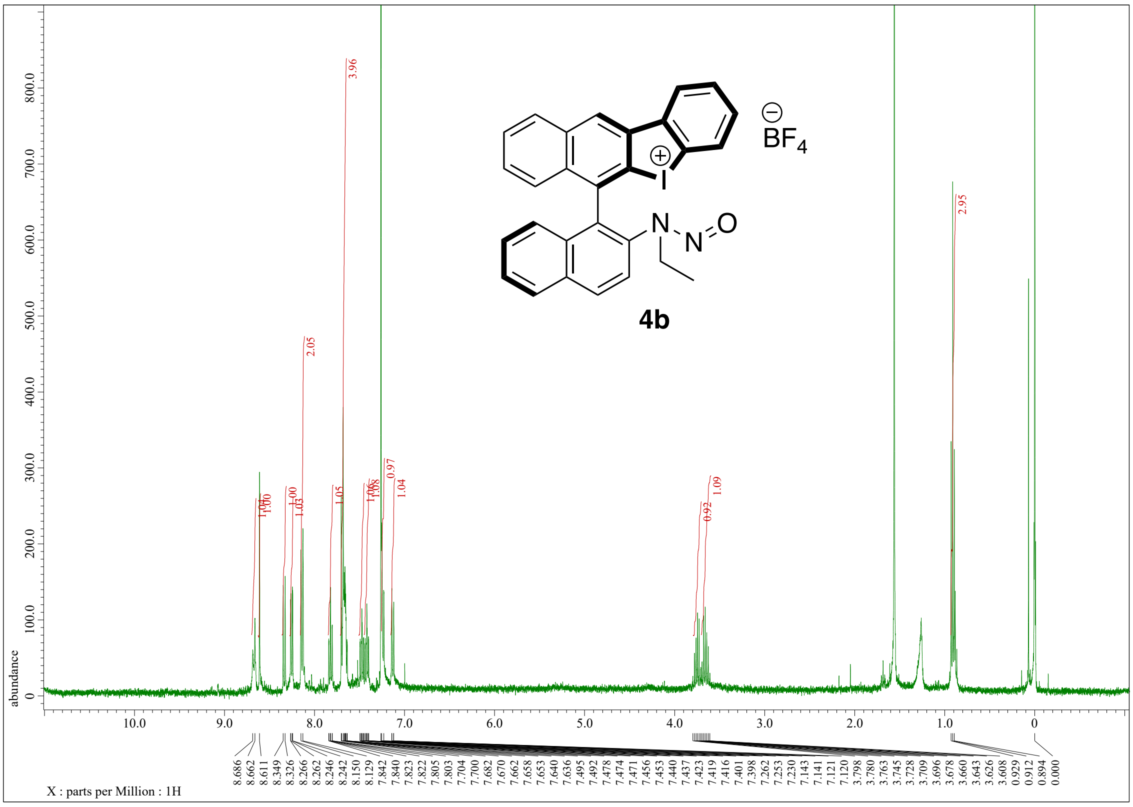
BF4
4b
abundance
X : parts per Million : 1H
8.686 8.662 8.611 8.349 8.326 8.266 8.262 8.246 8.242 8.150 8.129 7.842 7.840 7.823 7.822 7.805 7.803 7.704 7.700 7.682 7.670 7.662 7.658 7.653 7.640 7.636 7.495 7.492 7.478 7.474 7.471 7.456 7.453 7.440 7.437 7.423 7.419 7.416 7.401 7.398 7.262 7.253 7.230 7.143 7.141 7.121 7.120 3.798 3.780 3.763 3.745 3.728 3.709 3.696 3.678 3.660 3.643 3.626 3.608 0.929 0.912 0.894 0.000

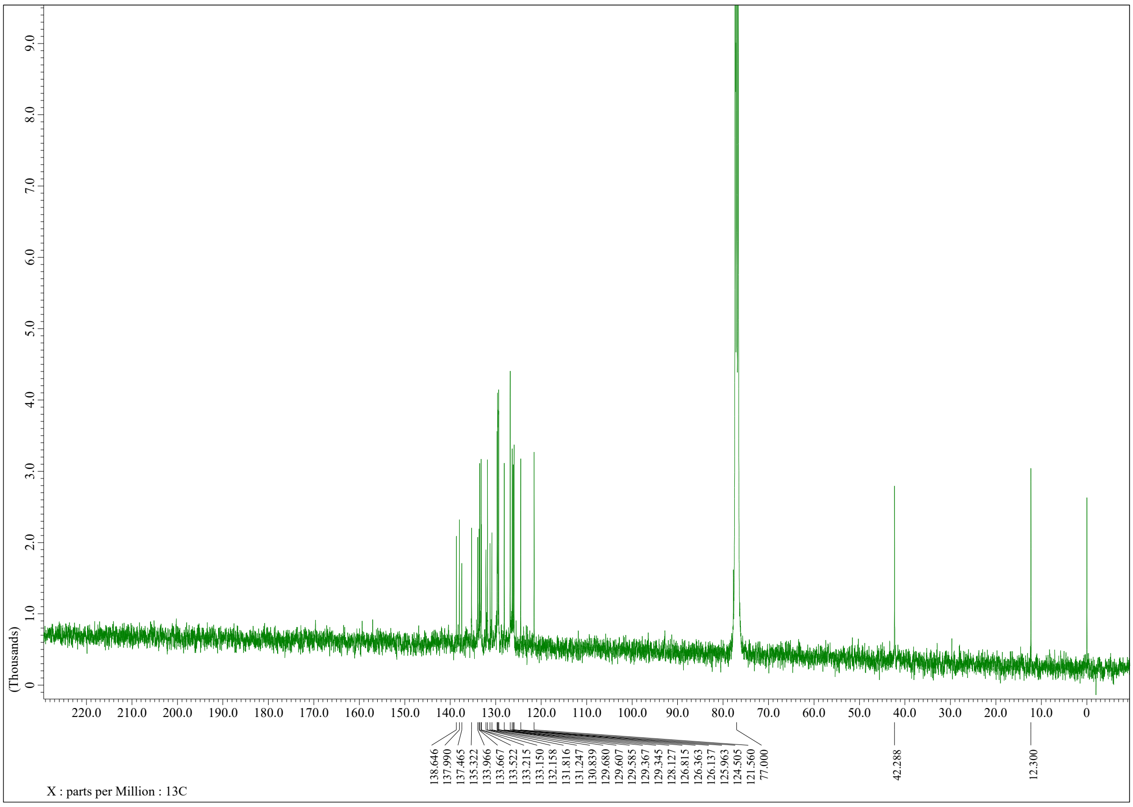
(Thousands)
X : parts per Million : 13C
138.646 137.990 137.465 135.322 133.966 133.667 133.522 133.215 133.150 132.158 131.816 131.247 130.839 129.680 129.607 129.585 129.367 129.345 128.127 126.815 126.363 126.137 125.963 124.505 121.560 77.000 42.288 12.300

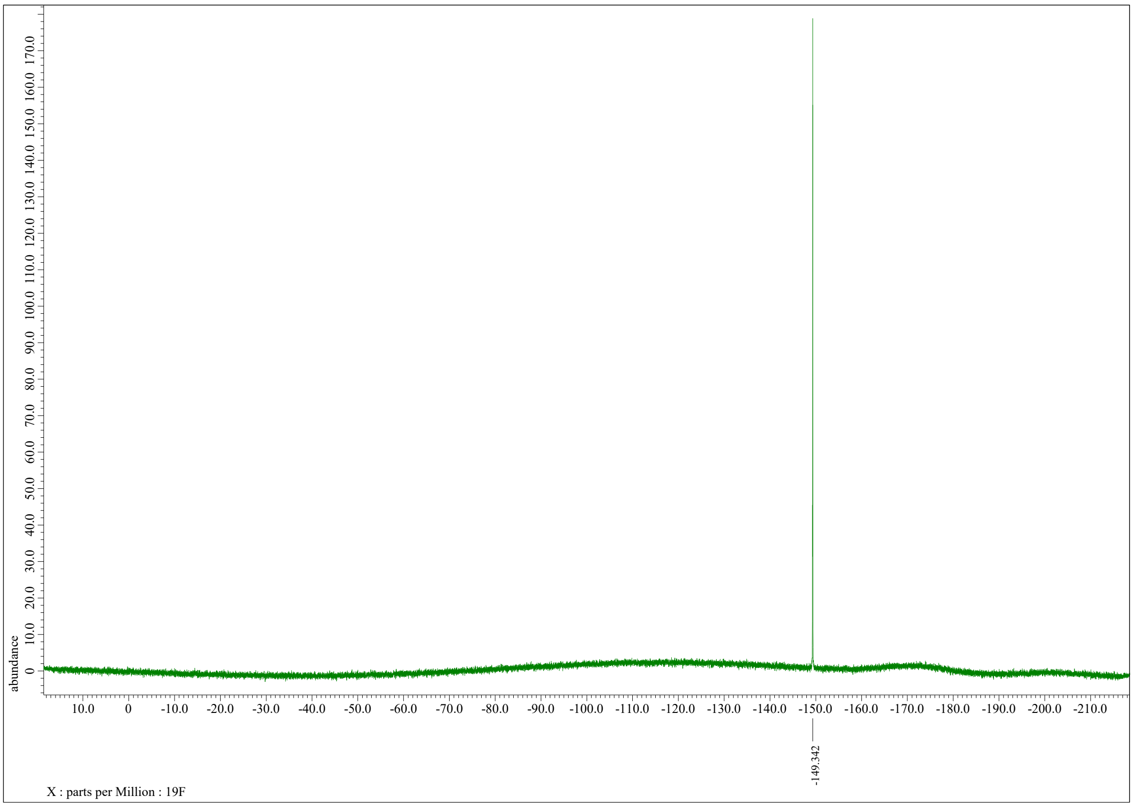

abundance
X : parts per Million : 19F
-149.342

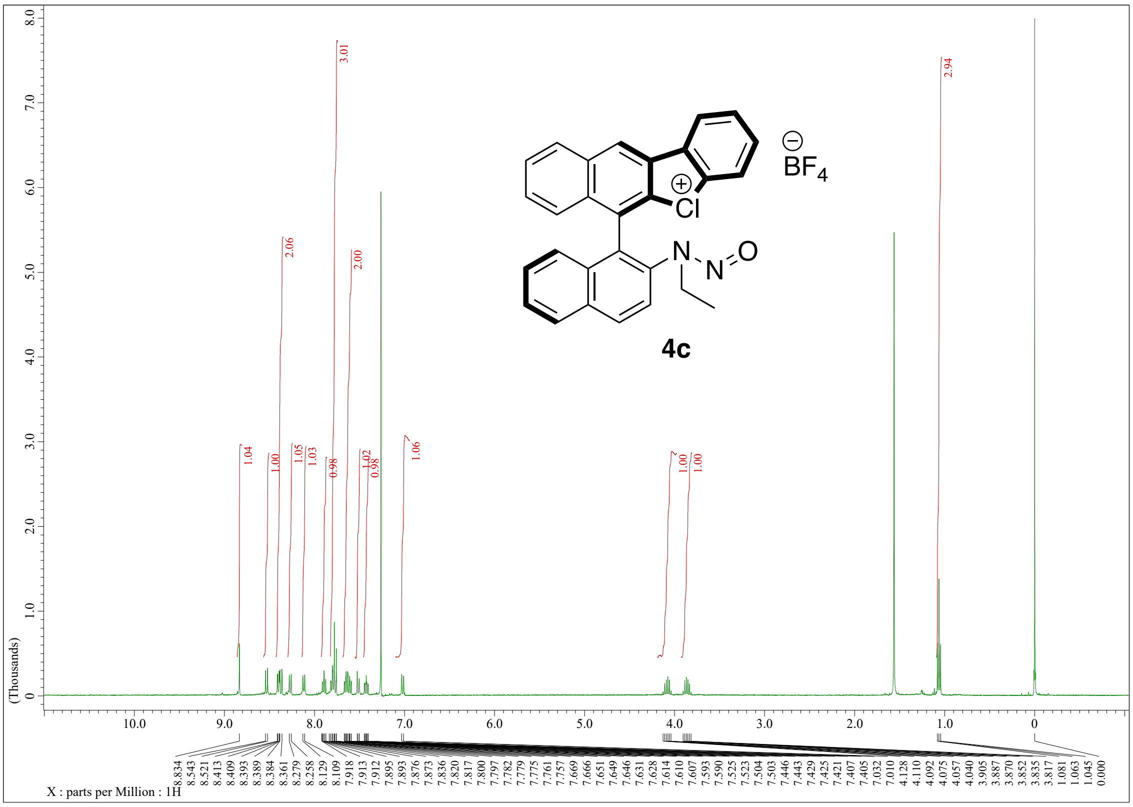
BF4
Cl
N
N
O
4c
(Thousands)
X : parts per Million : 1H
1.04
1.00
2.06
1.05
1.03
0.98
3.01
2.00
1.02
0.98
1.06
1.00
1.00
2.94
8.834 8.543 8.521 8.413 8.409 8.393 8.389 8.384 8.361 8.279 8.258 8.129 8.109 7.918 7.913 7.912 7.895 7.893 7.876 7.873 7.836 7.820 7.817 7.800 7.797 7.782 7.779 7.775 7.761 7.757 7.669 7.666 7.651 7.649 7.646 7.631 7.628 7.614 7.610 7.607 7.593 7.590 7.525 7.523 7.504 7.503 7.446 7.443 7.429 7.425 7.421 7.407 7.405 7.032 7.010 4.128 4.110 4.092 4.075 4.057 4.040 3.905 3.887 3.870 3.852 3.835 3.817 1.081 1.063 1.045 0.000

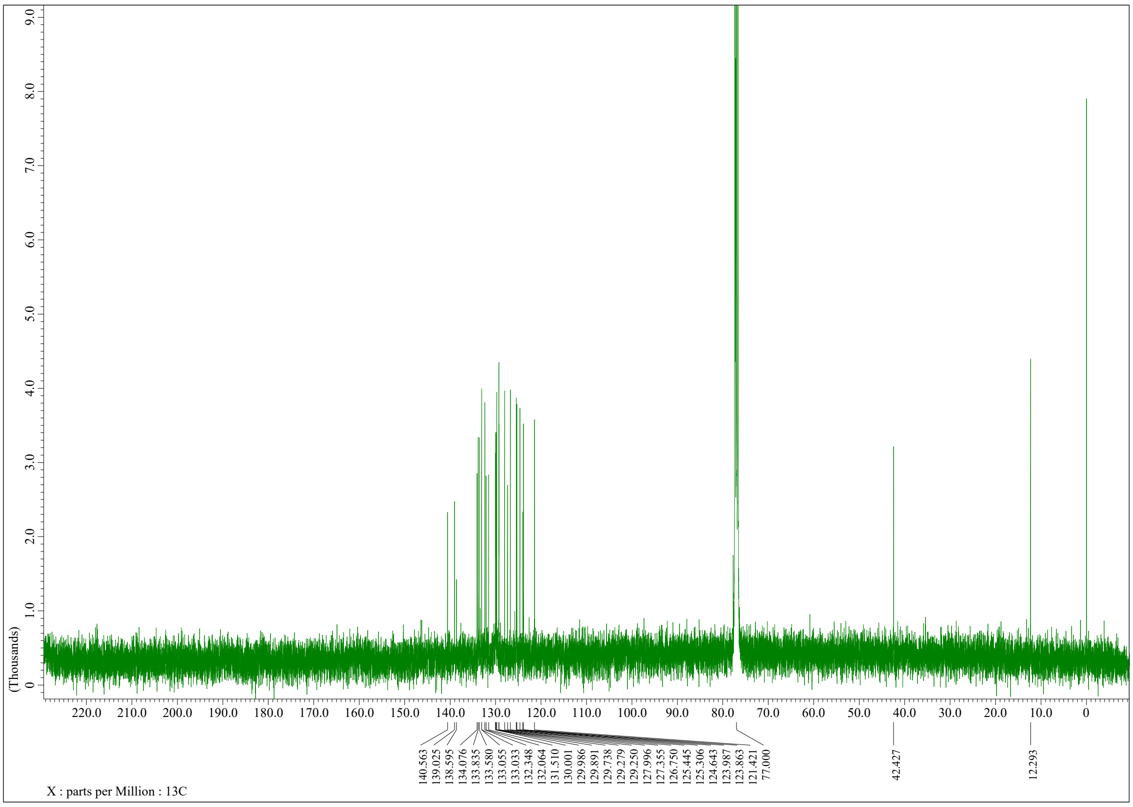
(Thousands)
X : parts per Million : 13C
140.563 139.025 138.595 134.076 133.835 133.580 133.055 133.033 132.348 132.064 131.510 130.001 129.986 129.891 129.738 129.279 129.250 127.996 127.355 126.750 125.445 125.306 124.643 123.987 123.863 121.421 77.000
42.427
12.293

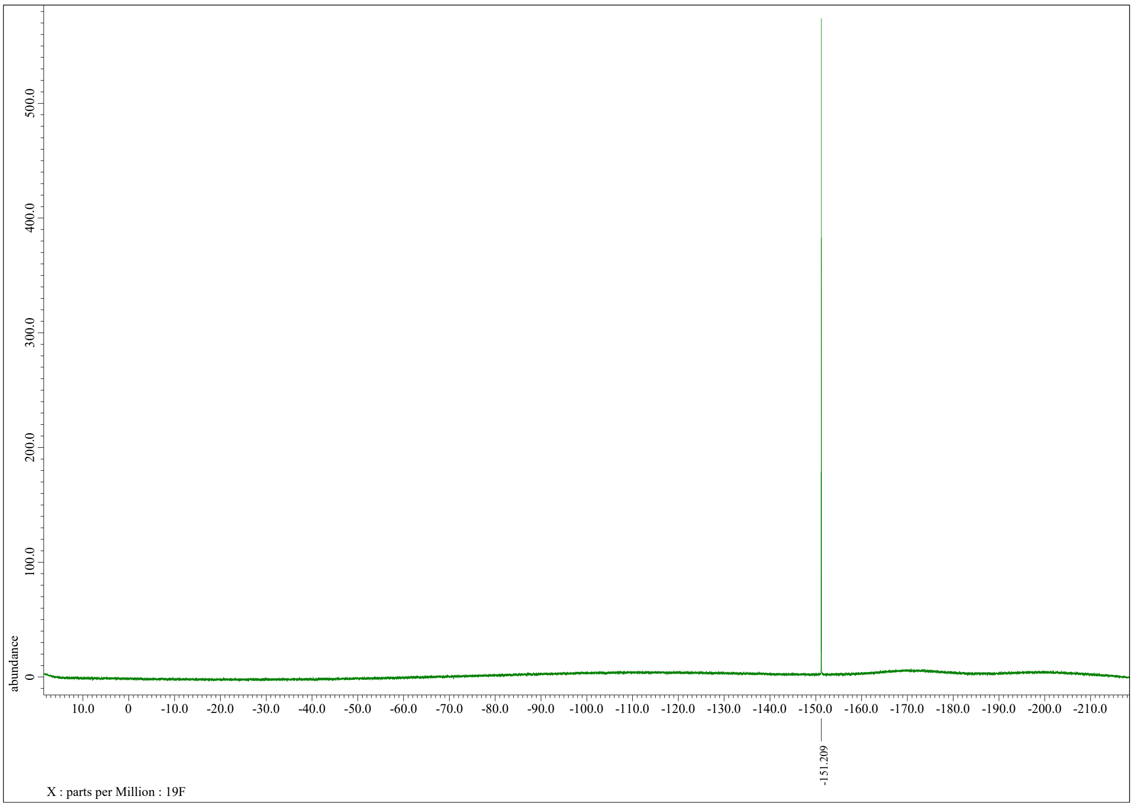

abundance
500.0
400.0
300.0
200.0
100.0
0
10.0
0
-10.0
-20.0
-30.0
-40.0
-50.0
-60.0
-70.0
-80.0
-90.0
-100.0
-110.0
-120.0
-130.0
-140.0
-150.0
-160.0
-170.0
-180.0
-190.0
-200.0
-210.0
-151.209
X : parts per Million : 19F

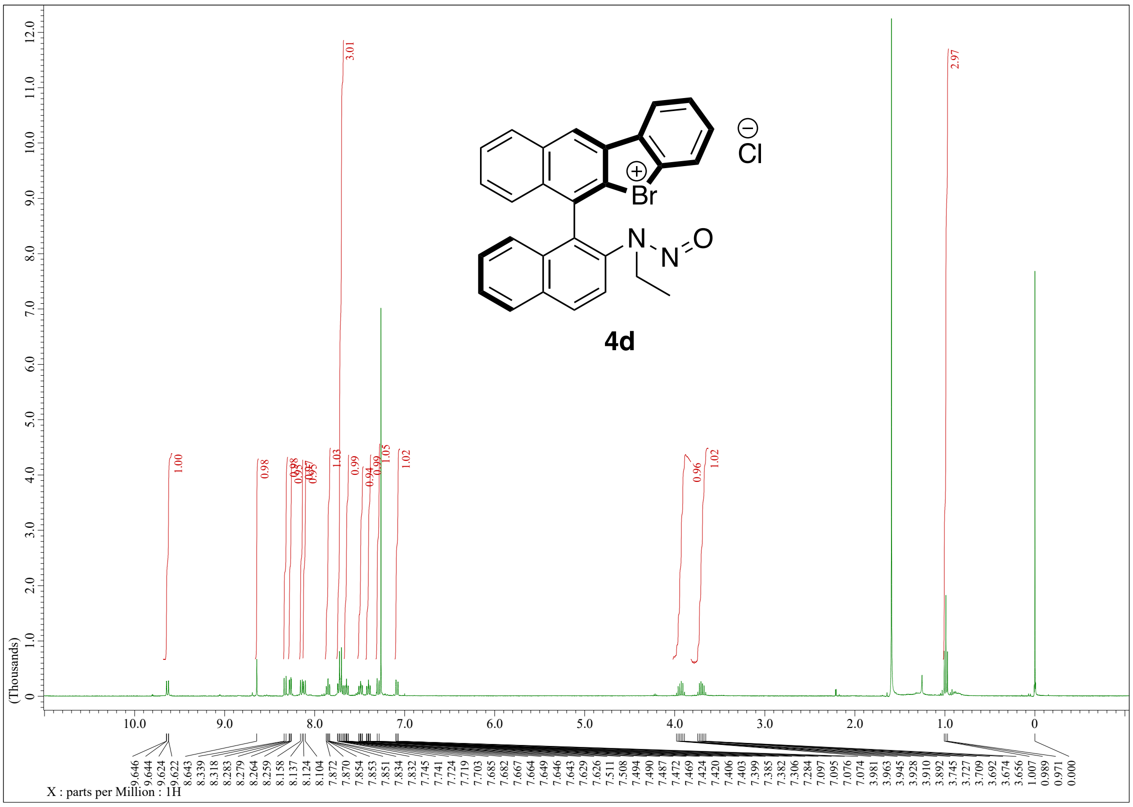

Br
Cl
N
N
O
4d
(Thousands)
X : parts per Million : 1H

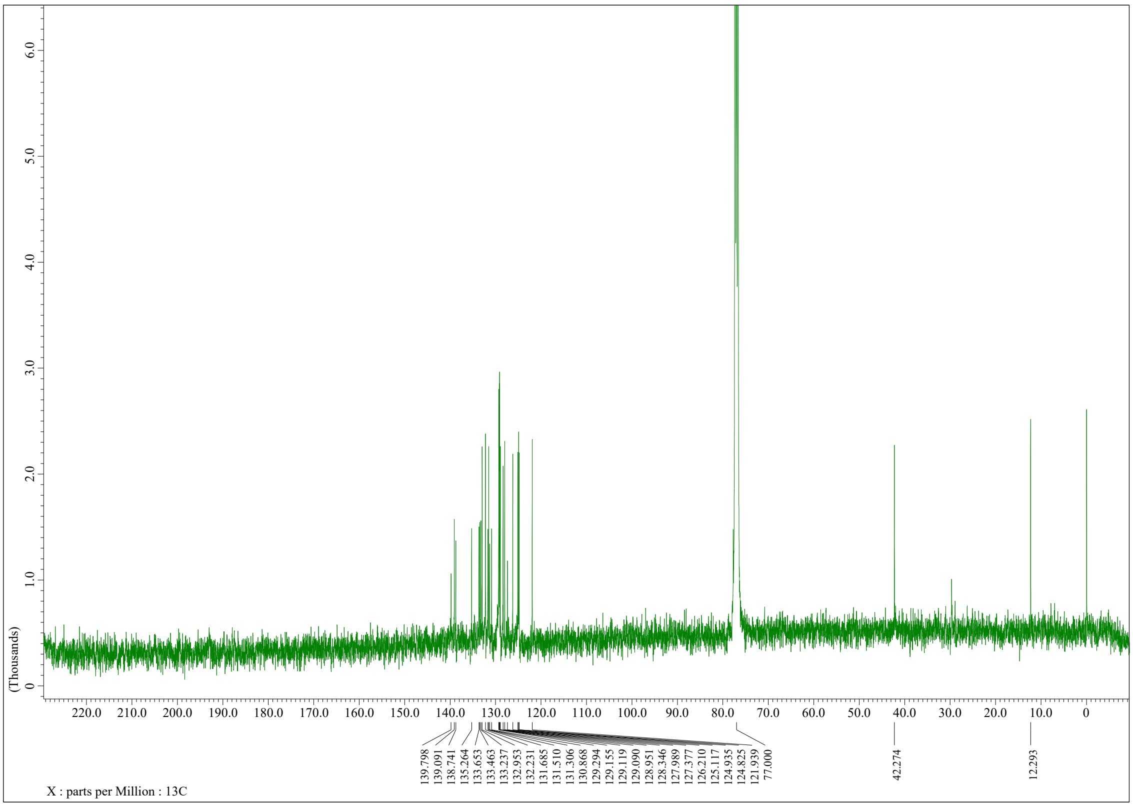

(Thousands)
X : parts per Million : 13C

・ HPLC charts of **7a** for Scheme 2.

Racemic sample of **7a**

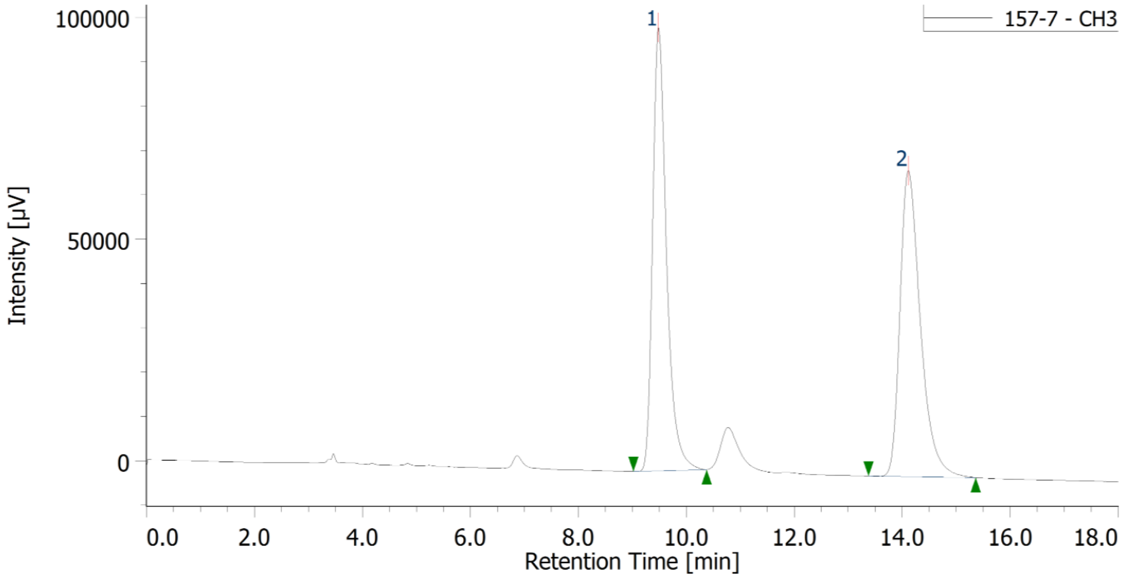

#	ピーク名	CH	tR [min]	Area [μV·sec]	Height [μV]	Area%	Height%
1	Unknown	3	9.48	1775891	99921	49.539	59.170
2	Unknown	3	14.11	1808946	68951	50.461	40.830

Catalyzed by **4a**

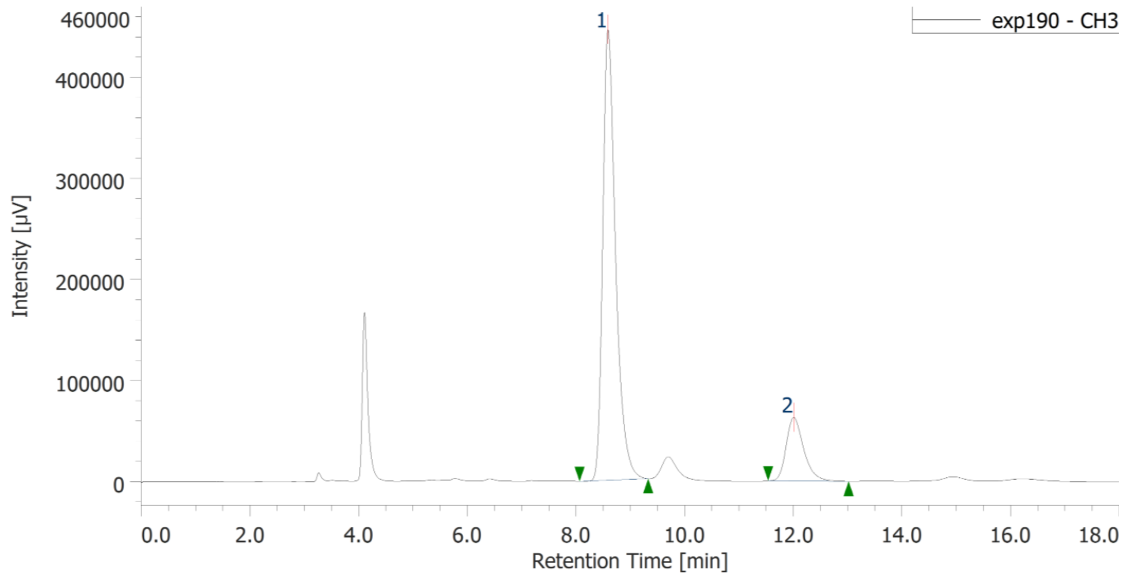

#	ピーク名	CH	tR [min]	Area [μV·sec]	Height [μV]	Area%	Height%
1	Unknown	3	8.59	7331298	445910	83.891	87.648
2	Unknown	3	12.01	1407809	62840	16.109	12.352

Catalyzed by **4b**

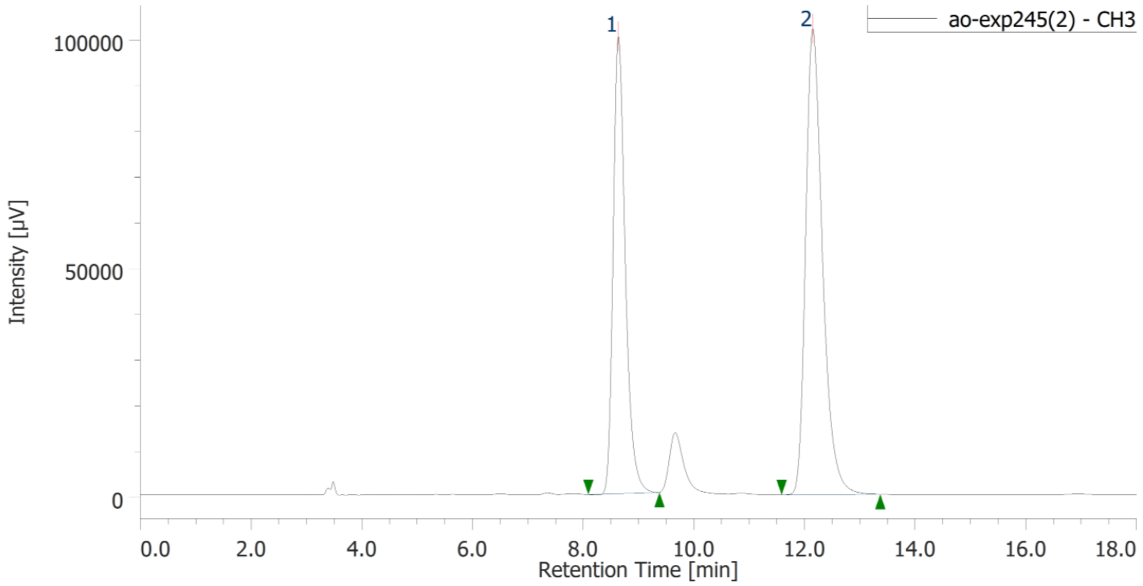

#	ピーク名	CH	tR [min]	面積 [μV·sec]	高さ [μV]	面積%	高さ%
1	Unknown	3	8.633	1522932	99893	41.4	49.5
2	Unknown	3	12.150	2157293	101828	58.6	50.5

Catalyzed by **4c**

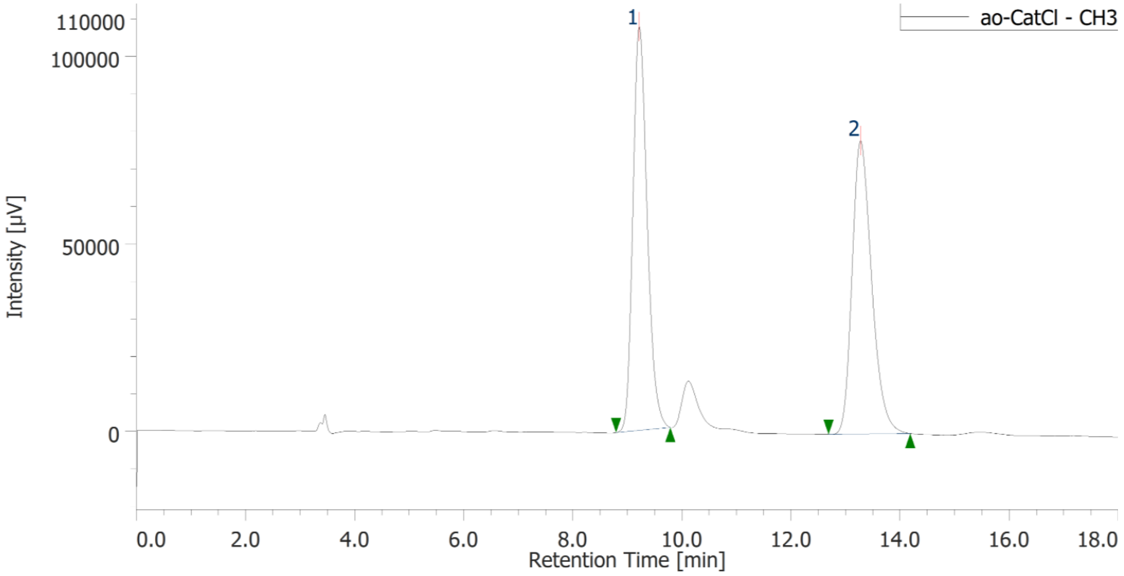

#	ピーク名	CH	tR [min]	面積 [μV·sec]	高さ [μV]	面積%	高さ%
1	Unknown	3	9.217	1951692	107727	49.9	57.9
2	Unknown	3	13.275	1957723	78277	50.1	42.1

Catalyzed by **4d**

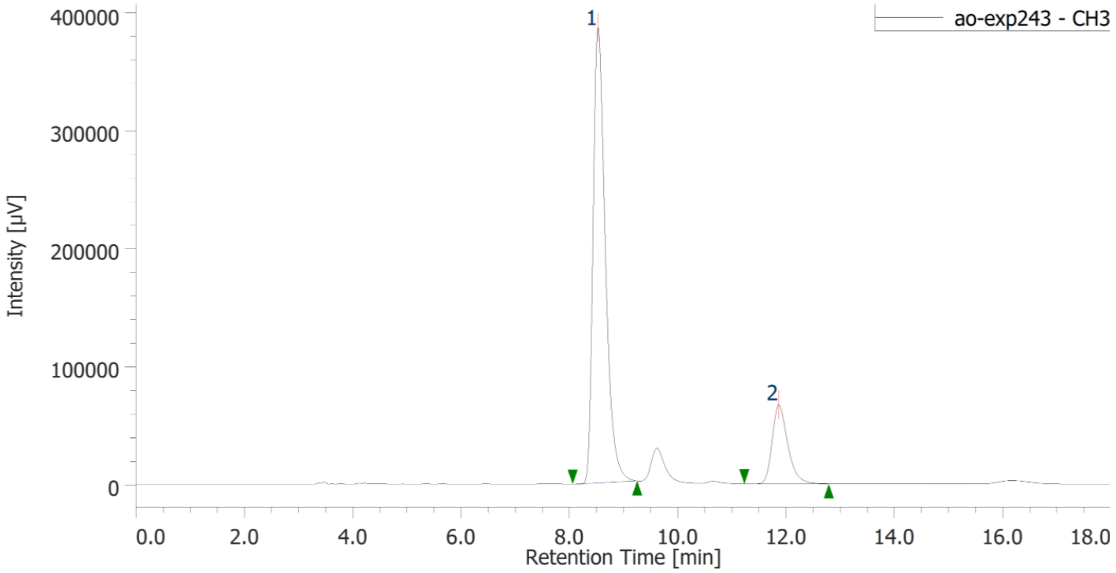

#	ピーク名	CH	tR [min]	面積 [μV·sec]	高さ [μV]	面積%	高さ%
1	Unknown	3	8.525	6083829	385607	82.1	85.2
2	Unknown	3	11.867	1330858	66772	17.9	14.8

・ HPLC charts of **7a** for Table 1.

Entry 1 (toluene)

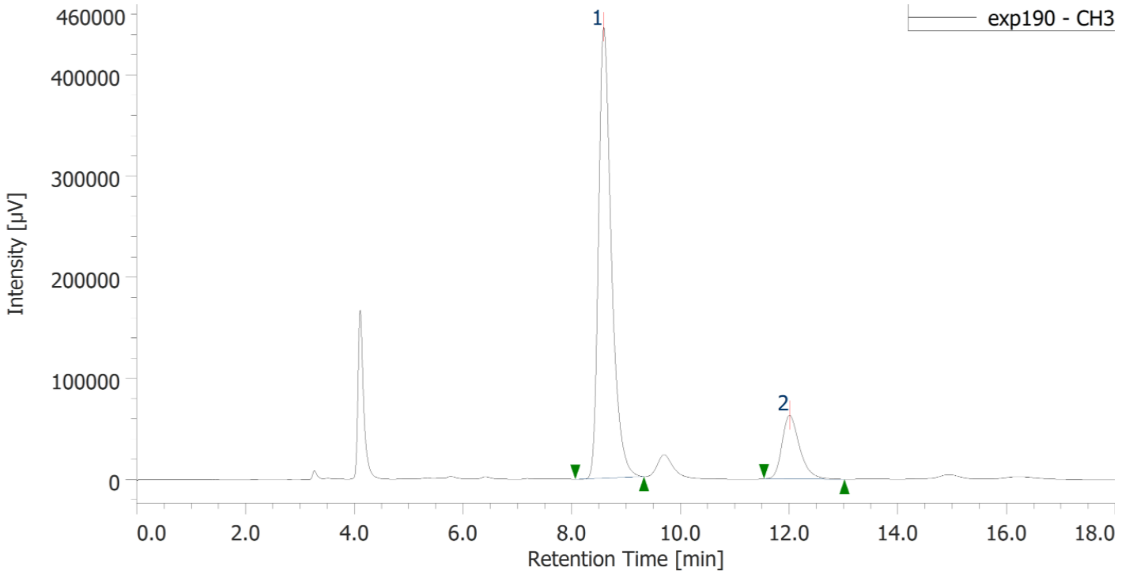

#	ピーク名	CH	tR [min]	Area [μV·sec]	Height [μV]	Area%	Height%
1	Unknown	3	8.59	7331298	445910	83.891	87.648
2	Unknown	3	12.01	1407809	62840	16.109	12.352

Entry 2 (THF)

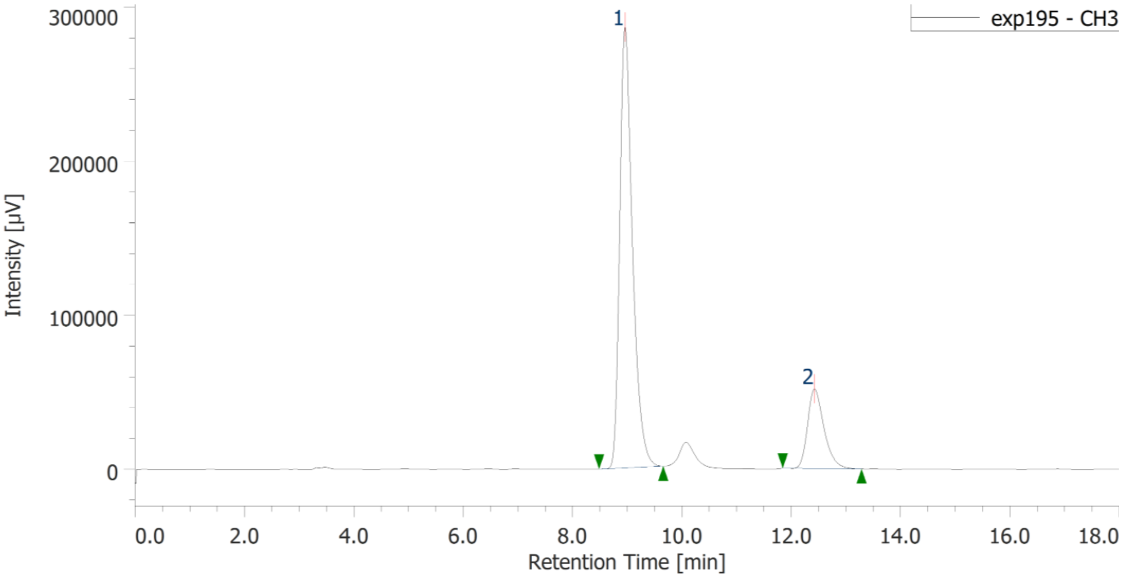

#	ピーク名	CH	tR [min]	Area [μV·sec]	Height [μV]	Area%	Height%
1	Unknown	3	8.96	4651974	286067	81.190	84.701
2	Unknown	3	12.43	1077770	51669	18.810	15.299

Entry 3 (CH_2Cl_2)

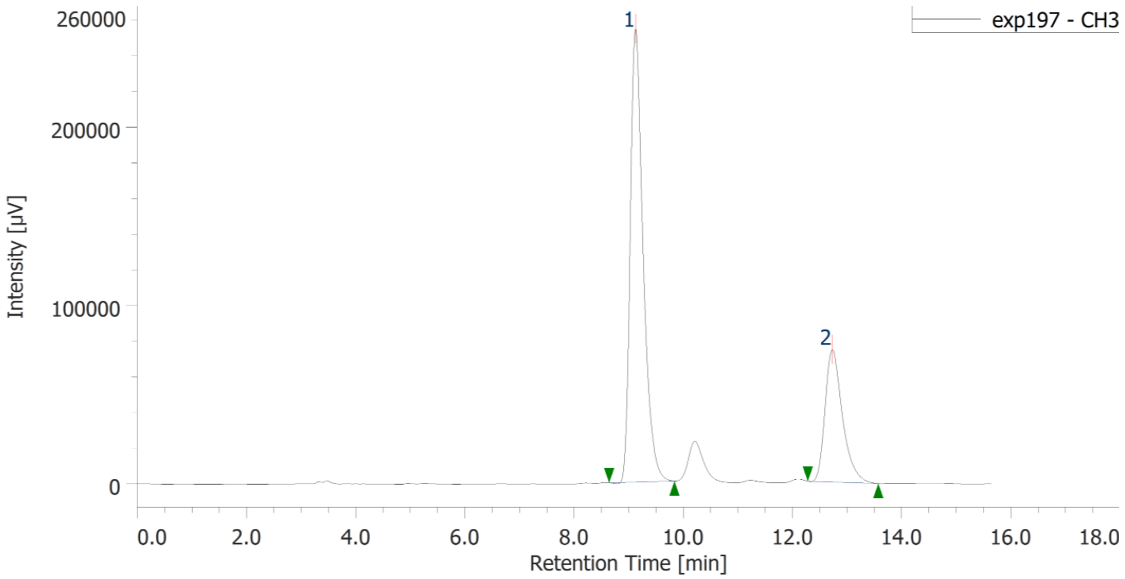

#	ピーク名	CH	tR [min]	Area [µV·sec]	Height [µV]	Area%	Height%
1	Unknown	3	9.13	4156467	254127	71.672	77.397
2	Unknown	3	12.72	1642800	74217	28.328	22.603

Entry 4 (Et_2O)

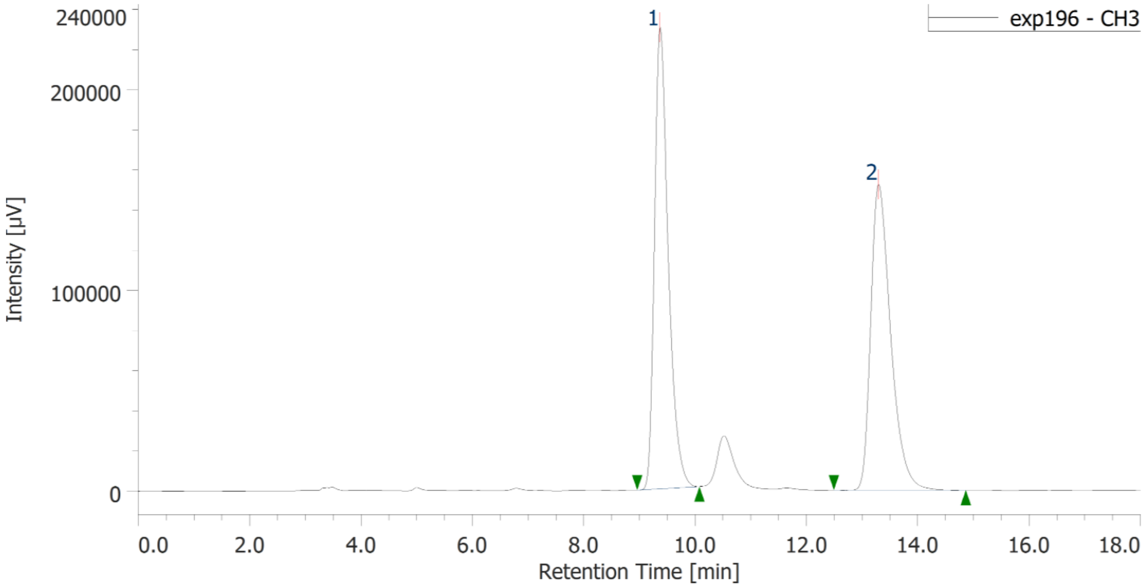

#	ピーク名	CH	tR [min]	Area [µV·sec]	Height [µV]	Area%	Height%
1	Unknown	3	9.37	3953222	229910	51.082	60.151
2	Unknown	3	13.29	3785806	152310	48.918	39.849

Entry 5 (CH_3CN)

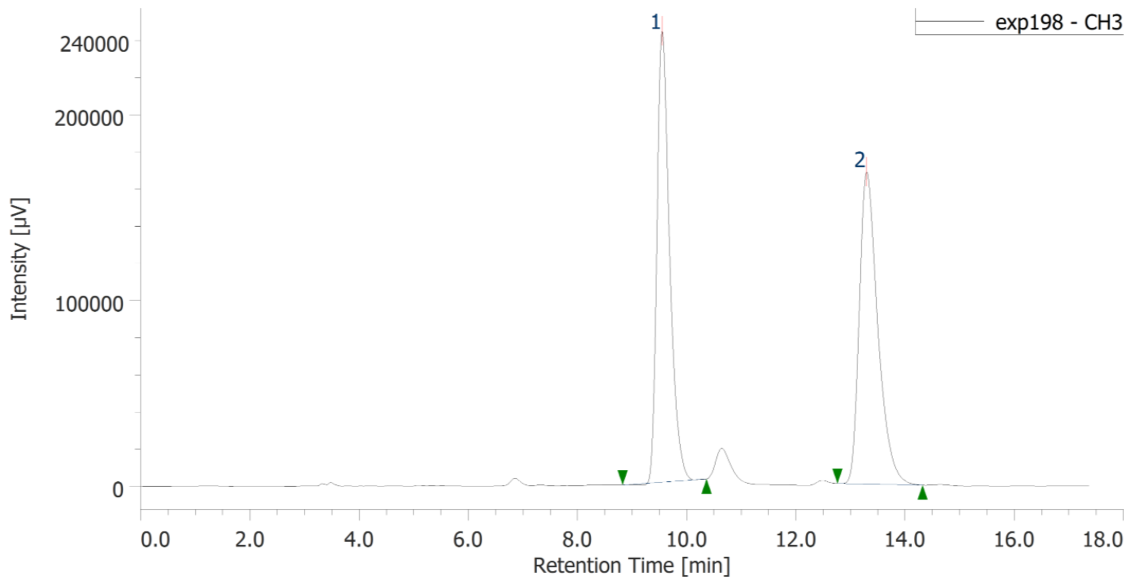

#	ピーク名	CH	tR [min]	Area [µV·sec]	Height [µV]	Area%	Height%
1	Unknown	3	9.55	3911439	242861	49.715	59.100
2	Unknown	3	13.29	3956320	168069	50.285	40.900

Entry 6 (-10 °C)

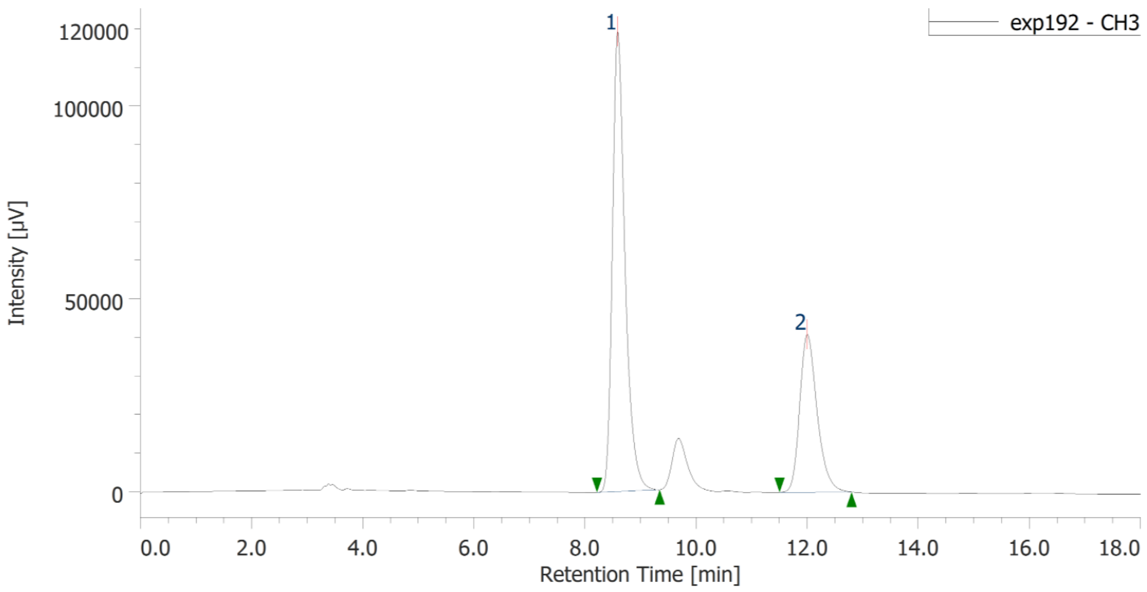

#	ピーク名	CH	tR [min]	Area [µV·sec]	Height [µV]	Area%	Height%
1	Unknown	3	8.59	1891690	119256	67.951	74.441
2	Unknown	3	12.00	892200	40946	32.049	25.559

Entry 7 (-30 °C)

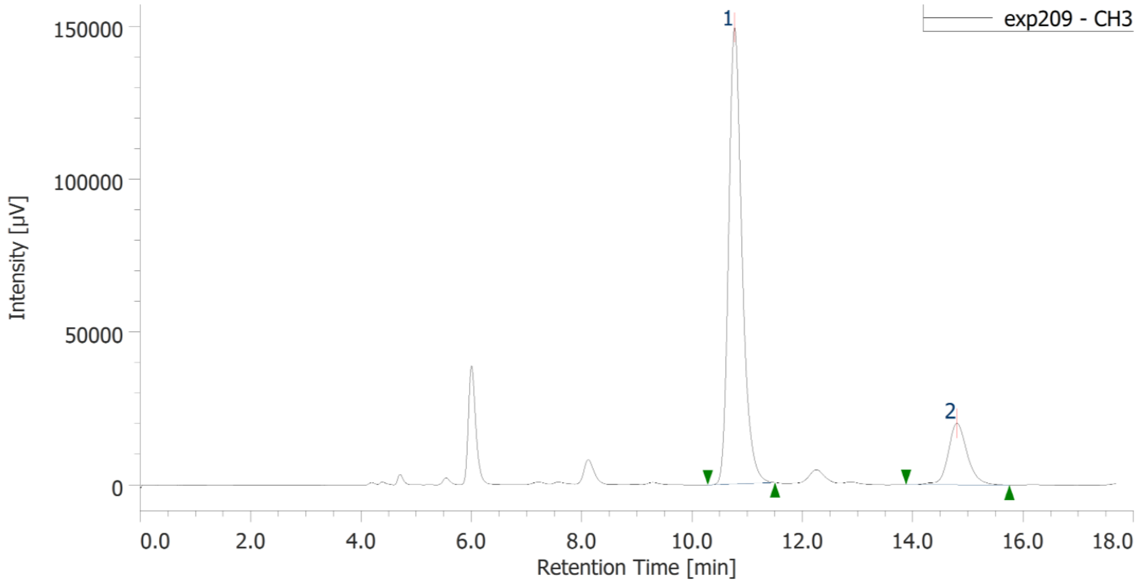

#	ピーク名	CH	tR [min]	Area [μV·sec]	Height [μV]	Area%	Height%
1	Unknown	3	10.77	2518823	149219	84.010	88.122
2	Unknown	3	14.80	479416	20113	15.990	11.878

Entry 8 (catalyzed by 1 mol% of **4a**)

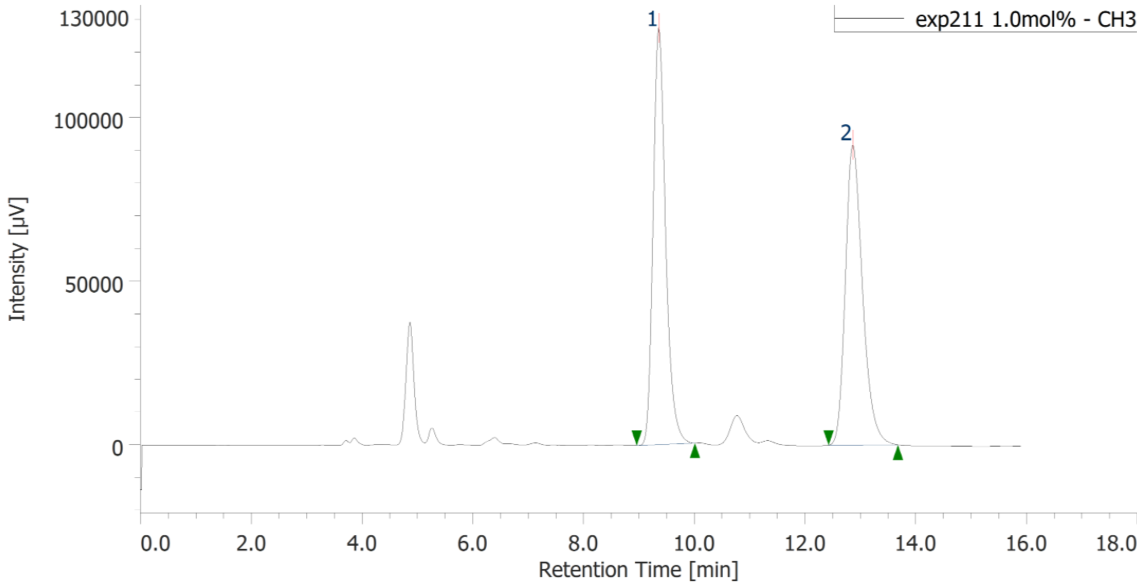

#	ピーク名	CH	tR [min]	Area [μV·sec]	Height [μV]	Area%	Height%
1	Unknown	3	9.36	1995053	127209	50.220	58.117
2	Unknown	3	12.86	1977560	91677	49.780	41.883

Entry 9 (catalyzed by 5 mol% of **4a**)

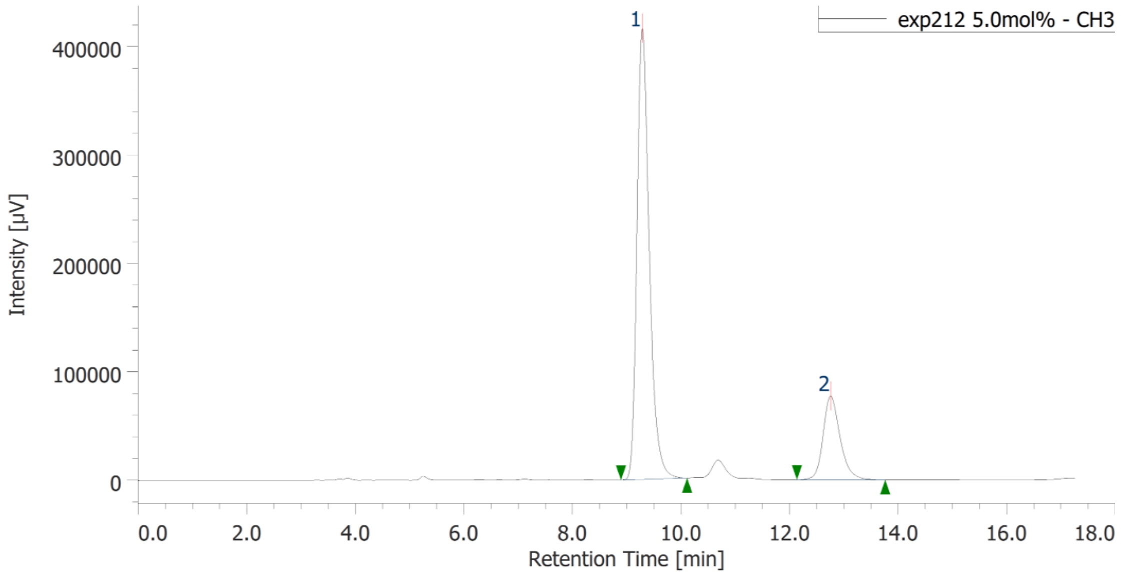

#	ピーク名	CH	tR [min]	Area [µV·sec]	Height [µV]	Area%	Height%
1	Unknown	3	9.28	6530331	415943	79.512	84.328
2	Unknown	3	12.75	1682690	77299	20.488	15.672

Entry 10 (no catalyst)

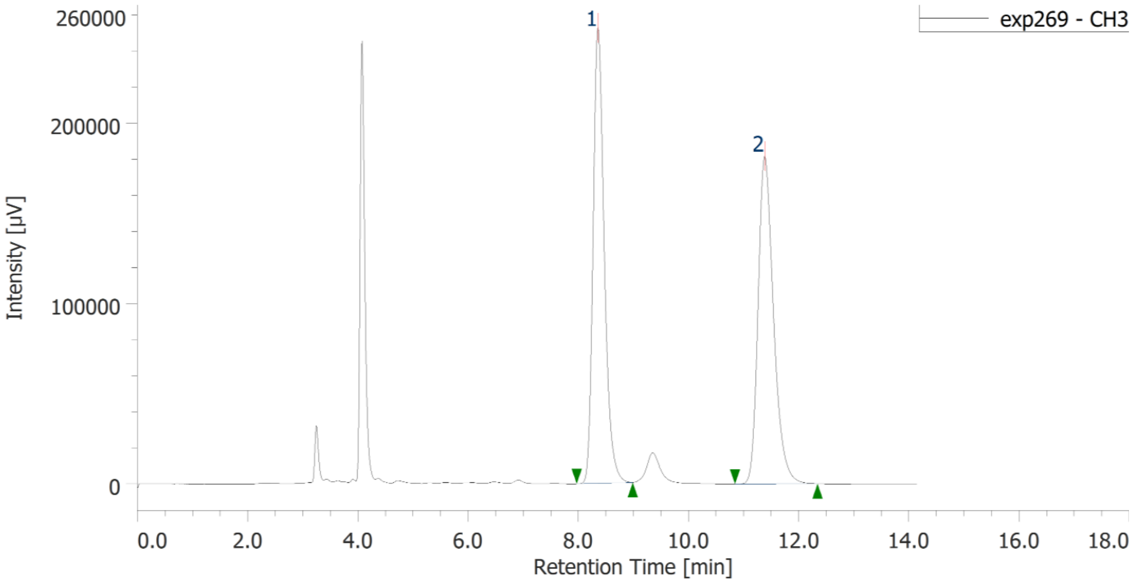

#	ピーク名	CH	tR [min]	Area [µV·sec]	Height [µV]	Area%	Height%
1	Unknown	3	8.36	3540468	252506	49.938	58.152
2	Unknown	3	11.38	3549256	181714	50.062	41.848

・ Charts for Scheme 3

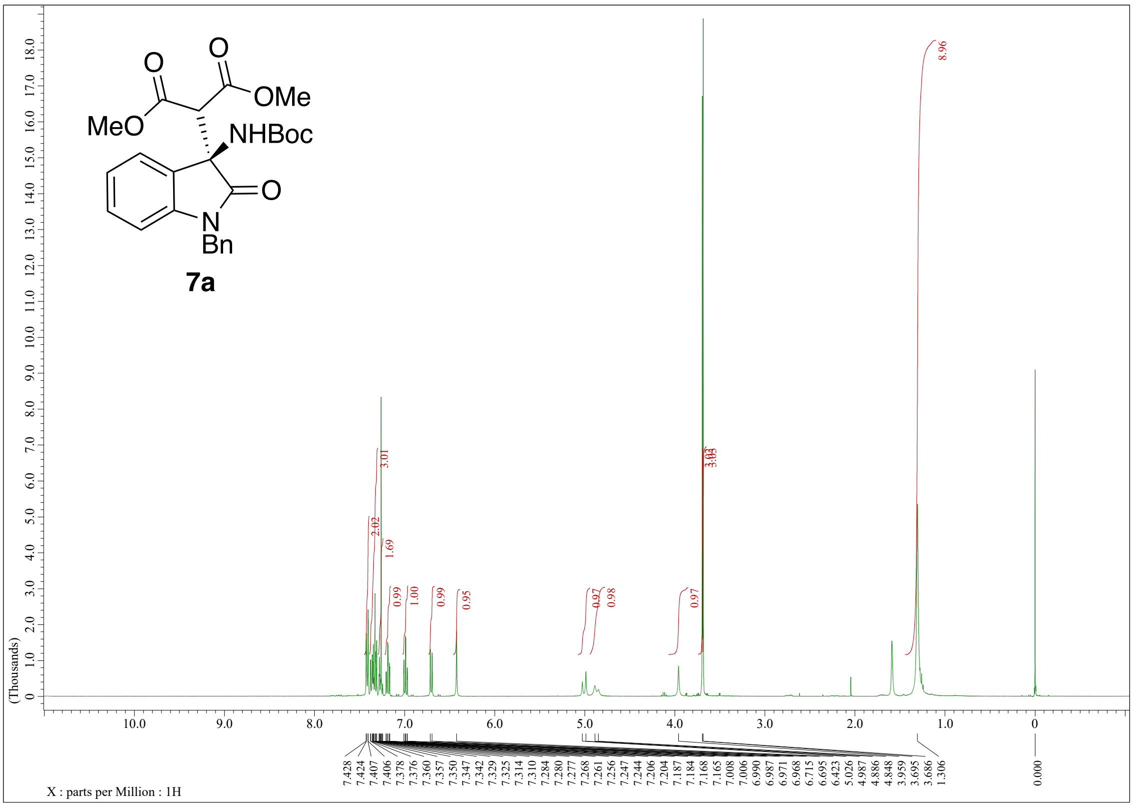

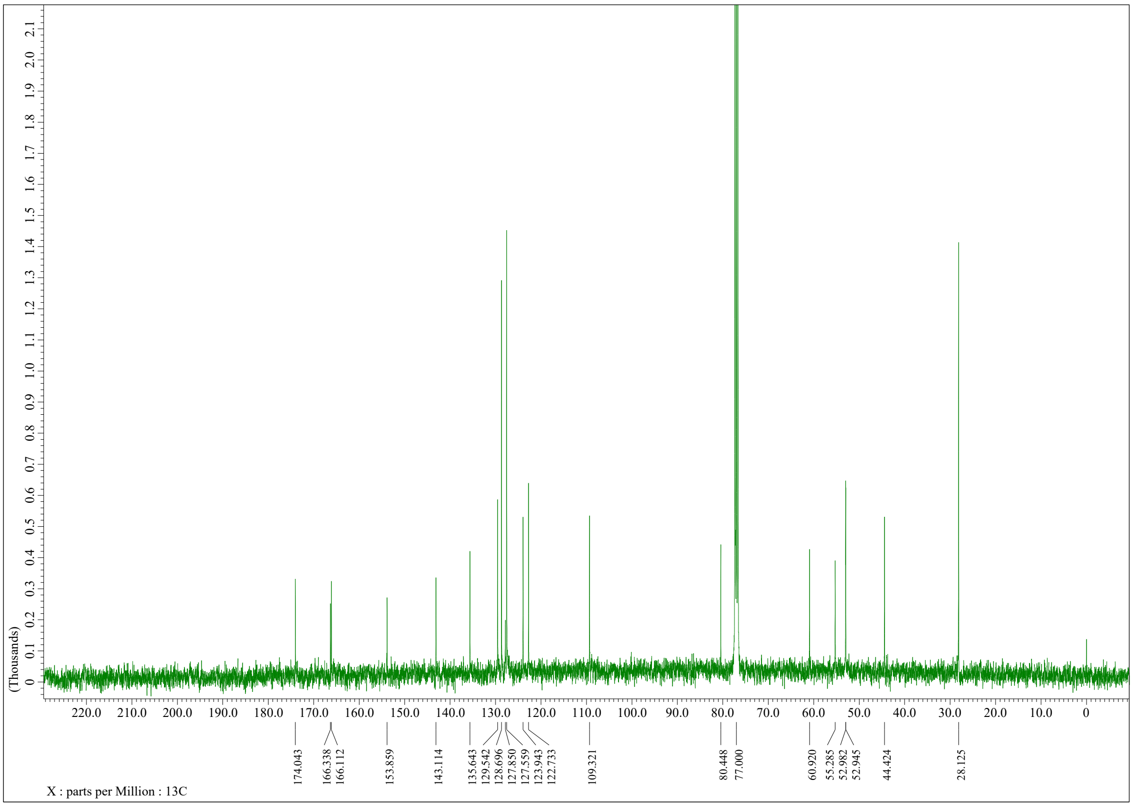

Racemic sample of **7a**

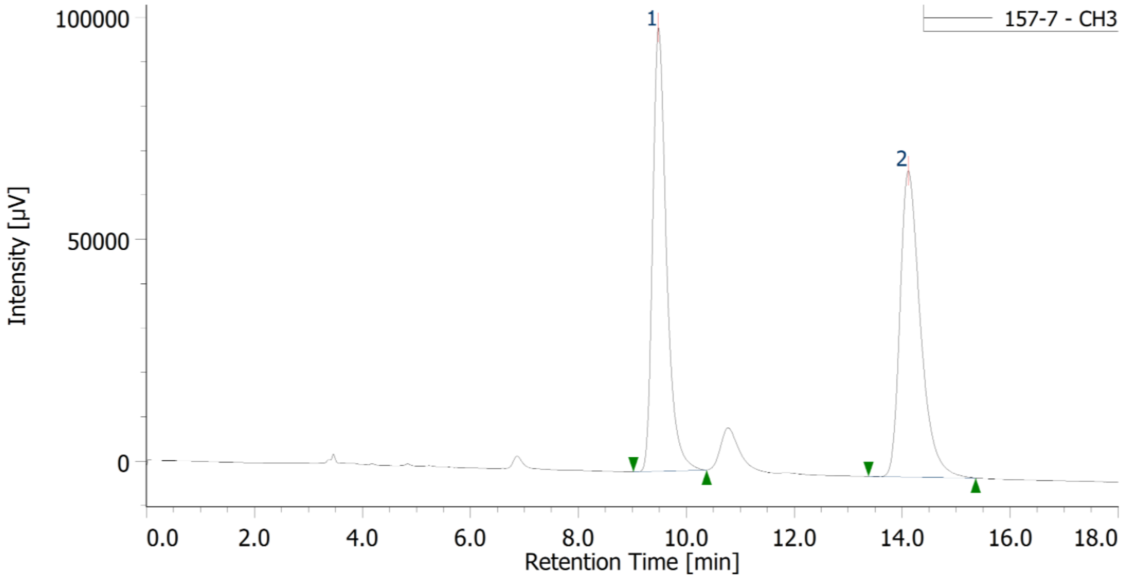

#	ピーク名	CH	tR [min]	Area [μV·sec]	Height [μV]	Area%	Height%
1	Unknown	3	9.48	1775891	99921	49.539	59.170
2	Unknown	3	14.11	1808946	68951	50.461	40.830

7a catalyzed by **4a**

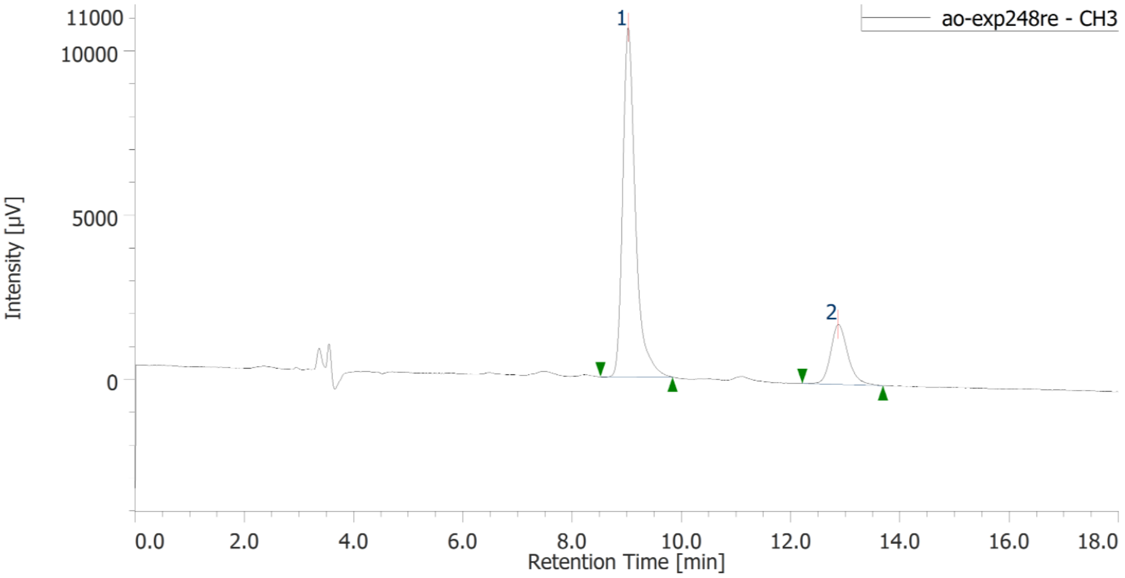

#	ピーク名	CH	tR [min]	面積 [μV·sec]	高さ [μV]	面積%	高さ%
1	Unknown	3	9.025	172319	10641	81.1	85.3
2	Unknown	3	12.867	40037	1829	18.9	14.7

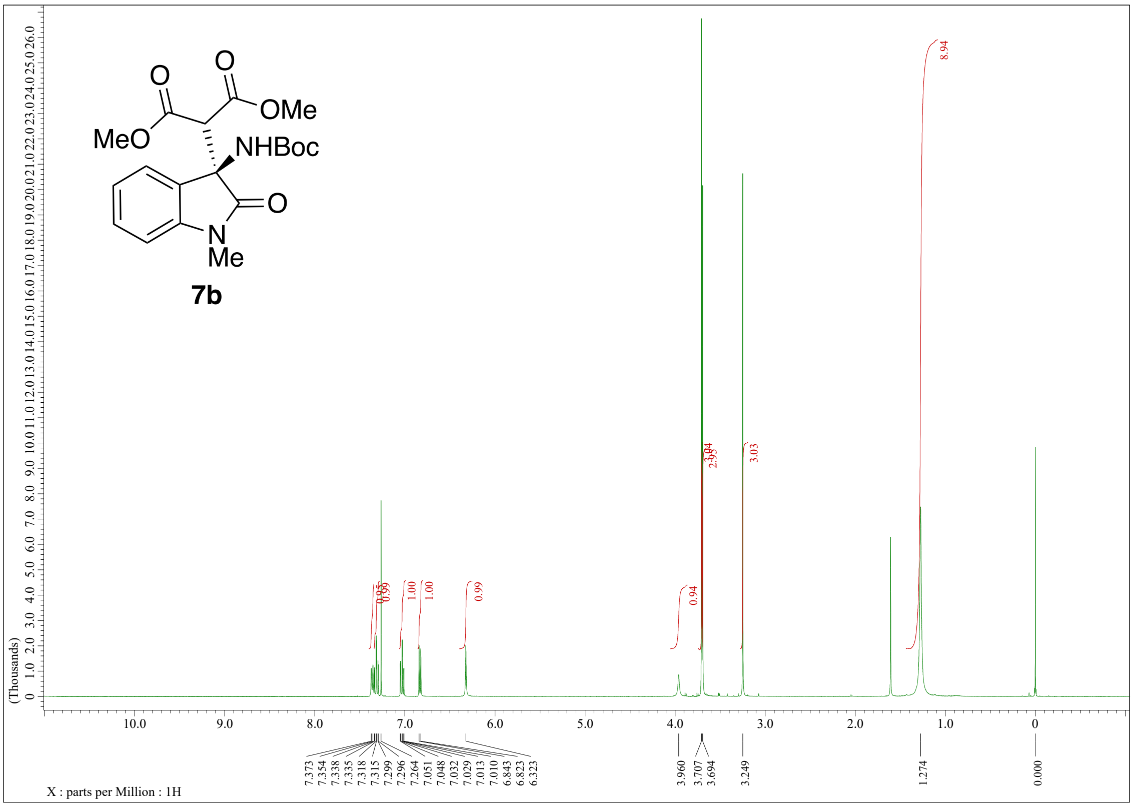

7b
NHBoc
MeO
OMe
X : parts per Million : 1H
(Thousands)

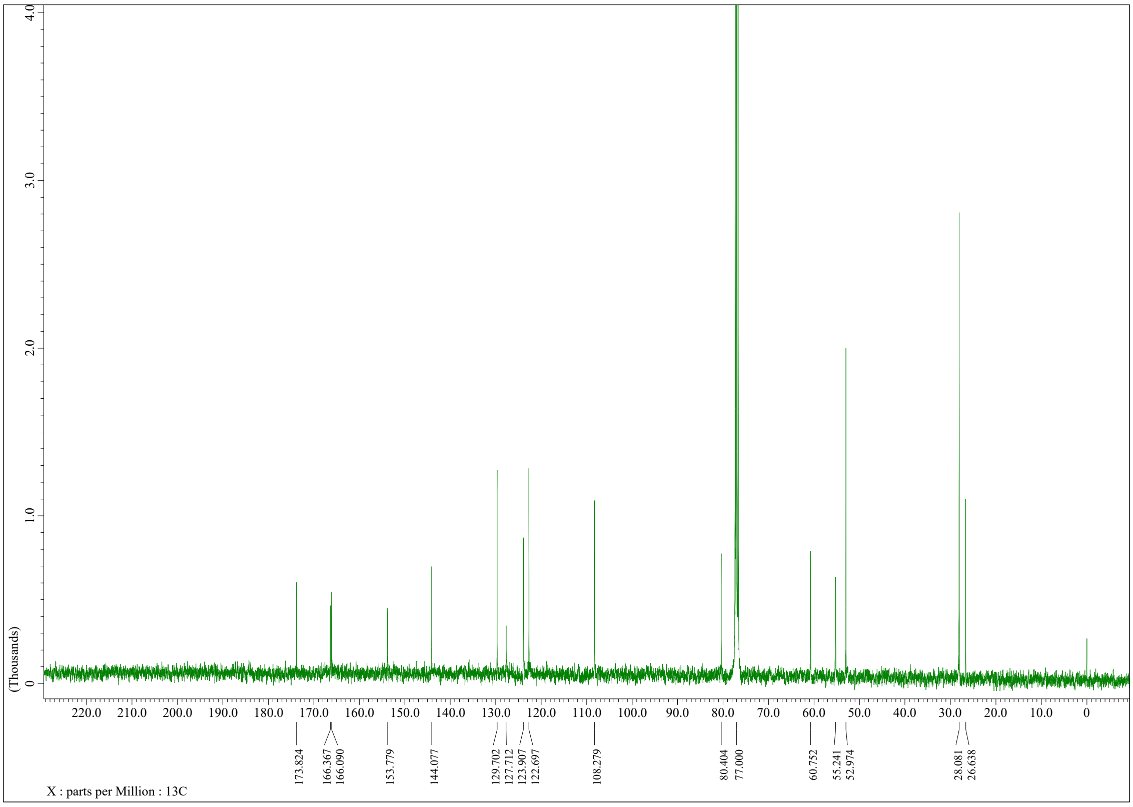

X : parts per Million : 13C
(Thousands)

Racemic sample of **7b**

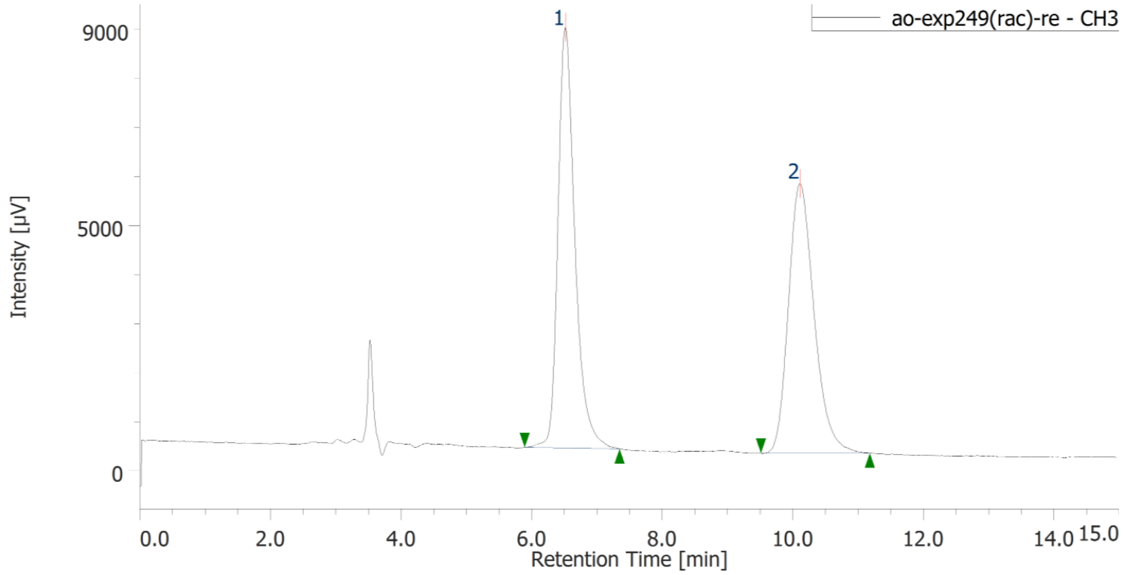

#	ピーク名	CH	tR [min]	面積 [µV·sec]	高さ [µV]	面積%	高さ%
1	Unknown	3	6.517	154628	8579	50.8	60.9
2	Unknown	3	10.108	150023	5500	49.2	39.1

7b catalyzed by **4a**

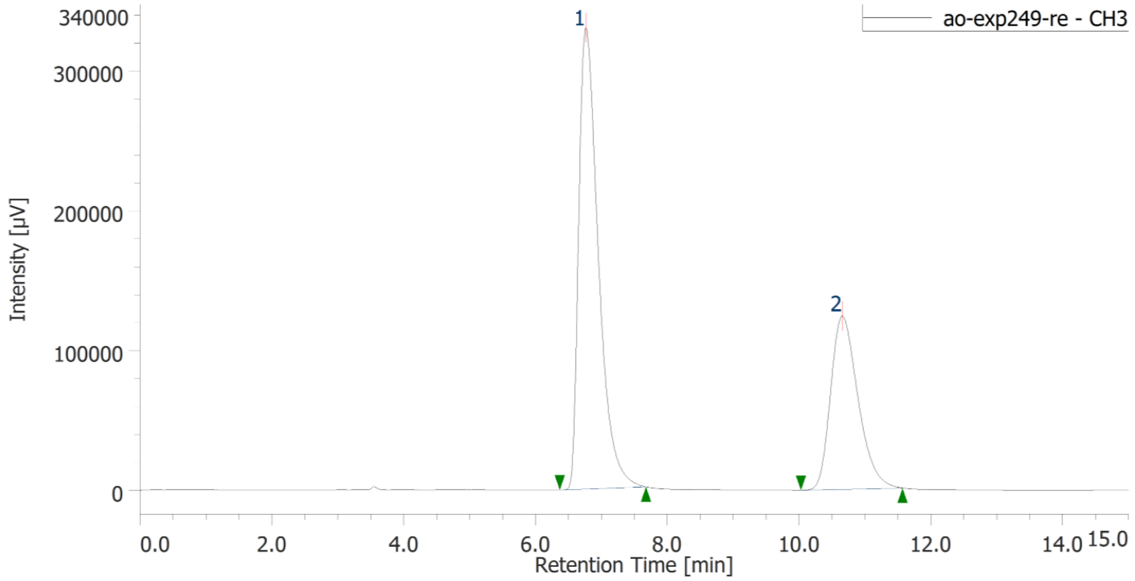

#	ピーク名	CH	tR [min]	面積 [µV·sec]	高さ [µV]	面積%	高さ%
1	Unknown	3	6.767	6798246	329619	65.6	72.7
2	Unknown	3	10.658	3564843	123669	34.4	27.3

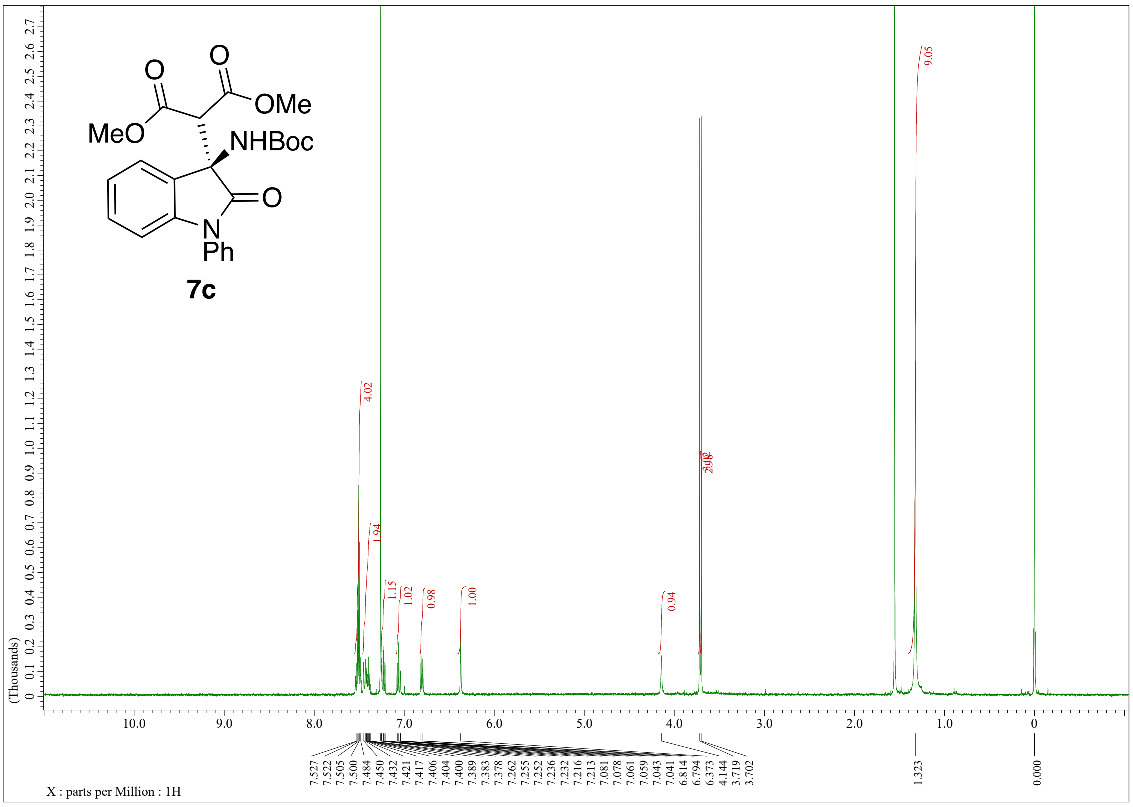
MeO
O
O
OMe
NHBoc
O
N
Ph
7c
4.02
1.94
1.15
1.02
0.98
1.00
0.94
9.05
(Thousands)
7.527 7.522 7.505 7.500 7.484 7.450 7.432 7.421 7.417 7.406 7.404 7.400 7.389 7.383 7.378 7.262 7.255 7.252 7.236 7.232 7.216 7.213 7.081 7.078 7.061 7.059 7.043 7.041 6.814 6.794 6.373 4.144 3.719 3.702
1.323
0.000
X : parts per Million : 1H

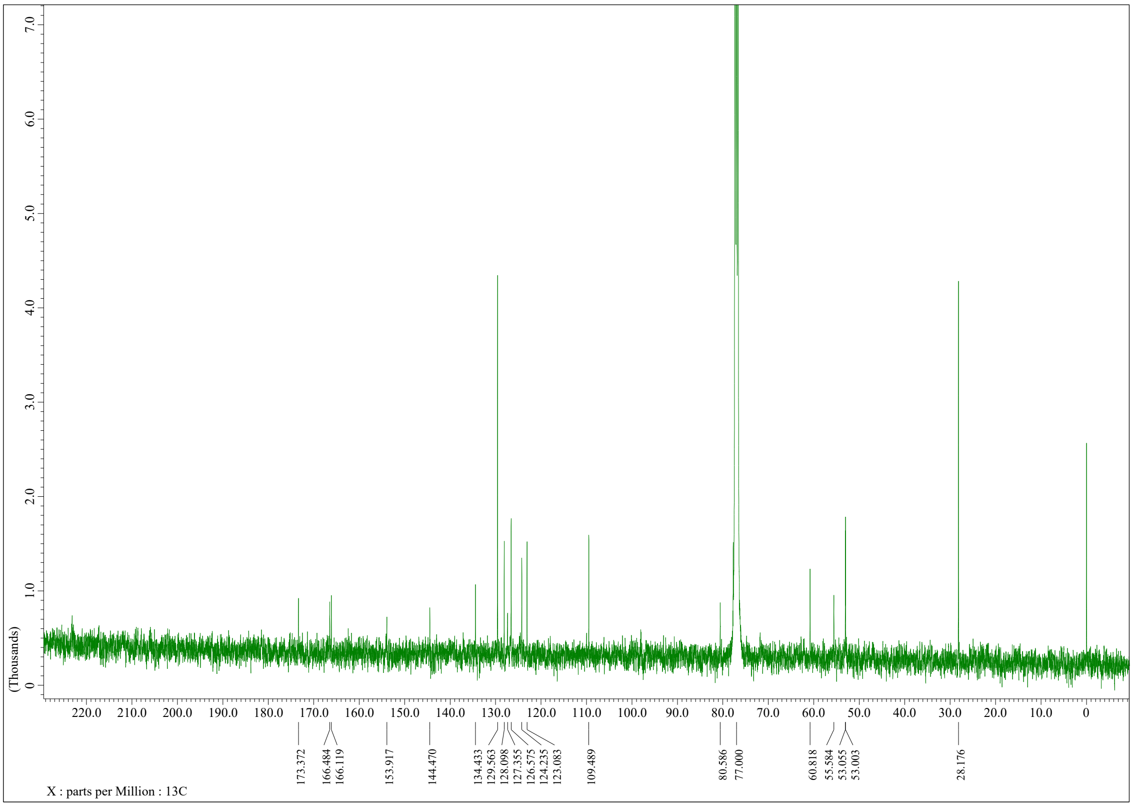
(Thousands)
173.372
166.484
166.119
153.917
144.470
134.433
129.563
128.098
127.355
126.575
124.235
123.083
109.489
80.586
77.000
60.818
55.584
53.055
53.003
28.176
X : parts per Million : 13C

Racemic sample of **7c**

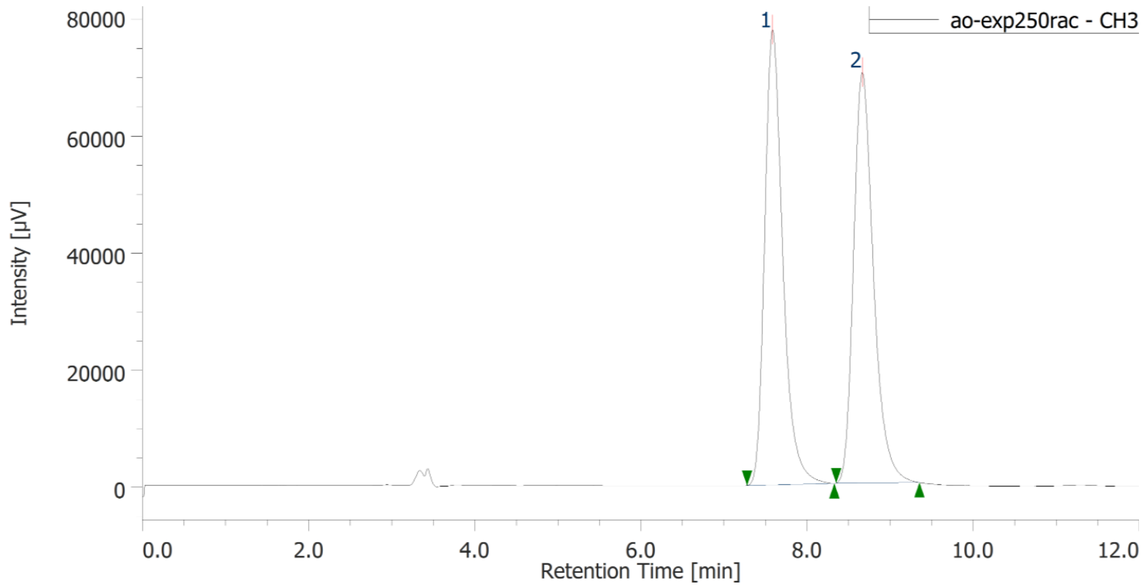

#	ピーク名	CH	tR [min]	面積 [µV·sec]	高さ [µV]	面積%	高さ%
1	Unknown	3	7.583	1178795	77792	50.3	52.6
2	Unknown	3	8.667	1166613	70149	49.7	47.4

7c catalyzed by **4a**

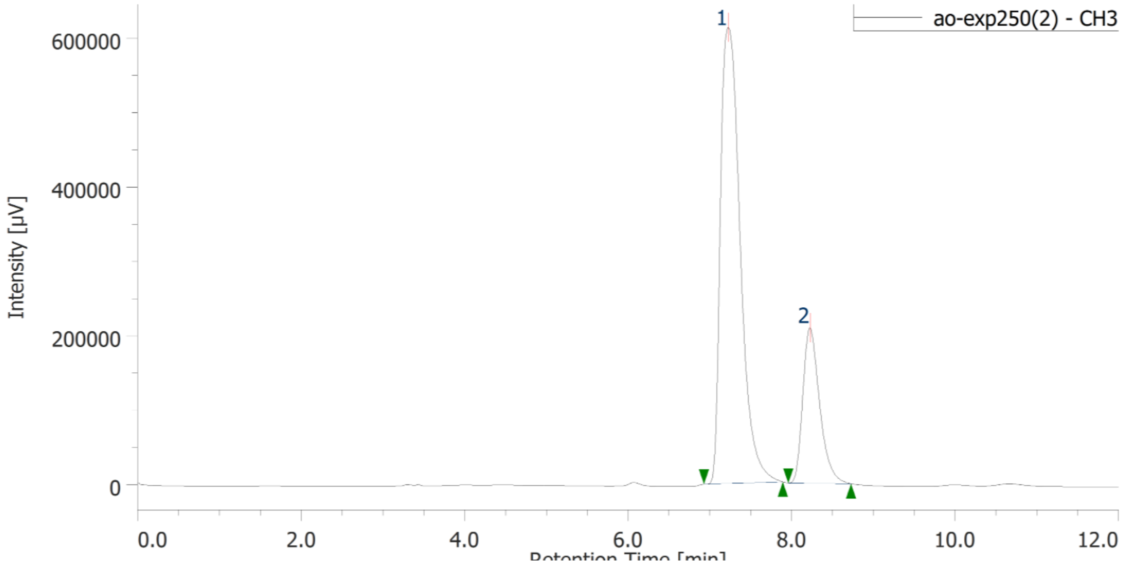

#	ピーク名	CH	tR [min]	面積 [µV·sec]	高さ [µV]	面積%	高さ%
1	Unknown	3	7.225	10291138	612648	77.5	74.6
2	Unknown	3	8.225	2987784	208907	22.5	25.4

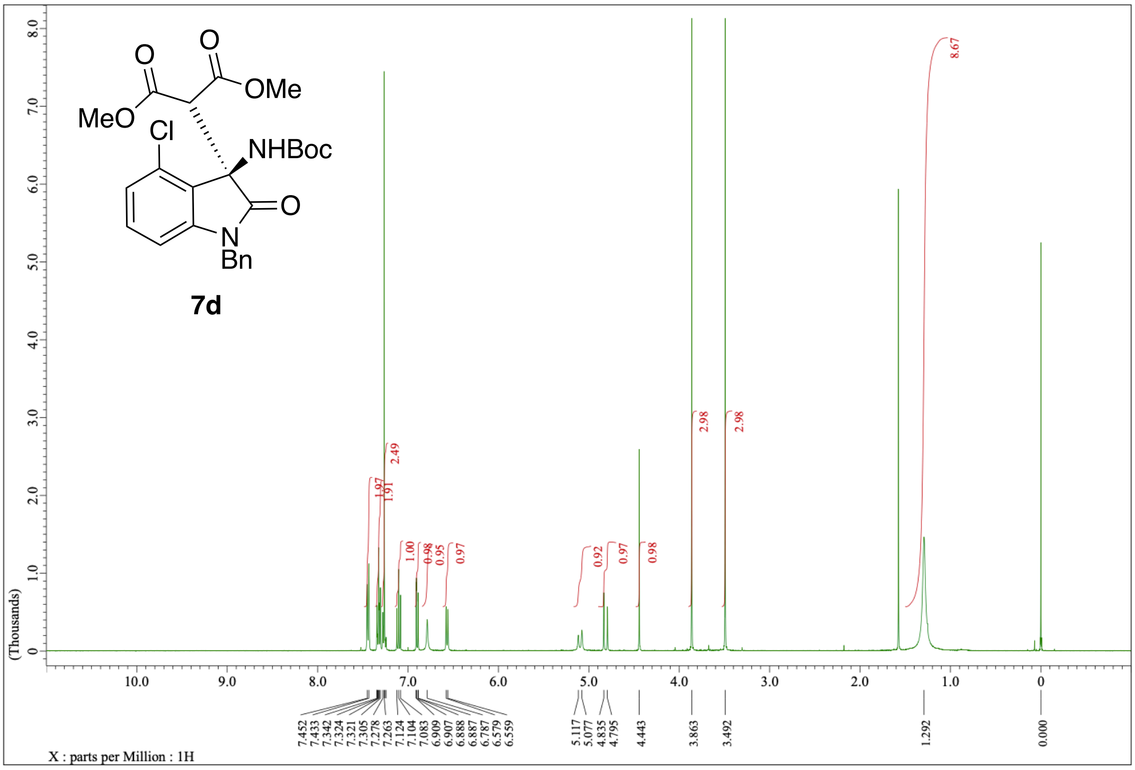
O
O
OMe
MeO
Cl
NHBoc
O
N
Bn
7d
(Thousands)
X : parts per Million : 1H
7.452
7.433
7.342
7.324
7.321
7.305
7.278
7.263
7.124
7.104
7.083
6.909
6.907
6.888
6.887
6.787
6.579
6.559
5.117
5.077
4.835
4.795
4.443
3.863
3.492
1.292
0.000
1.97
1.91
2.49
1.00
0.98
0.95
0.97
0.92
0.97
0.98
2.98
2.98
8.67

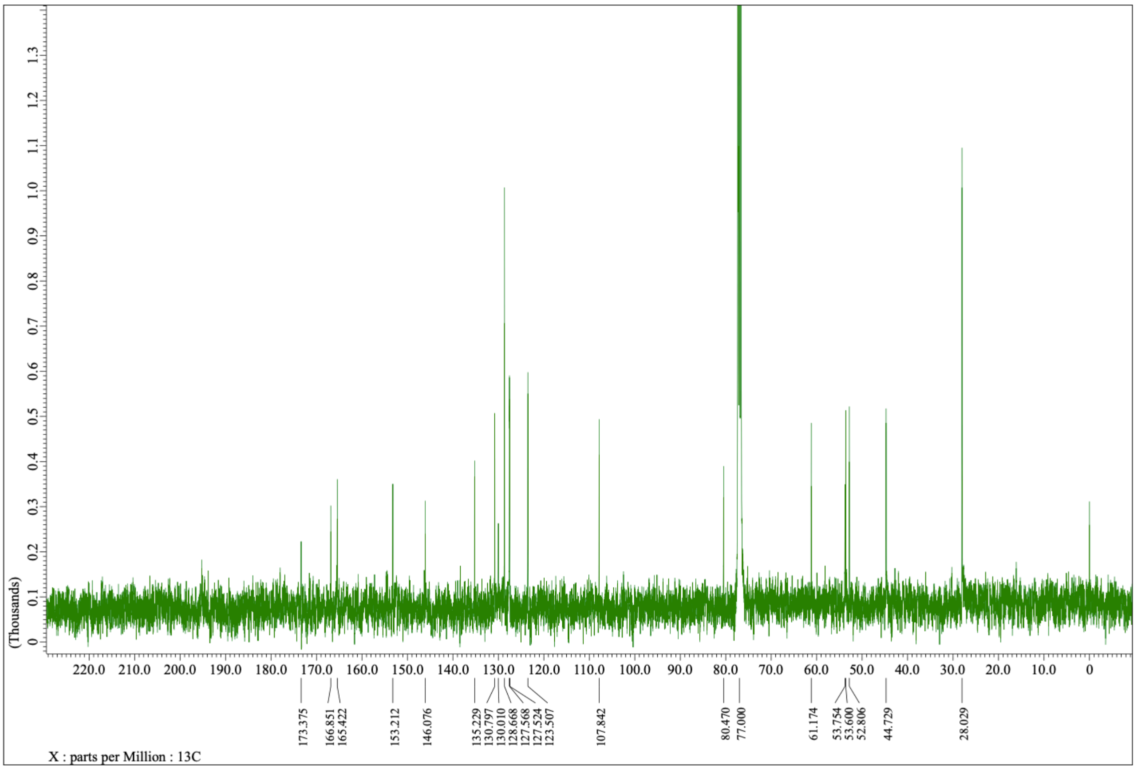
(Thousands)
X : parts per Million : 13C
173.375
166.851
165.422
153.212
146.076
135.229
130.797
130.010
128.668
127.568
127.524
123.507
107.842
80.470
77.000
61.174
53.754
53.600
52.806
44.729
28.029

Racemic sample of **7d**

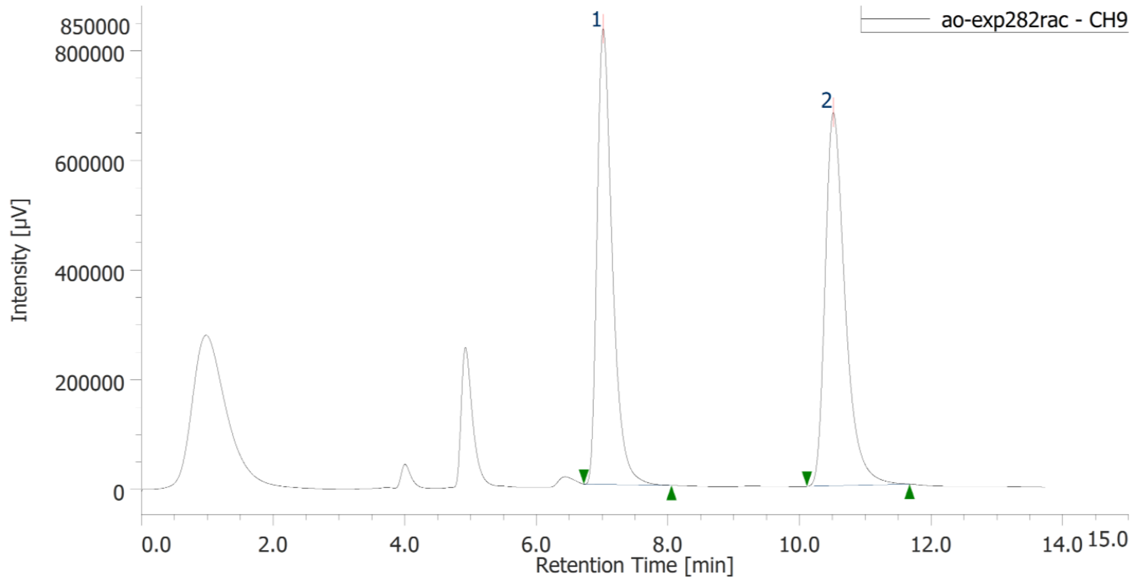

#	ピーク名	CH	tR [min]	面積 [µV·sec]	高さ [µV]	面積%	高さ%
1	Unknown	9	7.013	13387672	830499	48.2	55.0
2	Unknown	9	10.512	14376635	680124	51.8	45.0

7d catalyzed by **4a**

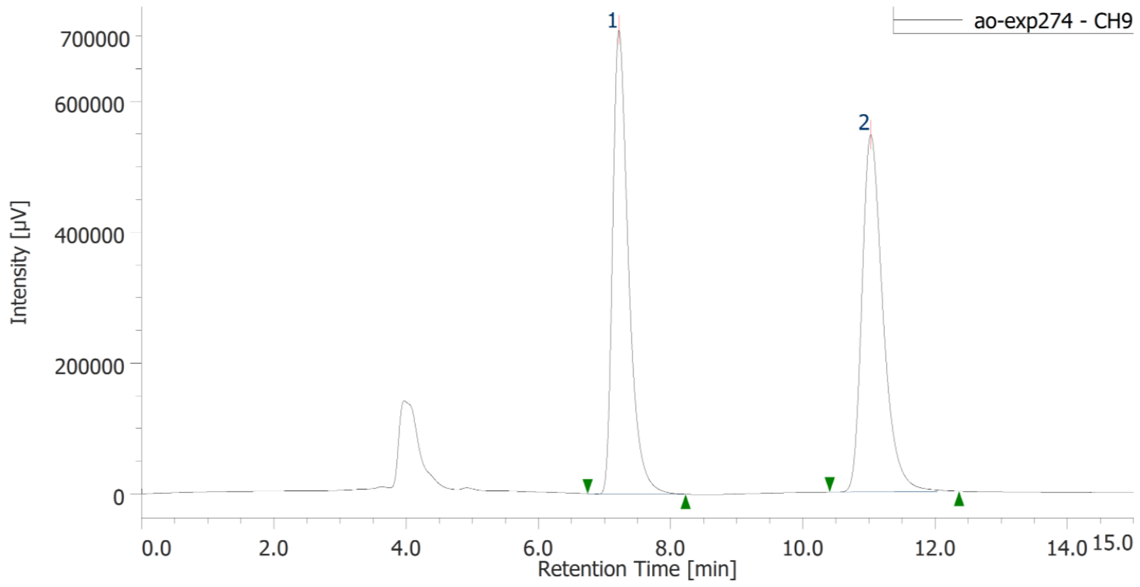

#	ピーク名	CH	tR [min]	面積 [µV·sec]	高さ [µV]	面積%	高さ%
1	Unknown	9	7.217	11804115	708841	49.4	56.5
2	Unknown	9	11.025	12071694	545843	50.6	43.5

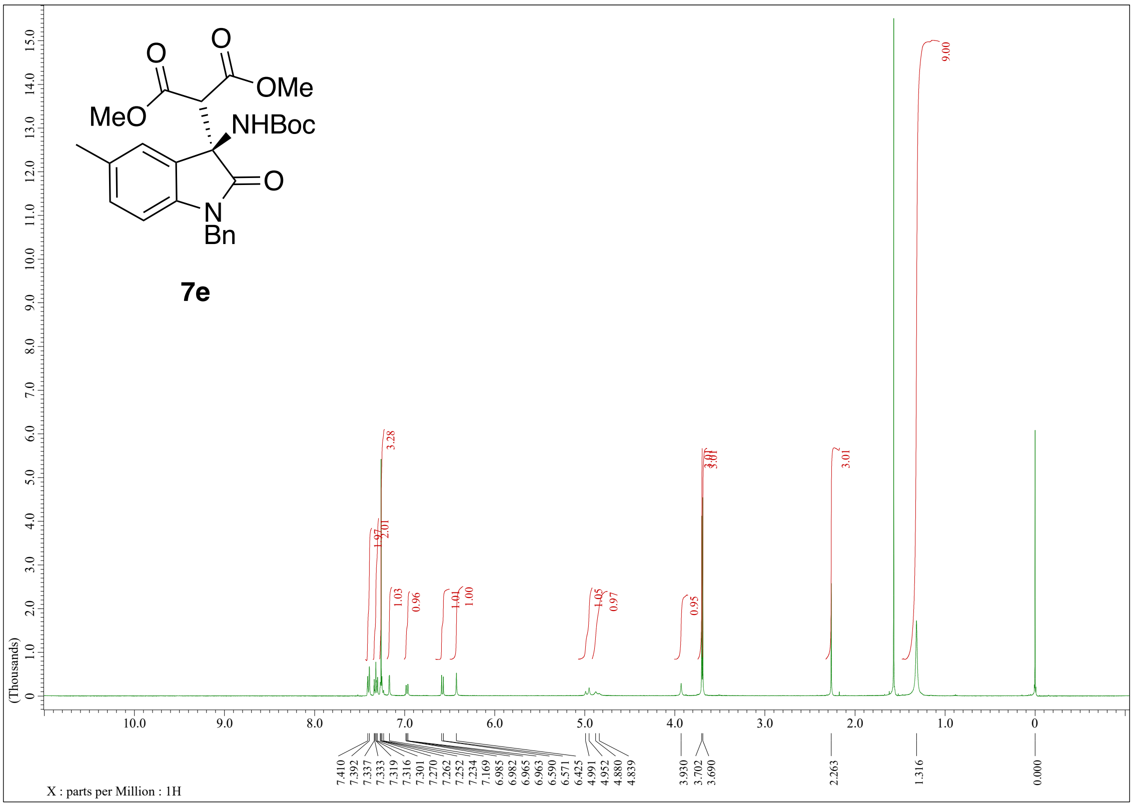

MeO
OMe
NHBoc
N
Bn
7e
(Thousands)
X : parts per Million : 1H
7.410
7.392
7.337
7.333
7.319
7.316
7.301
7.270
7.262
7.252
7.234
7.169
6.985
6.982
6.965
6.963
6.590
6.571
6.425
4.991
4.952
4.880
4.839
3.930
3.702
3.690
2.263
1.316
0.000
9.00
3.28
2.01
1.97
1.03
0.96
1.01
1.00
1.05
0.97
0.95
3.01

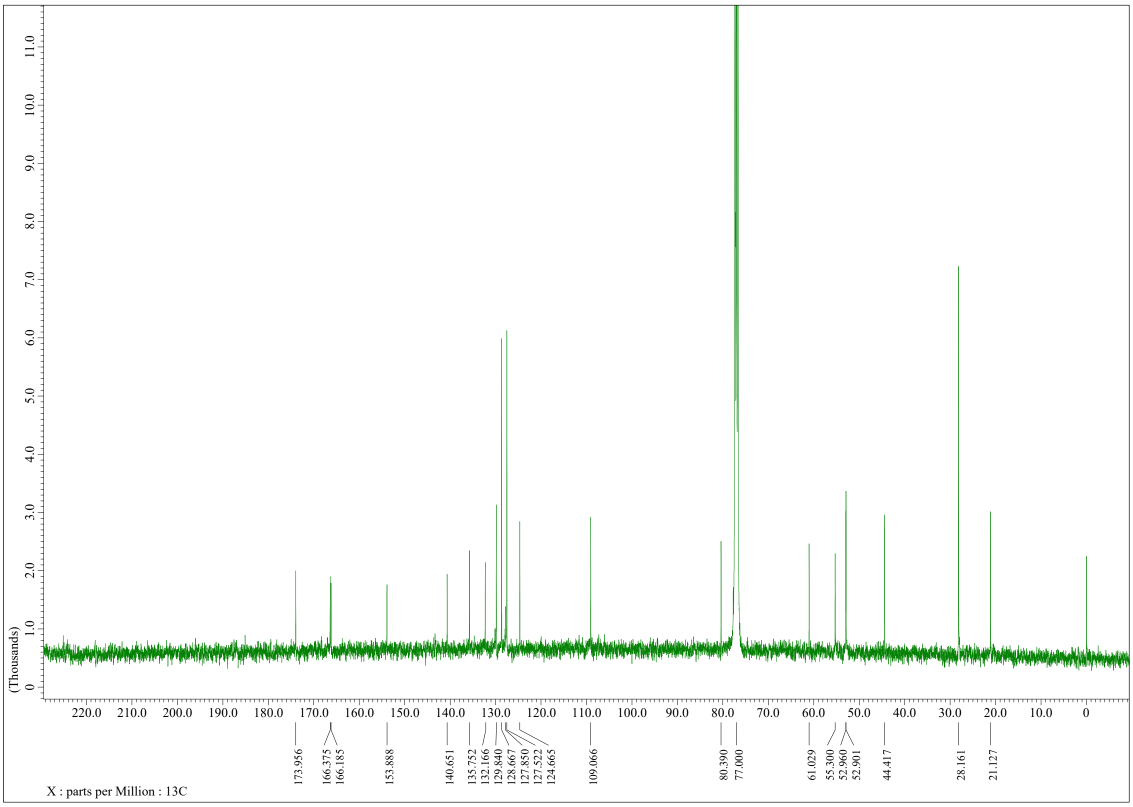

(Thousands)
X : parts per Million : 13C
173.956
166.375
166.185
153.888
140.651
135.752
132.166
129.840
128.667
127.850
127.522
124.665
109.066
80.390
77.000
61.029
55.300
52.960
52.901
44.417
28.161
21.127

Racemic sample of **7e**

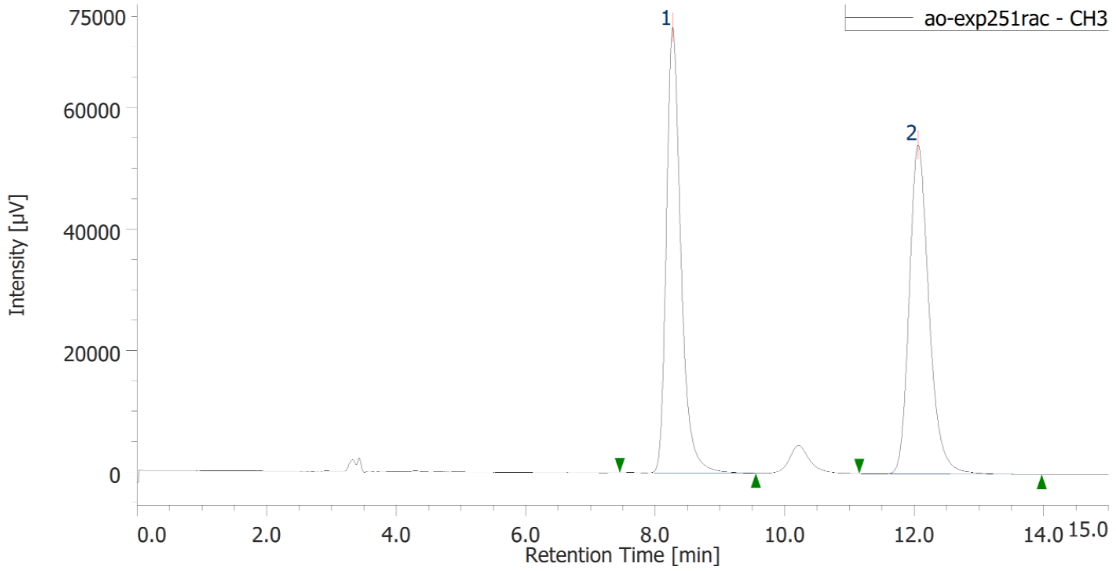

#	ピーク名	CH	tR [min]	面積 [μV·sec]	高さ [μV]	面積%	高さ%
1	Unknown	3	8.267	1141384	73322	49.9	57.5
2	Unknown	3	12.058	1146791	54163	50.1	42.5

7e catalyzed by **4a**

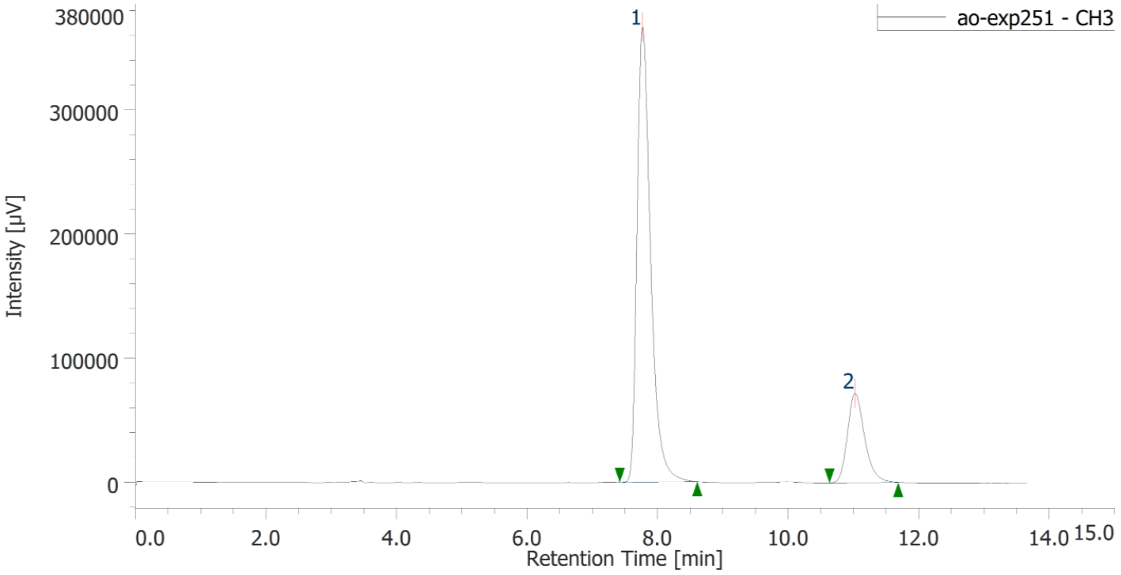

#	ピーク名	CH	tR [min]	面積 [μV·sec]	高さ [μV]	面積%	高さ%
1	Unknown	3	7.767	5311506	366736	80.2	83.6
2	Unknown	3	11.025	1311860	72001	19.8	16.4

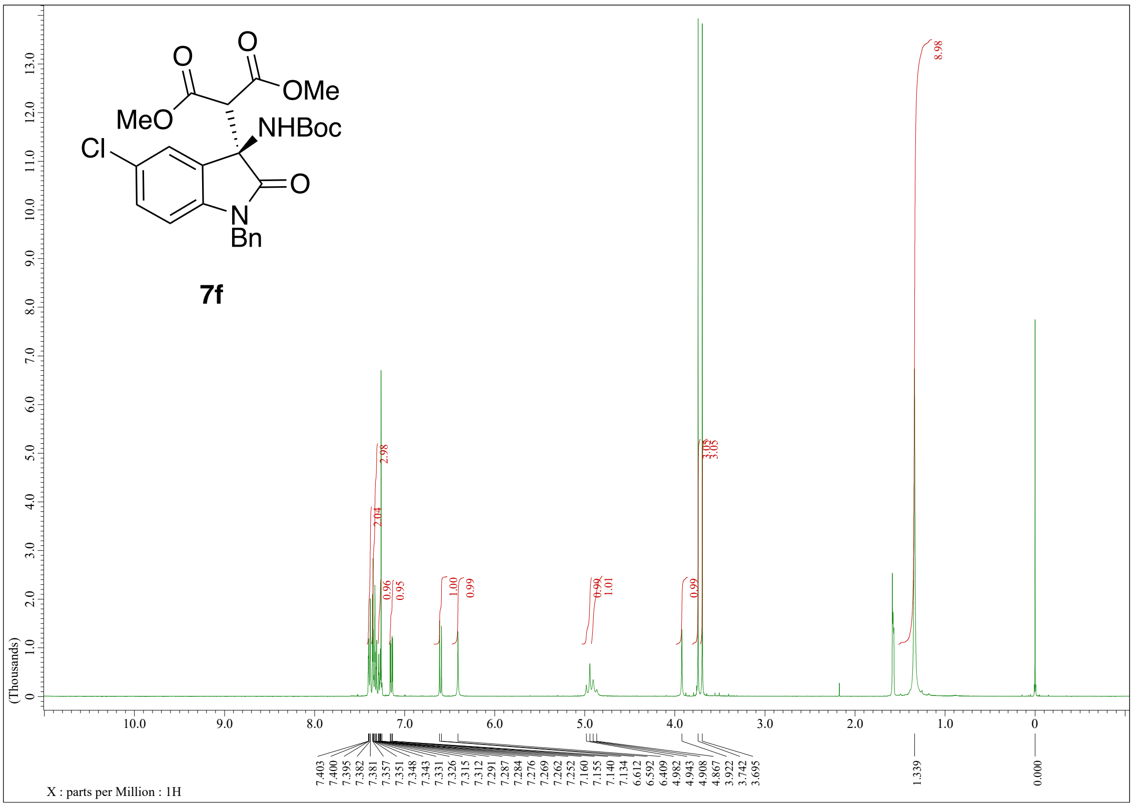

MeO
O
O
OMe
NHBoc
Cl
O
N
Bn
7f
X : parts per Million : 1H
(Thousands)

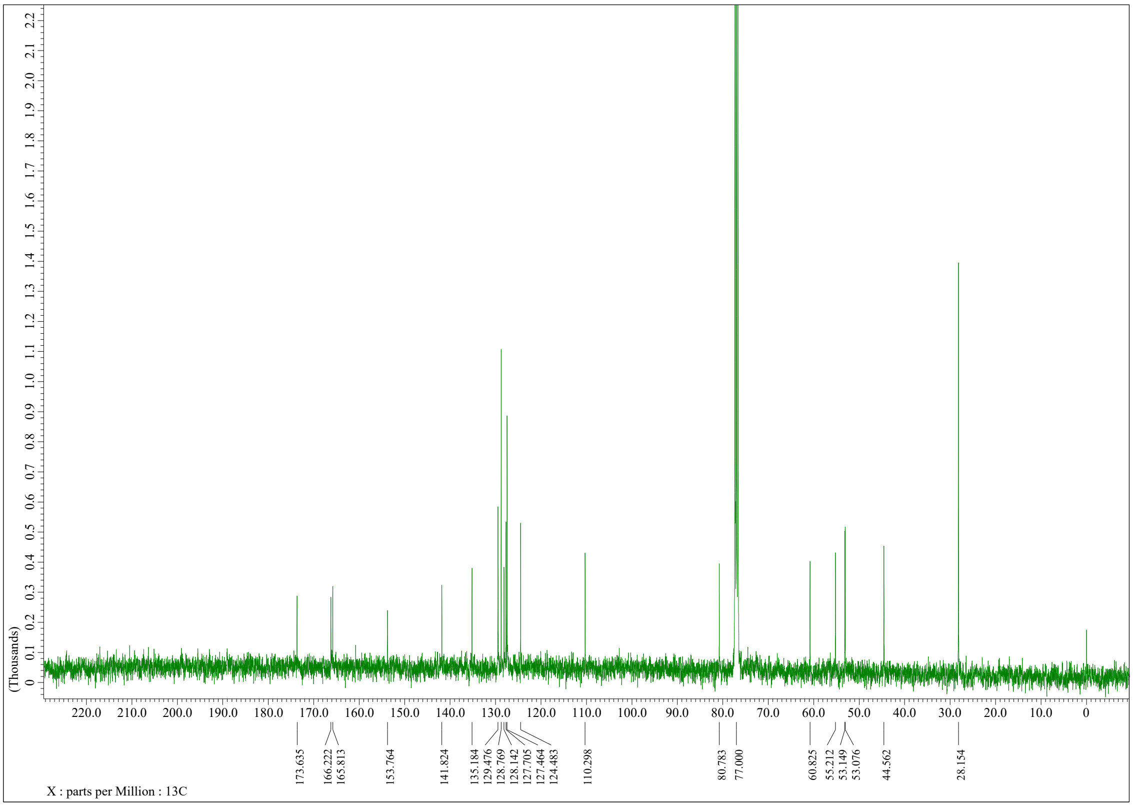

X : parts per Million : 13C
(Thousands)
173.635
166.222
165.813
153.764
141.824
135.184
129.476
128.769
128.142
127.705
127.464
124.483
110.298
80.783
77.000
60.825
55.212
53.149
53.076
44.562
28.154

Racemic sample of **7f**

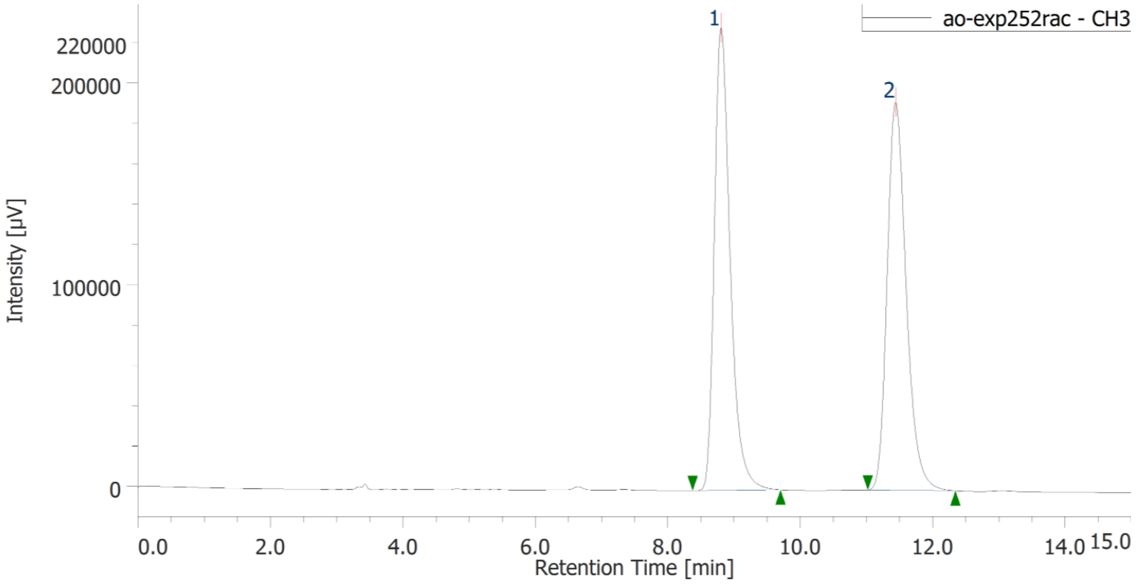

#	ピーク名	CH	tR [min]	面積 [μV·sec]	高さ [μV]	面積%	高さ%
1	Unknown	3	8.800	3818785	229343	49.8	54.4
2	Unknown	3	11.442	3842662	192245	50.2	45.6

7f catalyzed by **4a**

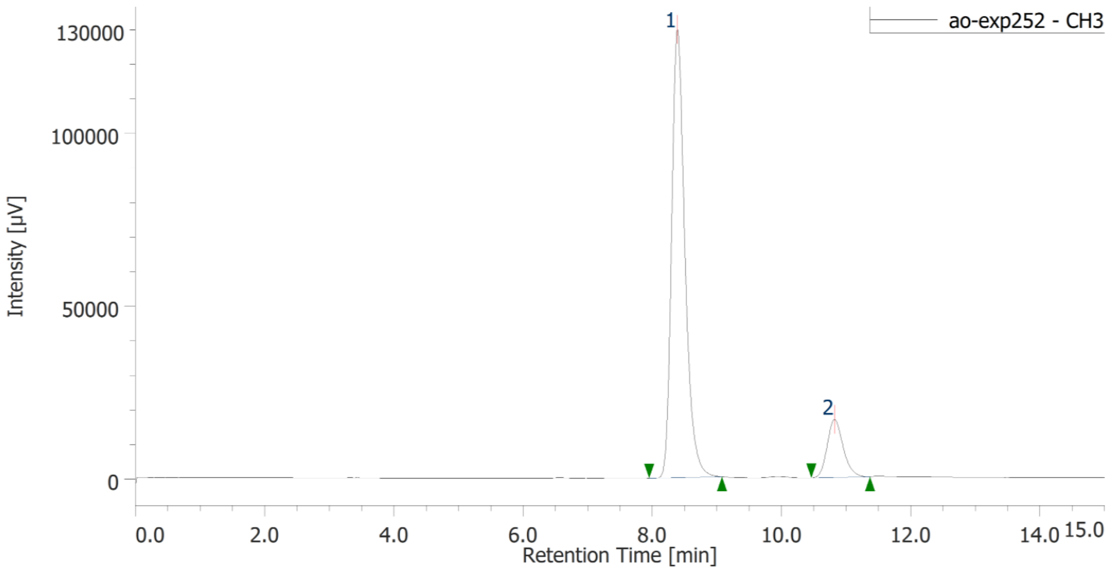

#	ピーク名	CH	tR [min]	面積 [μV·sec]	高さ [μV]	面積%	高さ%
1	Unknown	3	8.383	1850543	129527	86.6	88.5
2	Unknown	3	10.817	287346	16797	13.4	11.5

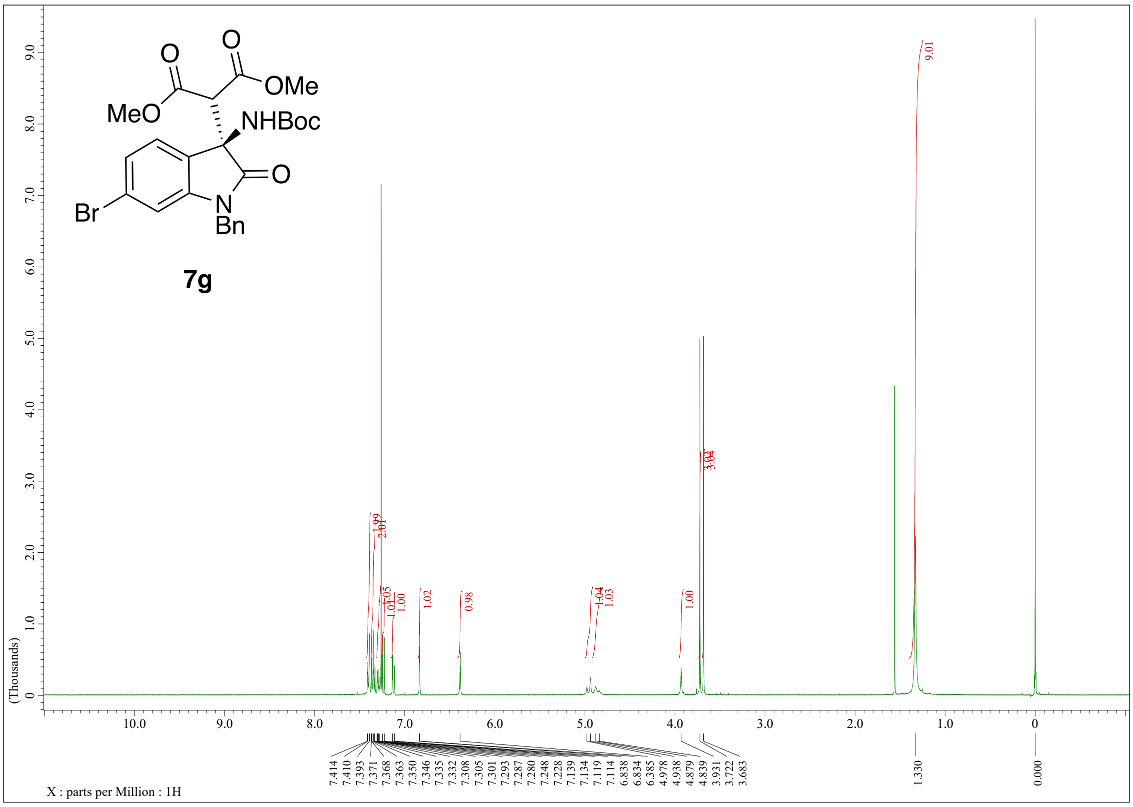

O
O
OMe
MeO
NHBoc
O
Br
N
Bn
7g
9.01
3.04
3.03
1.99
2.01
1.05
1.01
1.00
1.02
0.98
1.04
1.03
1.00
(Thousands)
7.414
7.410
7.393
7.371
7.368
7.363
7.350
7.346
7.335
7.332
7.308
7.305
7.301
7.293
7.287
7.280
7.248
7.228
7.139
7.134
7.119
7.114
6.838
6.834
6.385
4.978
4.938
4.879
4.839
3.931
3.722
3.683
1.330
0.000
X : parts per Million : 1H

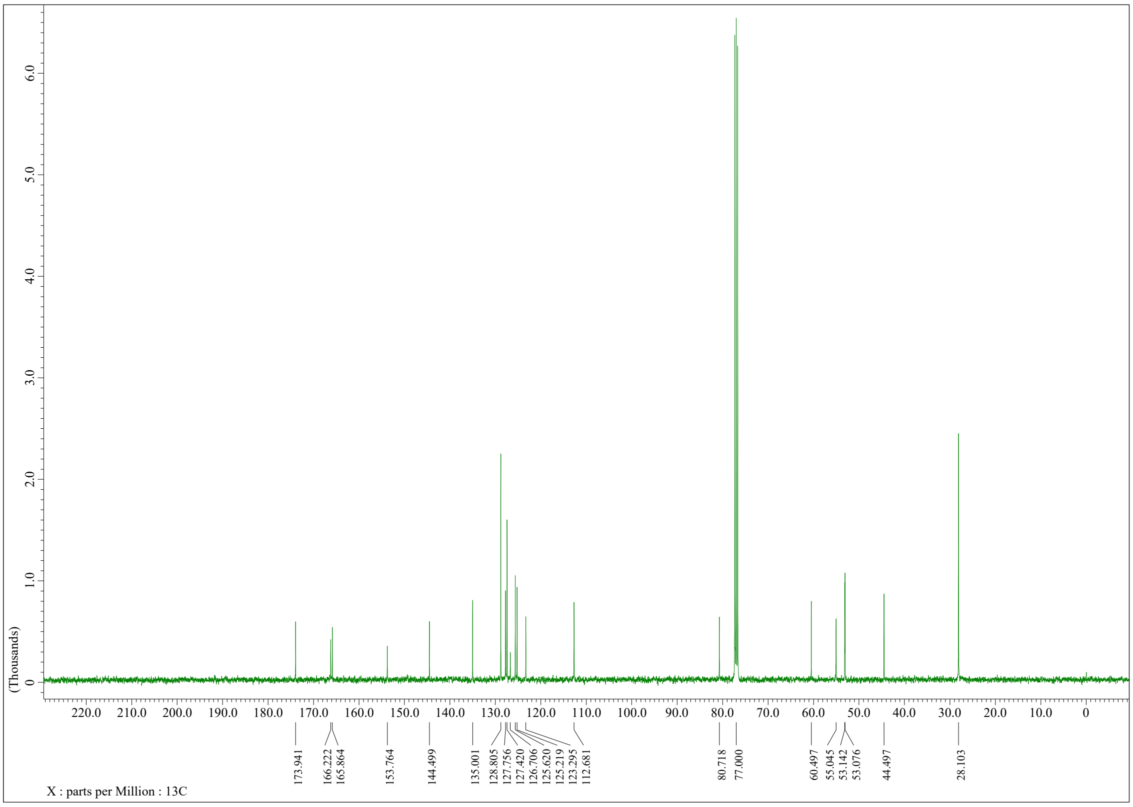

(Thousands)
173.941
166.222
165.864
153.764
144.499
135.001
128.805
127.756
127.420
126.706
125.620
125.219
123.295
112.681
80.718
77.000
60.497
55.045
53.142
53.076
44.497
28.103
X : parts per Million : 13C

Racemic sample of **7g**

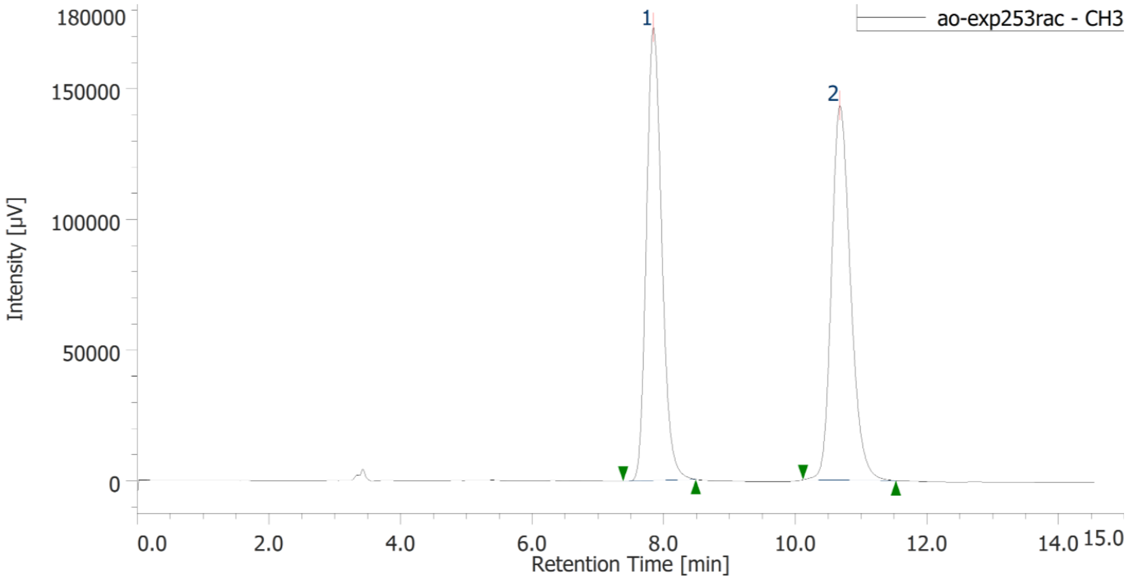

#	ピーク名	CH	tR [min]	面積 [μV·sec]	高さ [μV]	面積%	高さ%
1	Unknown	3	7.842	2808773	173331	49.3	54.7
2	Unknown	3	10.675	2886473	143286	50.7	45.3

7g catalyzed by **4a**

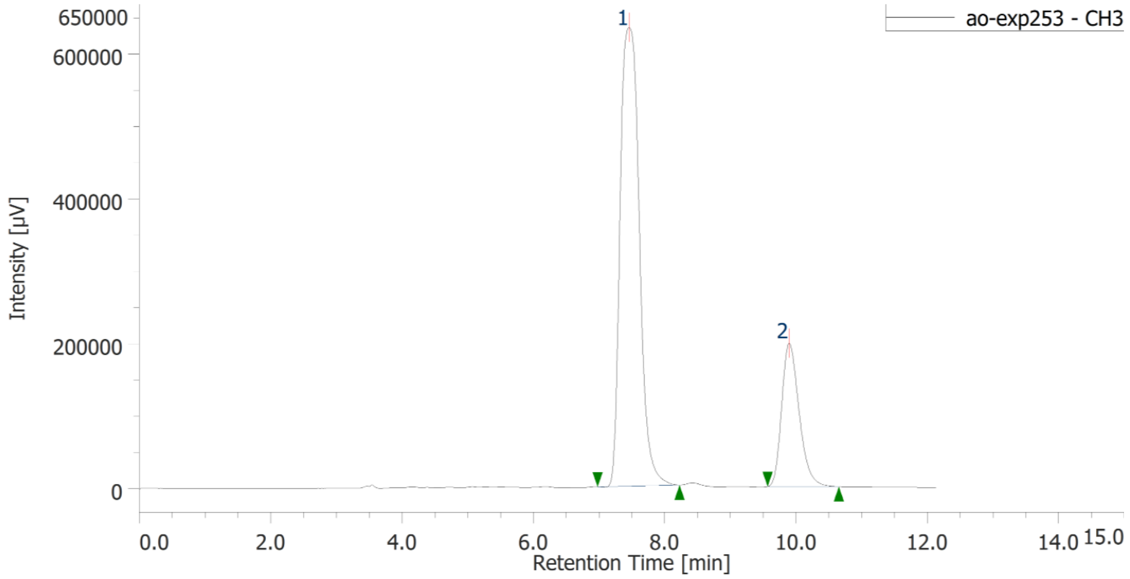

#	ピーク名	CH	tR [min]	面積 [μV·sec]	高さ [μV]	面積%	高さ%
1	Unknown	3	7.458	12827146	633069	77.9	76.2
2	Unknown	3	9.892	3647570	197759	22.1	23.8

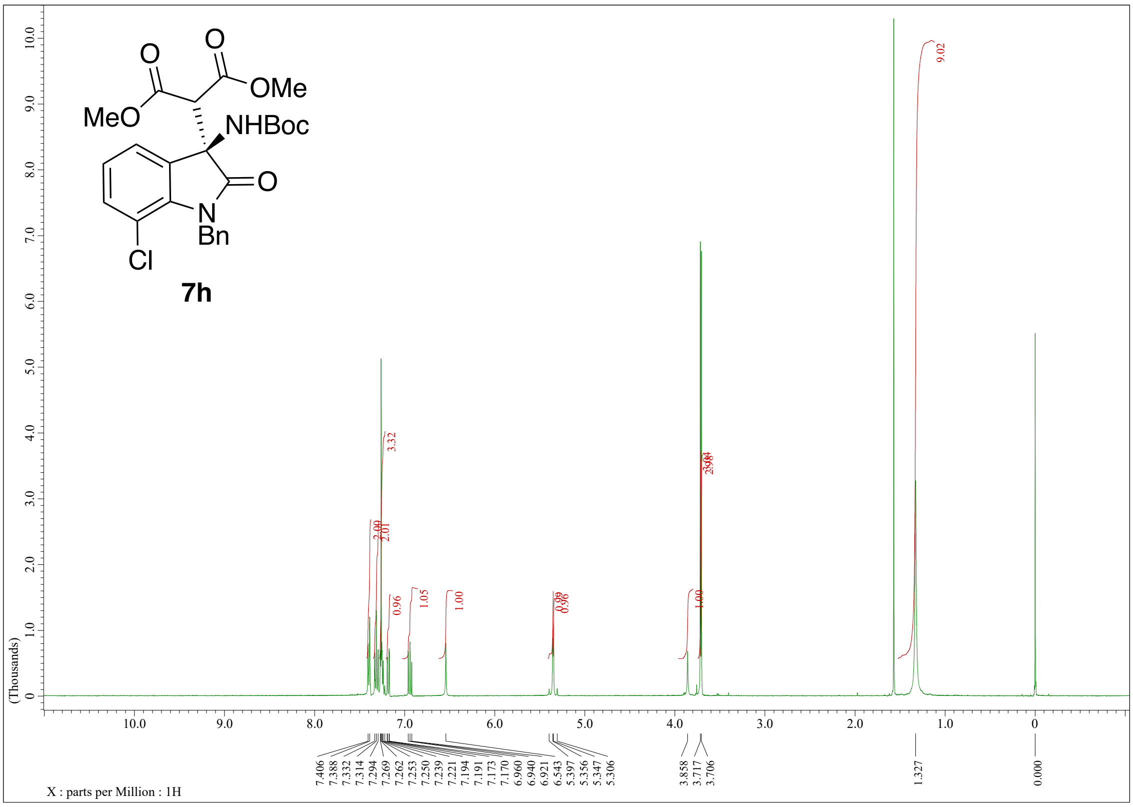
O
O
OMe
MeO
NHBoc
O
N
Bn
Cl
7h
(Thousands)
X : parts per Million : 1H
7.406
7.388
7.332
7.314
7.294
7.269
7.262
7.253
7.250
7.239
7.221
7.194
7.191
7.173
7.170
6.960
6.940
6.921
6.543
5.397
5.356
5.347
5.306
3.858
3.717
3.706
1.327
0.000
9.02
3.32
2.00
2.01
0.96
1.05
1.00
0.96
1.00

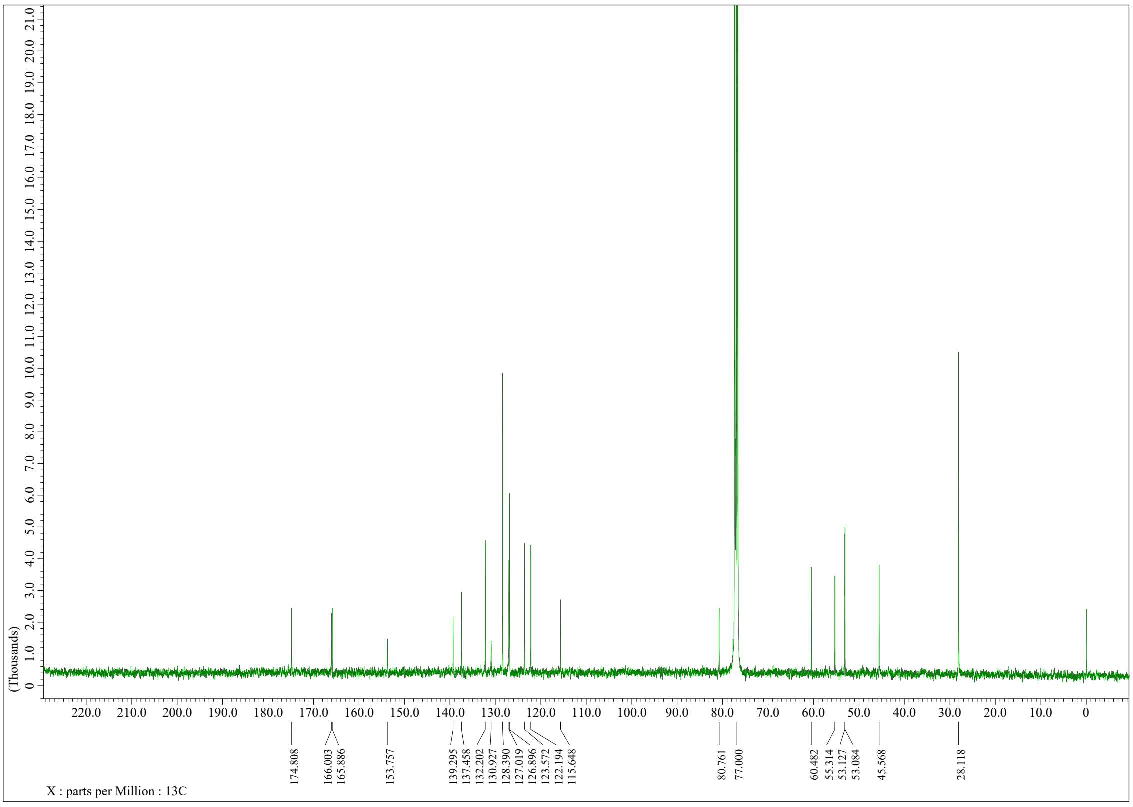
(Thousands)
X : parts per Million : 13C
174.808
166.003
165.886
153.757
139.295
137.458
132.202
130.927
128.390
127.019
126.896
123.572
122.194
115.648
80.761
77.000
60.482
55.314
53.127
53.084
45.568
28.118

Racemic sample of **7h**

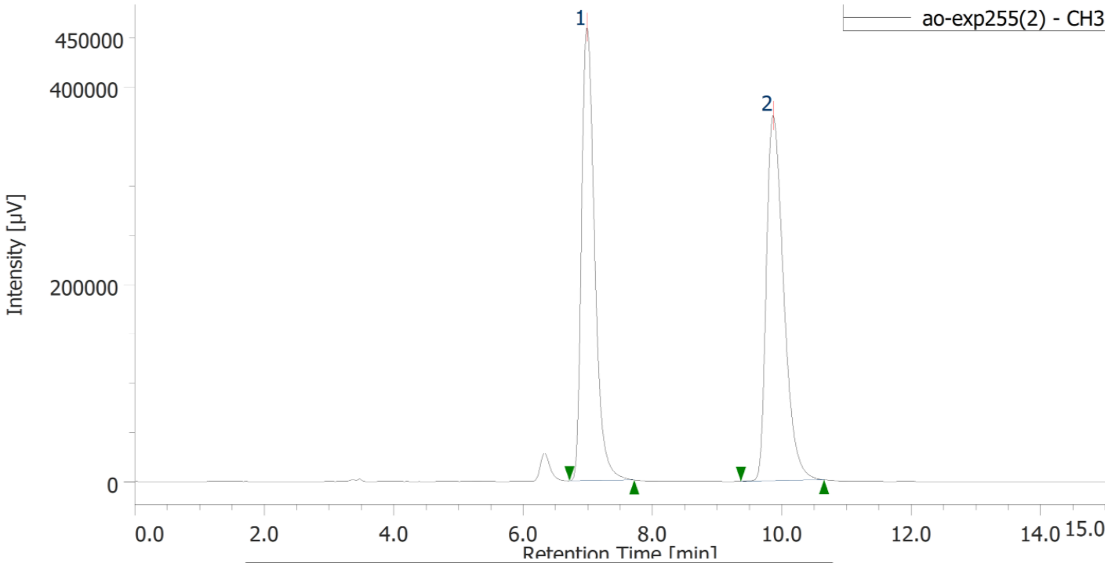

#	ピーク名	CH	tR [min]	面積 [μV·sec]	高さ [μV]	面積%	高さ%
1	Unknown	3	6.983	6475453	458736	48.5	55.4
2	Unknown	3	9.867	6863733	369520	51.5	44.6

7h catalyzed by **4a**

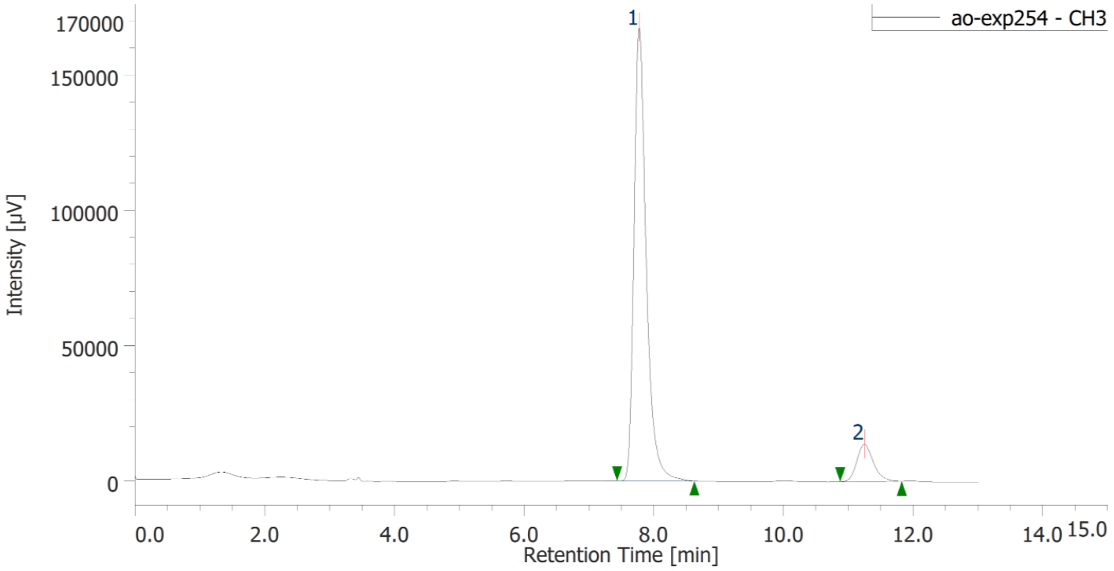

#	ピーク名	CH	tR [min]	面積 [μV·sec]	高さ [μV]	面積%	高さ%
1	Unknown	3	7.775	2195658	167722	90.0	92.4
2	Unknown	3	11.250	243390	13755	9.98	7.58

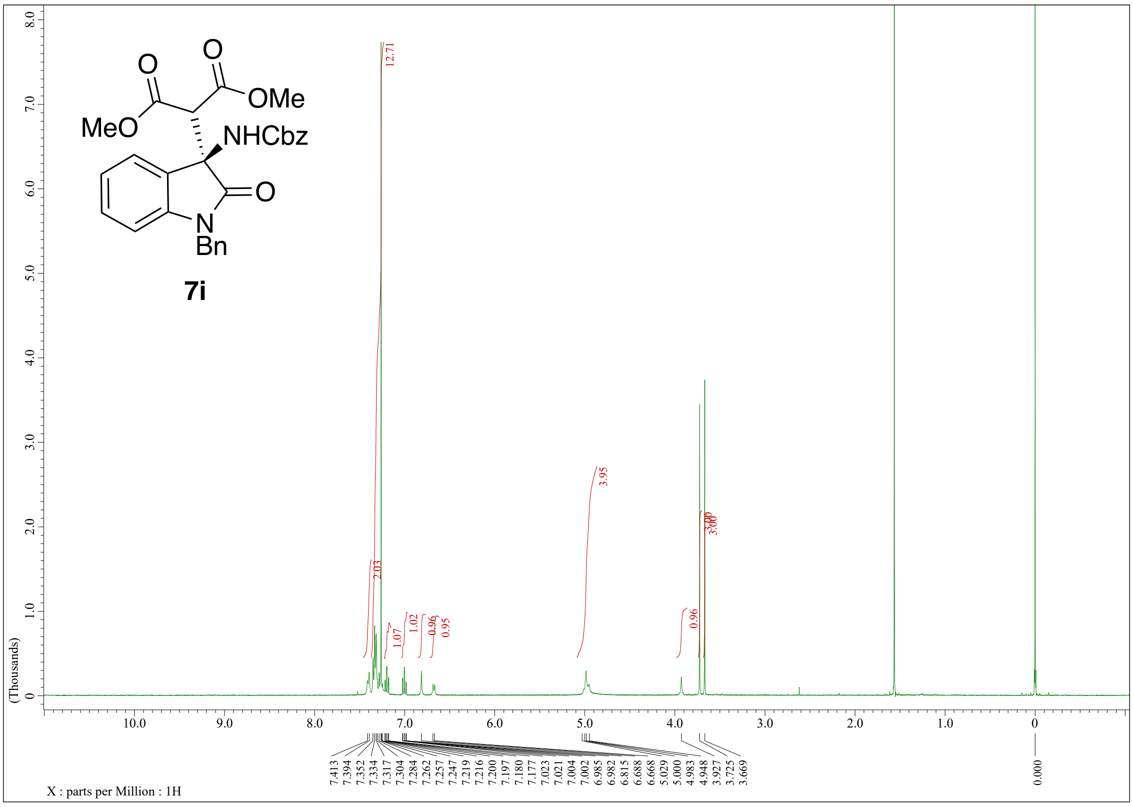

MeO
OMe
NHCbz
N
Bn
7i
12.71
2.03
1.07
1.02
0.96
0.95
3.95
0.96
3.00
(Thousands)
X : parts per Million : 1H
7.413
7.394
7.352
7.334
7.317
7.304
7.284
7.262
7.257
7.247
7.219
7.216
7.200
7.197
7.180
7.177
7.023
7.021
7.004
7.002
6.985
6.982
6.815
6.688
6.668
5.029
5.000
4.983
4.948
3.927
3.725
3.669
0.000

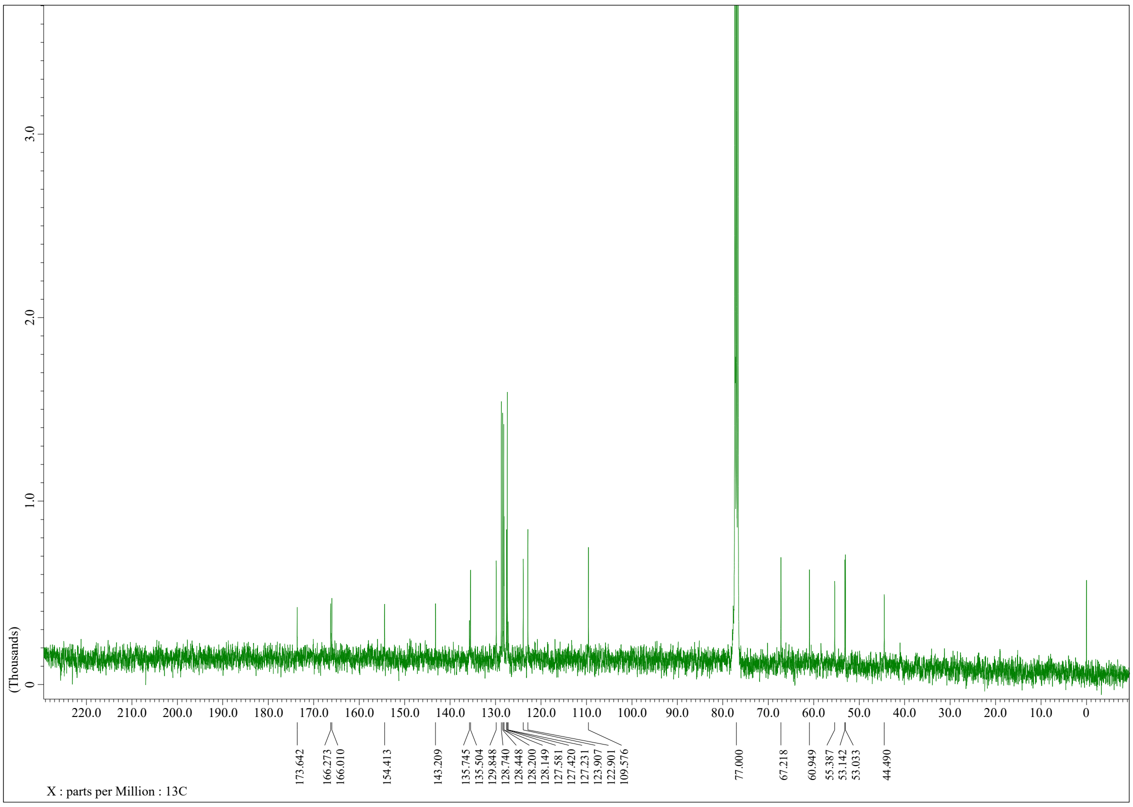

(Thousands)
X : parts per Million : 13C
173.642
166.273
166.010
154.413
143.209
135.745
135.504
129.848
128.740
128.448
128.200
128.149
127.581
127.420
127.231
123.907
122.901
109.576
77.000
67.218
60.949
55.387
53.142
53.033
44.490

Racemic sample of **7i**

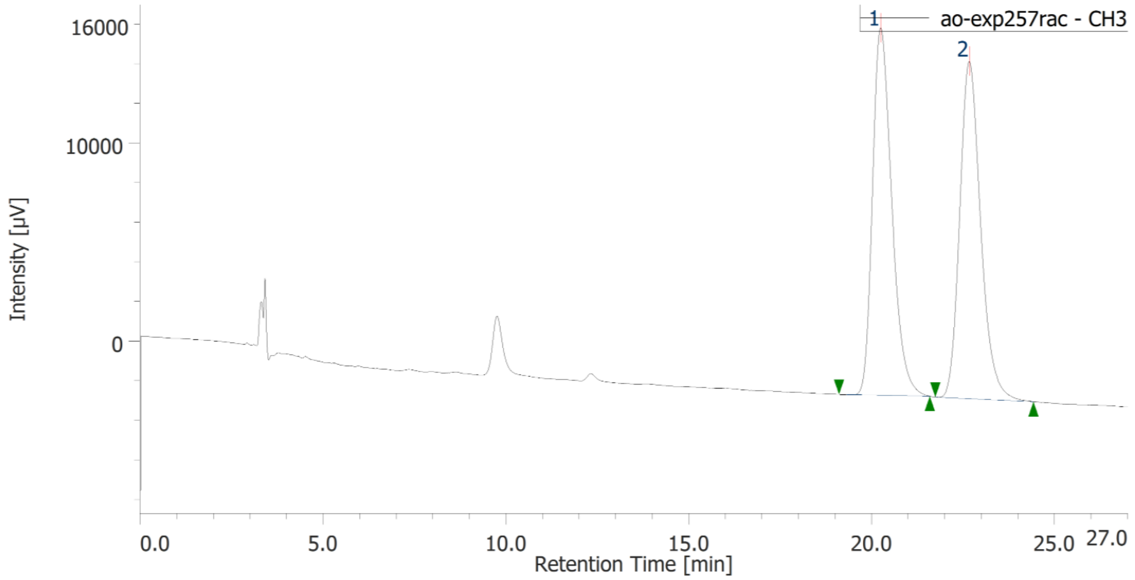

#	ピーク名	CH	tR [min]	面積 [μV·sec]	高さ [μV]	面積%	高さ%
1	Unknown	3	20.242	678230	18537	50.1	52.1
2	Unknown	3	22.667	674899	17018	49.9	47.9

7i catalyzed by **4a**

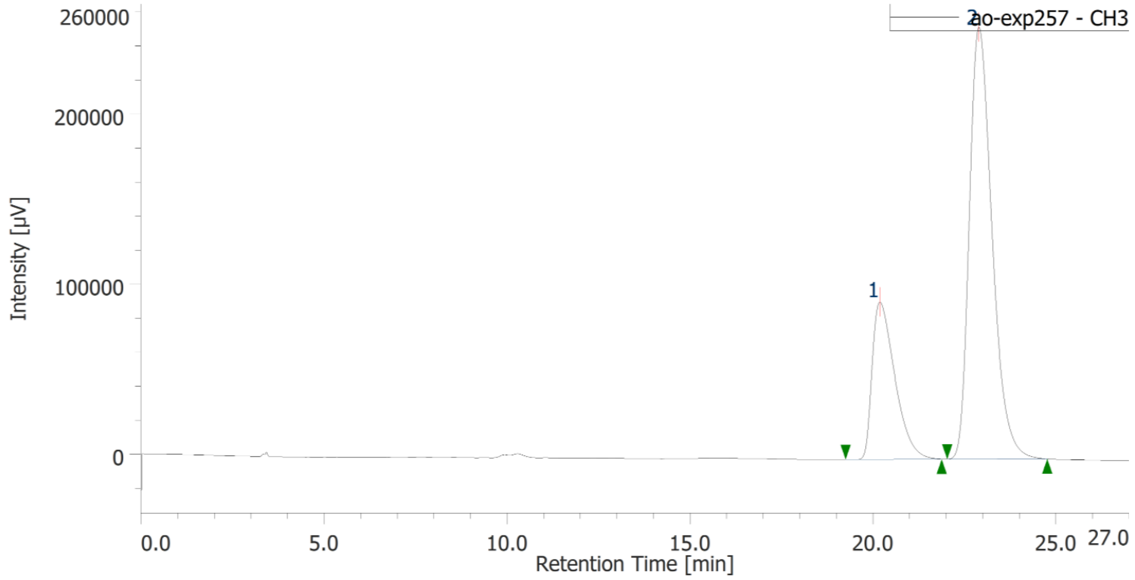

#	ピーク名	CH	tR [min]	面積 [μV·sec]	高さ [μV]	面積%	高さ%
1	Unknown	3	20.183	3985252	92517	26.7	26.7
2	Unknown	3	22.883	10921682	253860	73.3	73.3

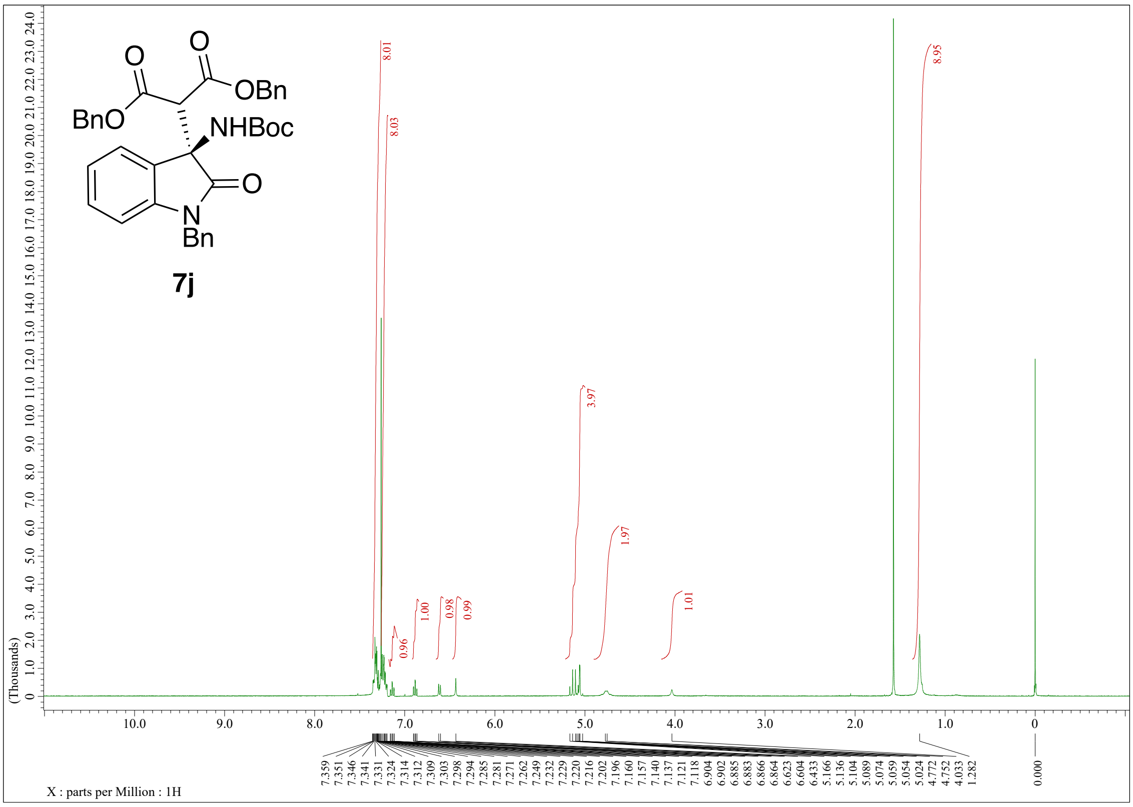

BnO
OBn
NHBoc
O
N
Bn
7j
(Thousands)
8.01
8.03
0.96
1.00
0.98
0.99
3.97
1.97
1.01
8.95
7.359 7.351 7.346 7.341 7.331 7.324 7.314 7.312 7.309 7.303 7.298 7.294 7.288 7.281 7.271 7.262 7.249 7.232 7.229 7.220 7.216 7.202 7.196 7.160 7.157 7.140 7.137 7.121 7.118 6.904 6.902 6.885 6.883 6.866 6.864 6.623 6.604 6.433 5.166 5.136 5.104 5.089 5.074 5.059 5.054 5.024 4.772 4.752 4.033 1.282
0.000
X : parts per Million : 1H

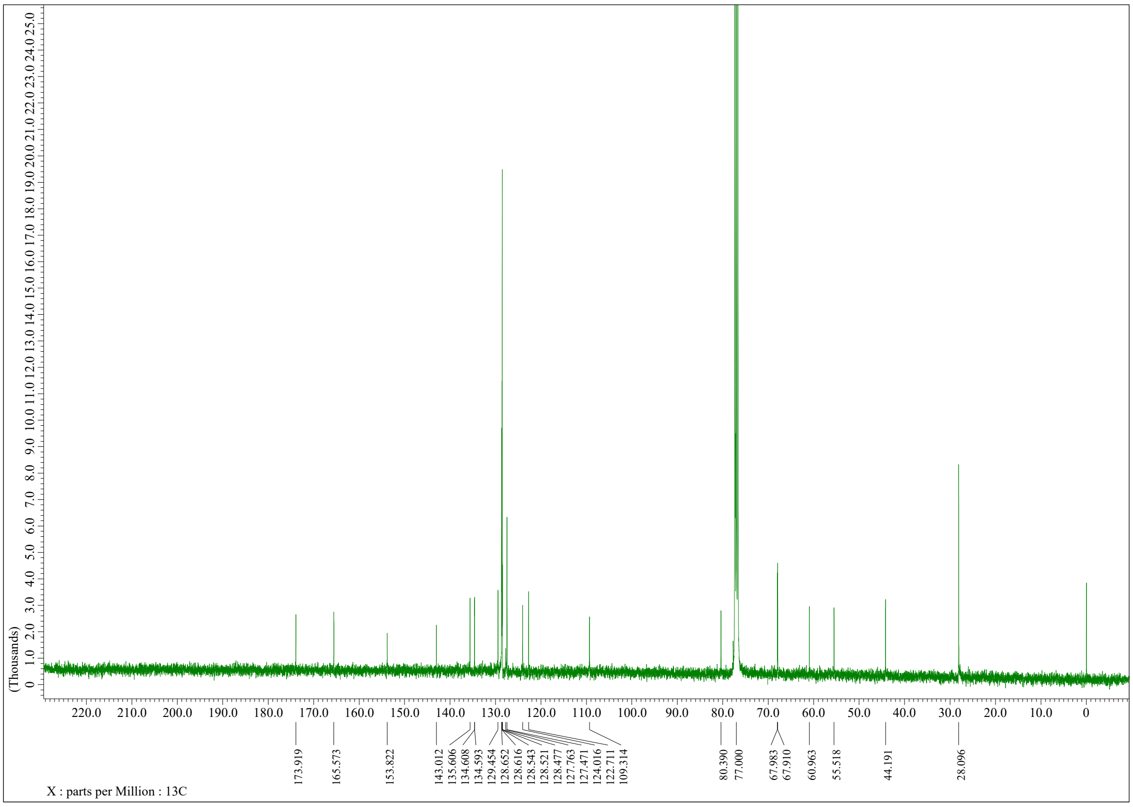

(Thousands)
173.919
165.573
153.822
143.012 135.606 134.608 134.593 129.454 128.652 128.616 128.543 128.521 128.477 127.763 127.471 124.016 122.711 109.314
80.390
77.000
67.983 67.910
60.963
55.518
44.191
28.096
X : parts per Million : 13C

Racemic sample of **7j**

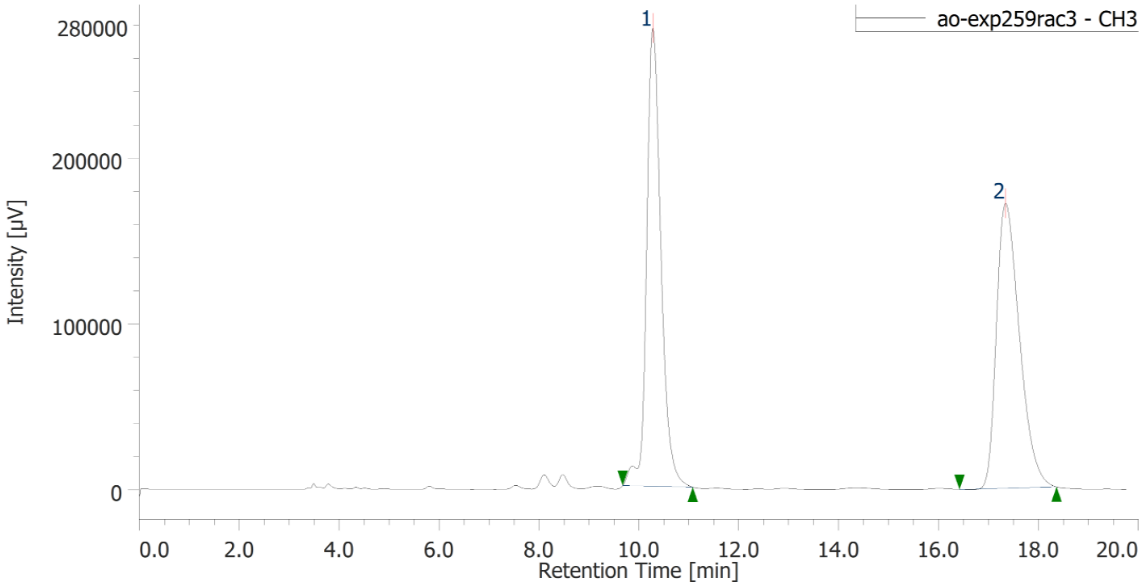

#	ピーク名	CH	tR [min]	面積 [μV·sec]	高さ [μV]	面積%	高さ%
1	Unknown	3	10.283	5506445	276038	50.1	61.7
2	Unknown	3	17.342	5494019	171610	49.9	38.3

7j catalyzed by **4a**

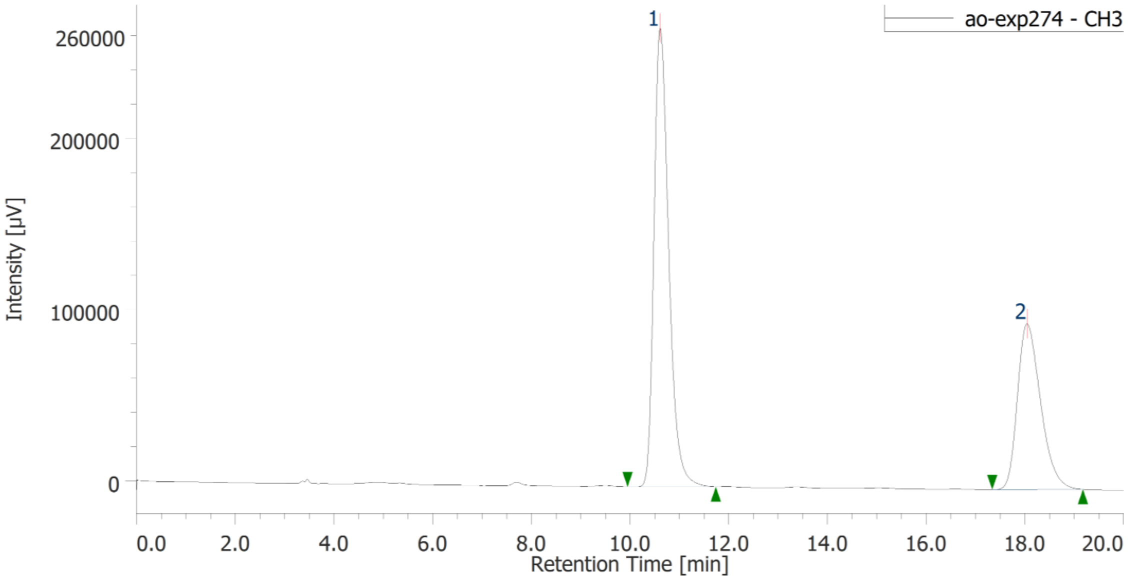

#	ピーク名	CH	tR [min]	面積 [μV·sec]	高さ [μV]	面積%	高さ%
1	Unknown	3	10.608	5541817	267545	63.8	73.5
2	Unknown	3	18.042	3149248	96697	36.2	26.5

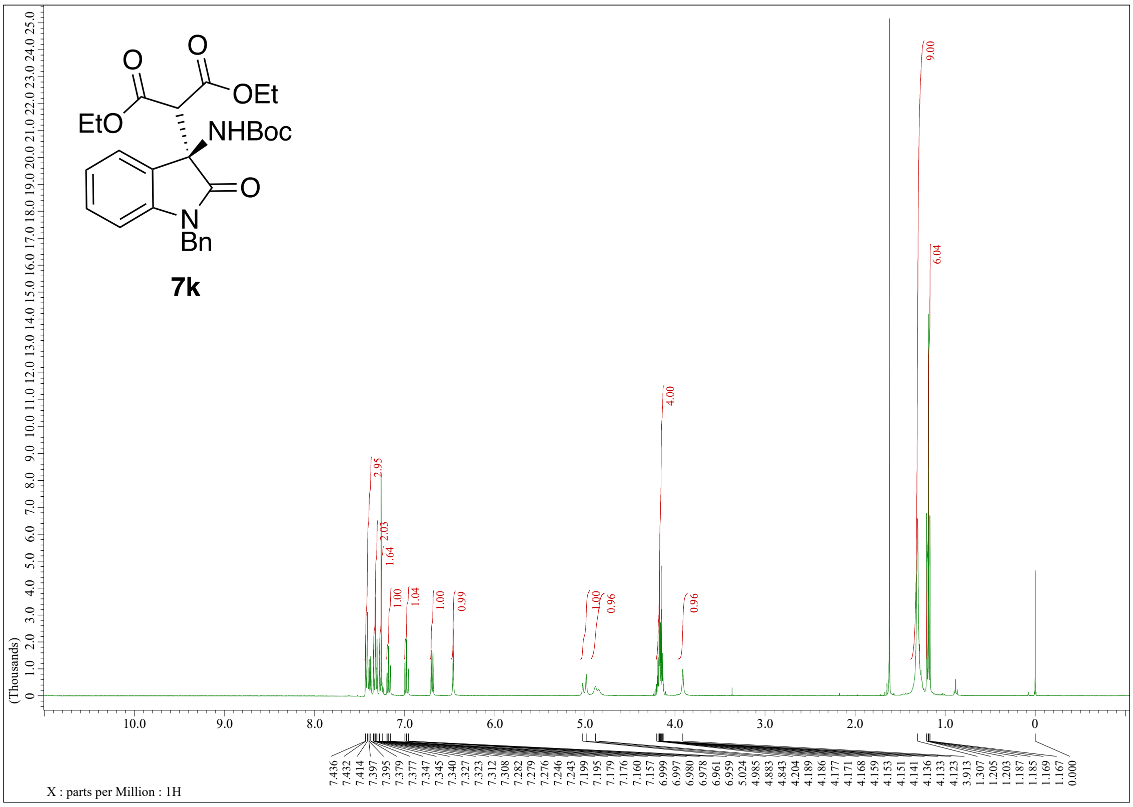
EtO
O
O
OEt
NHBoc
O
N
Bn
7k
(Thousands)
2.95
2.03
1.64
1.00
1.04
1.00
0.99
1.00
0.96
4.00
0.96
9.00
6.04
7.436 7.432 7.414 7.397 7.395 7.379 7.377 7.347 7.345 7.340 7.327 7.323 7.312 7.308 7.282 7.279 7.276 7.246 7.243 7.199 7.195 7.179 7.176 7.160 7.157 6.999 6.997 6.980 6.978 6.961 6.959 5.024 4.985 4.883 4.843 4.204 4.189 4.186 4.177 4.171 4.168 4.159 4.153 4.151 4.141 4.136 4.133 4.123 3.913 1.307 1.205 1.203 1.187 1.185 1.169 1.167 0.000
X : parts per Million : 1H

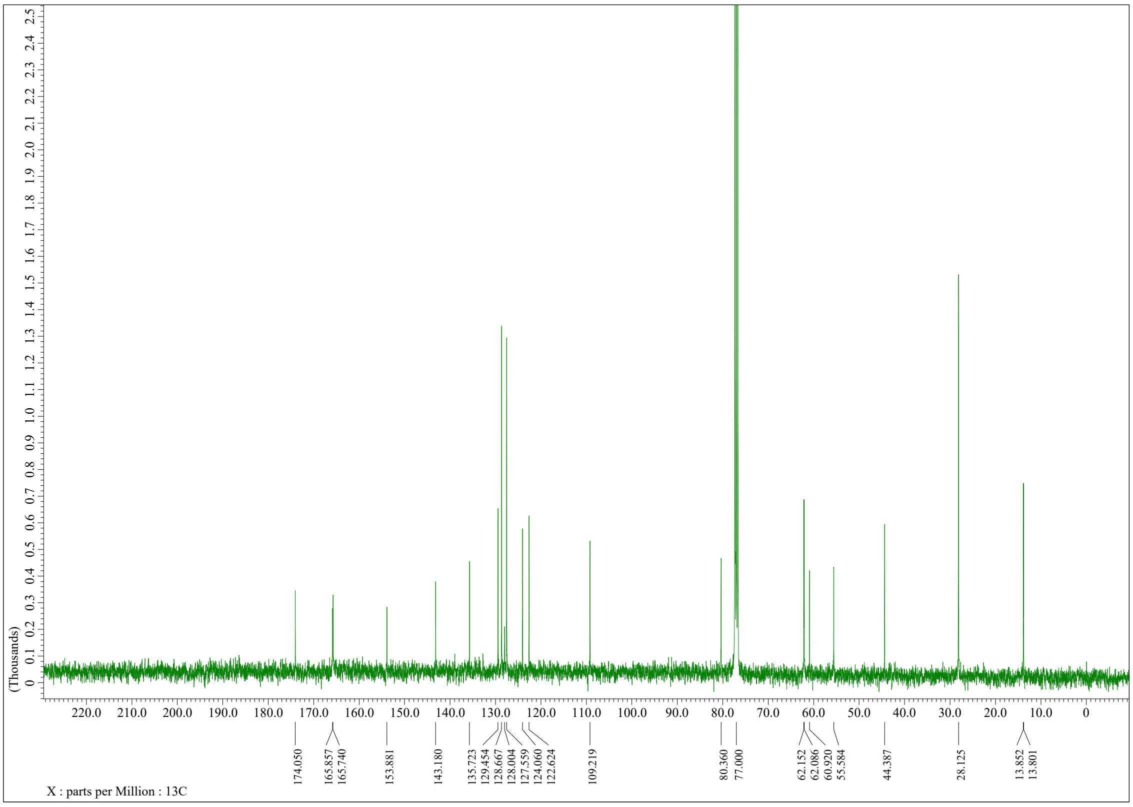
(Thousands)
174.050 165.857 165.740 153.881 143.180 135.723 129.454 128.667 128.004 127.559 124.060 122.624 109.219 80.360 77.000 62.152 62.086 60.920 55.584 44.387 28.125 13.852 13.801
X : parts per Million : 13C

Racemic sample of **7k**

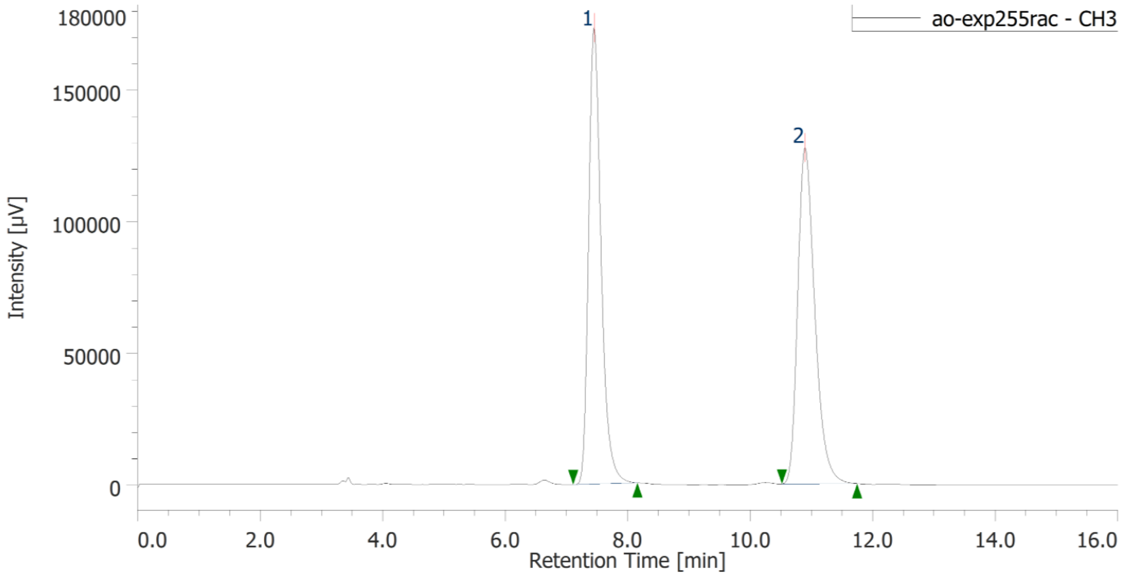

#	ピーク名	CH	tR [min]	面積 [µV·sec]	高さ [µV]	面積%	高さ%
1	Unknown	3	7.450	2450724	173241	49.8	57.6
2	Unknown	3	10.892	2467709	127557	50.2	42.4

7k catalyzed by **4a**

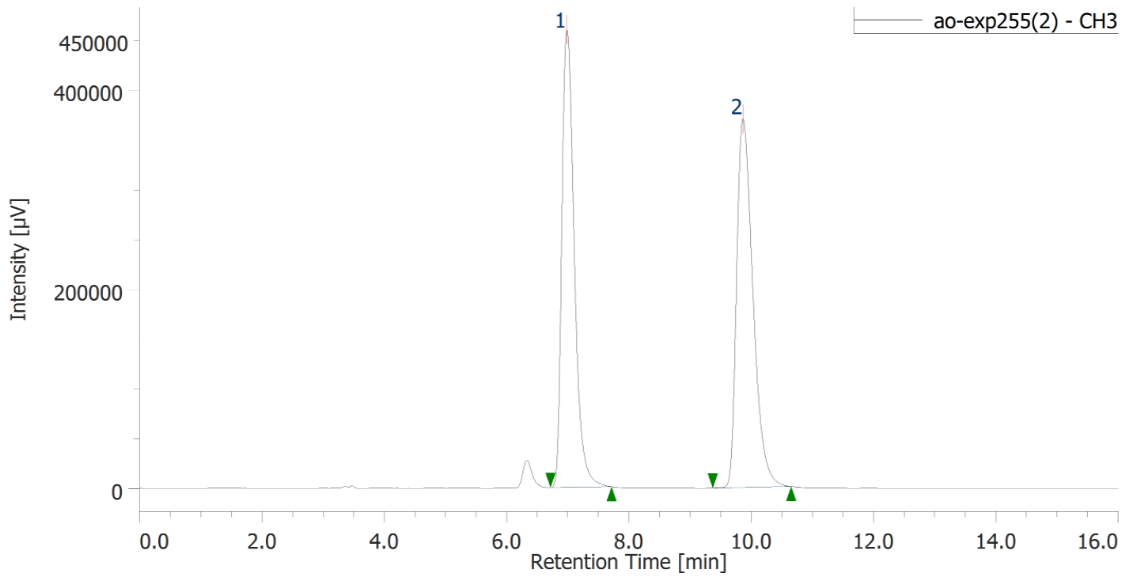

#	ピーク名	CH	tR [min]	面積 [µV·sec]	高さ [µV]	面積%	高さ%
1	Unknown	3	6.983	6475453	458736	48.5	55.4
2	Unknown	3	9.867	6863733	369520	51.5	44.6

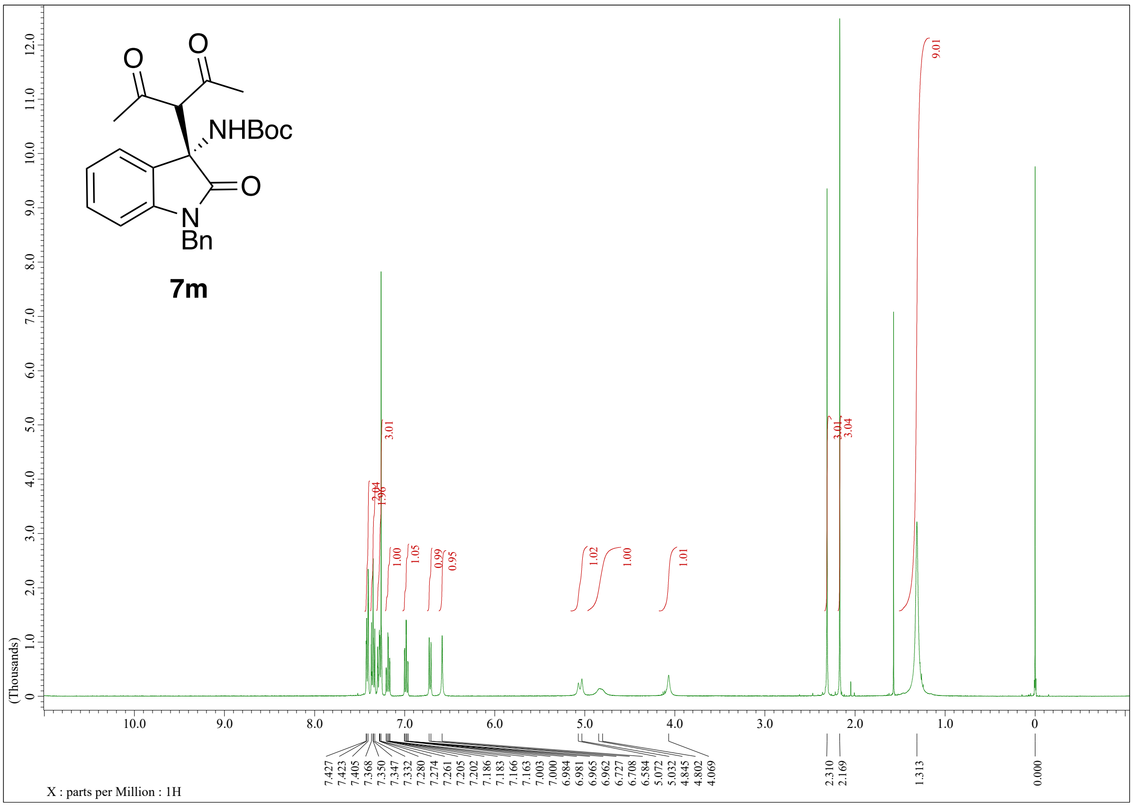

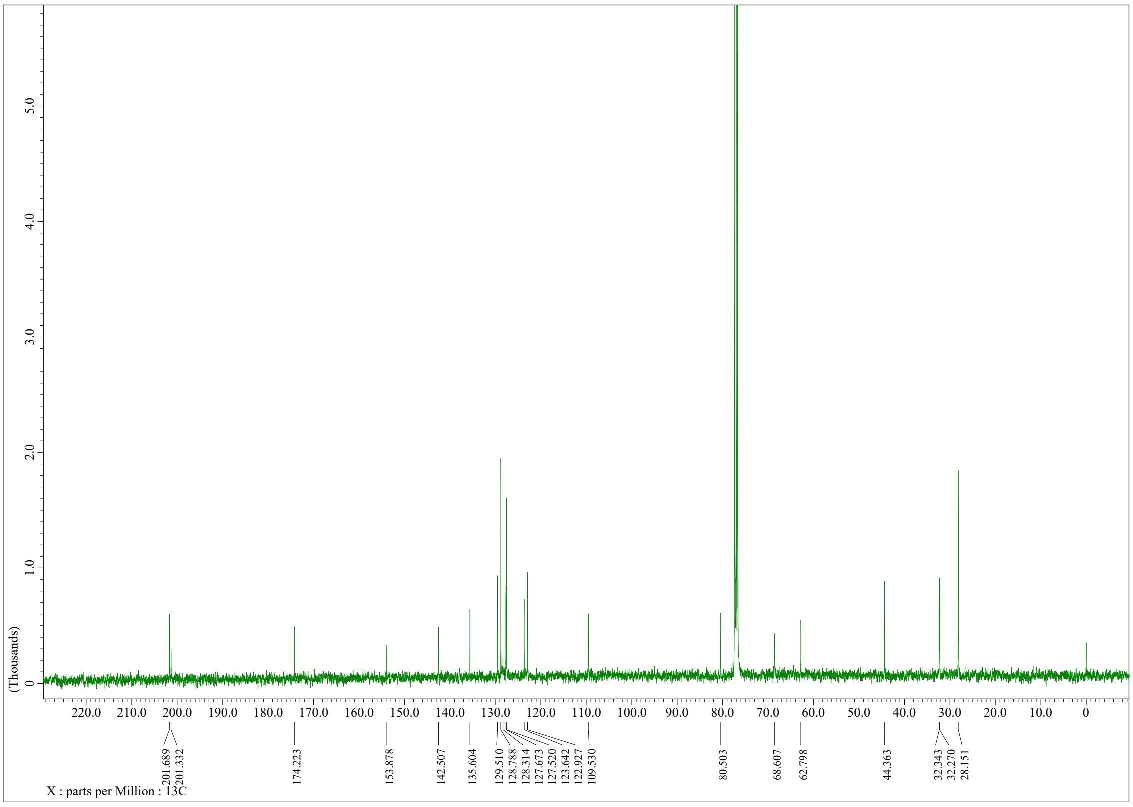

Racemic sample of **7m**; HPLC (CHIRALPAK IC column, hexane/2-propanol = 95/5, flow rate 1.0 mL/min, 25 °C, 210 nm)

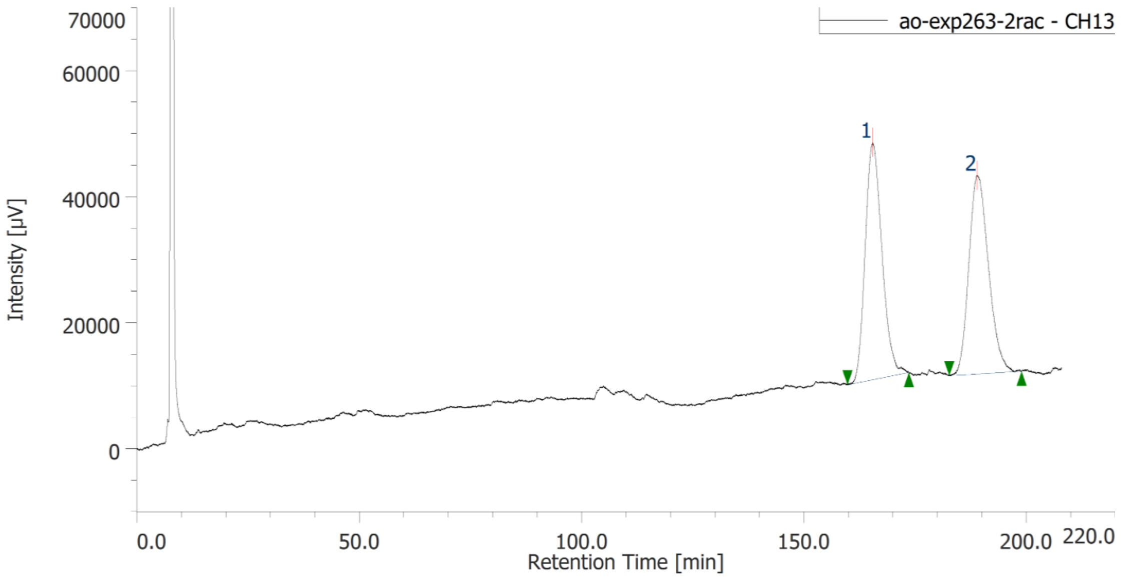

#	ピーク名	CH	tR [min]	面積 [µV·sec]	高さ [µV]	面積%	高さ%
1	Unknown	13	165.415	9866216	37638	50.5	54.4
2	Unknown	13	188.908	9658658	31514	49.5	45.6

7m catalyzed by **4a**

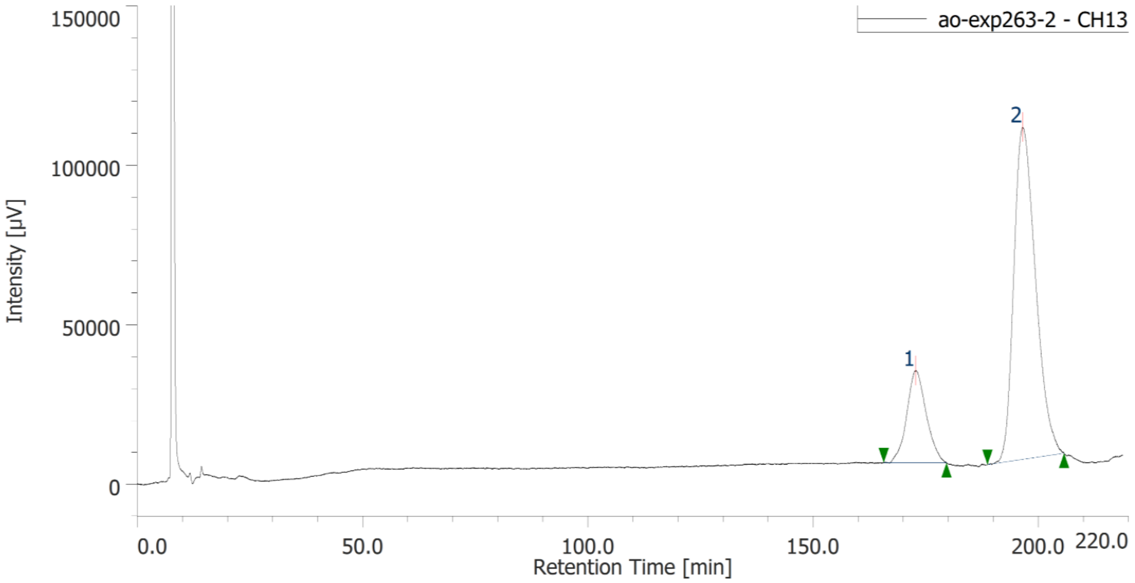

#	ピーク名	CH	tR [min]	面積 [µV·sec]	高さ [µV]	面積%	高さ%
1	Unknown	13	172.718	8618129	28868	19.8	21.7
2	Unknown	13	196.508	34916305	104057	80.2	78.3

Racemic sample of **7m**; HPLC (CHIRALPAK IA column, hexane/2-propanol = 80/20, flow rate 1.0 mL/min, 25 °C, 254 nm)

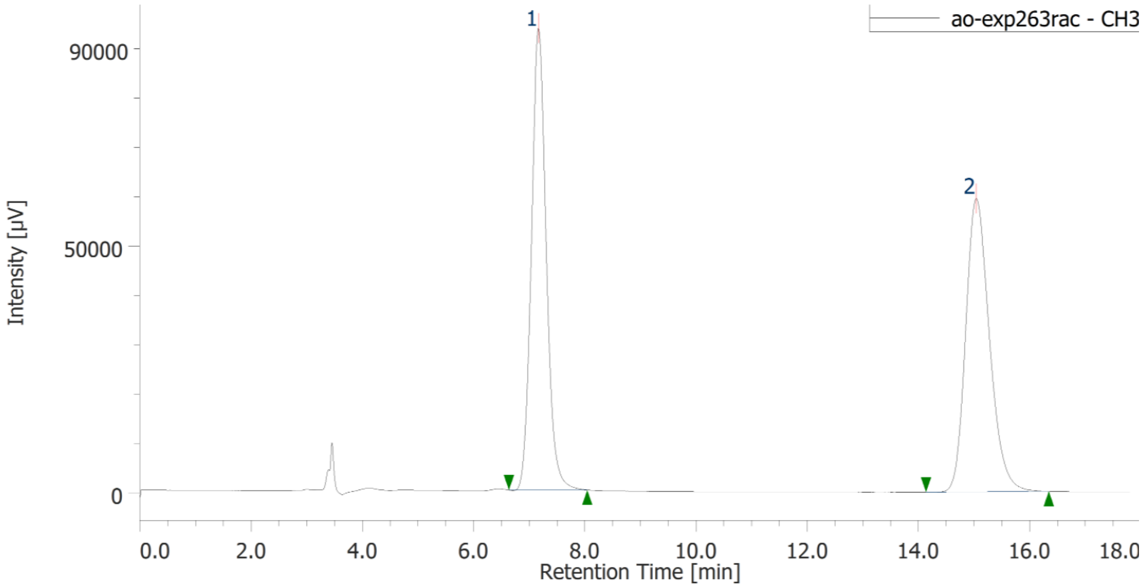

#	ピーク名	CH	tR [min]	面積 [µV·sec]	高さ [µV]	面積%	高さ%
1	Unknown	3	7.167	1734570	93567	49.6	61.2
2	Unknown	3	15.033	1760787	59373	50.4	38.8

7m catalyzed by **4a**

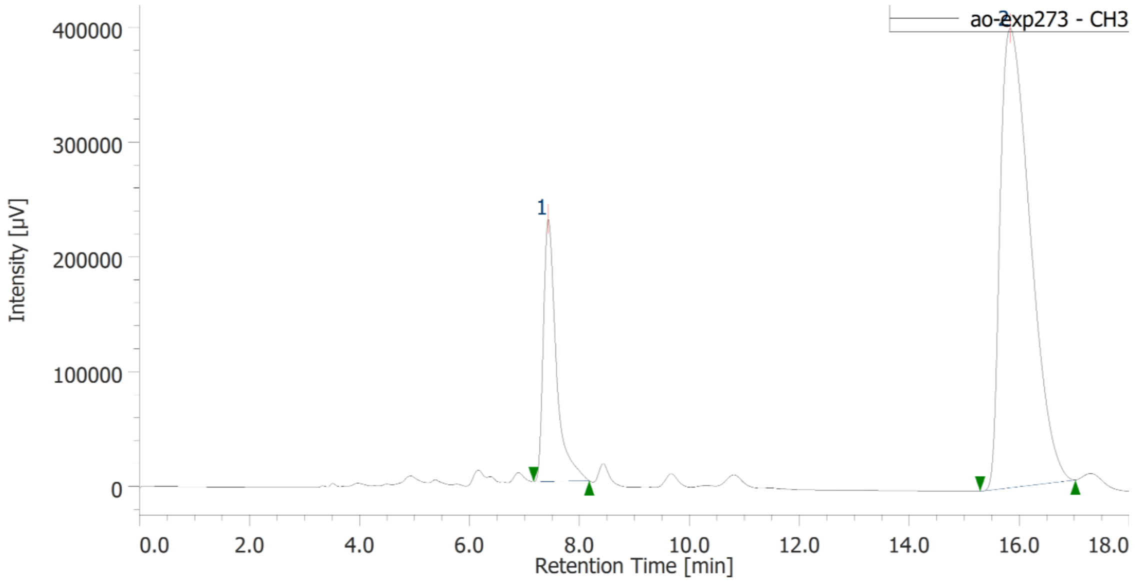

#	ピーク名	CH	tR [min]	面積 [µV·sec]	高さ [µV]	面積%	高さ%
1	Unknown	3	7.433	3741533	228637	19.7	36.4
2	Unknown	3	15.833	15226451	400186	80.3	63.6

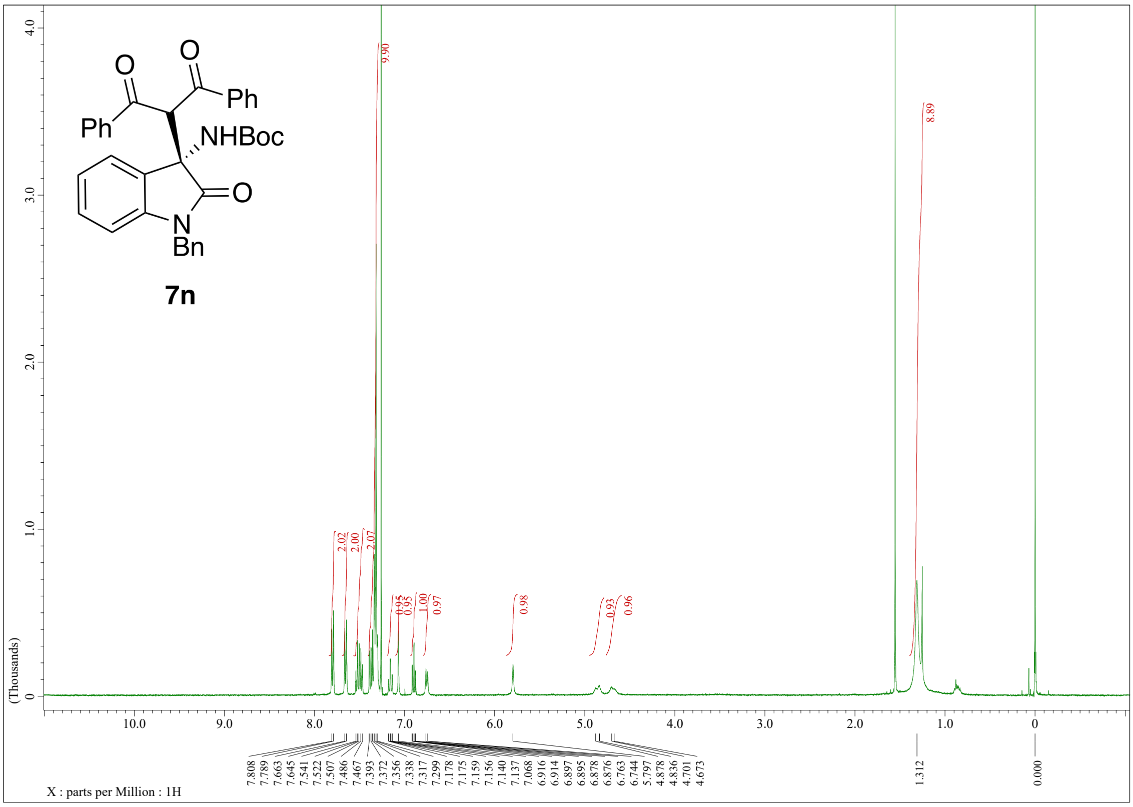

7n
NHBoc
Ph
O
N
Bn
(Thousands)
X : parts per Million : 1H
2.02 2.00 2.07 9.90 0.95 0.95 1.00 0.97 0.98 0.93 0.96 8.89
7.808 7.789 7.663 7.645 7.541 7.522 7.507 7.486 7.467 7.393 7.372 7.356 7.338 7.317 7.299 7.178 7.175 7.159 7.156 7.140 7.137 7.068 6.916 6.914 6.897 6.895 6.878 6.876 6.763 6.744 5.797 4.878 4.836 4.701 4.673
1.312
0.000

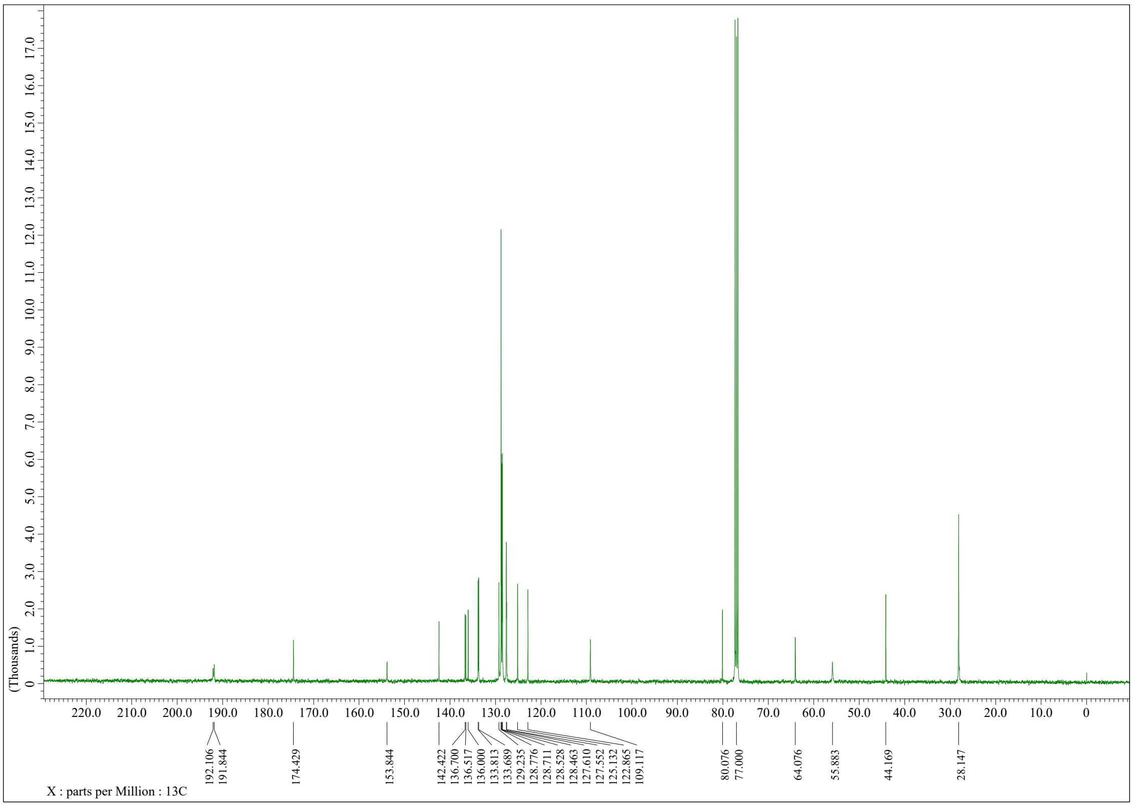

(Thousands)
X : parts per Million : 13C
192.106 191.844
174.429
153.844
142.422 136.700 136.517 136.000 133.813 133.689 129.235 128.776 128.711 128.528 128.463 127.610 127.552 125.132 122.865 109.117
80.076 77.000
64.076
55.883
44.169
28.147

Racemic sample of **7n**

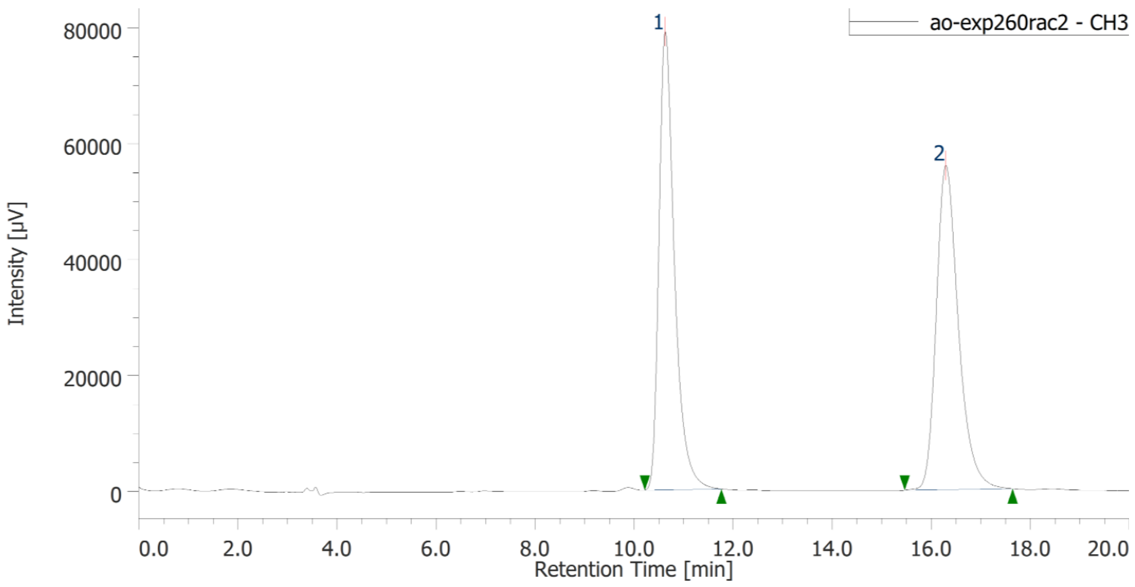

#	ピーク名	CH	tR [min]	面積 [μV·sec]	高さ [μV]	面積%	高さ%
1	Unknown	3	10.625	1718643	79049	49.7	58.6
2	Unknown	3	16.292	1737799	55939	50.3	41.4

Racemic sample of **7n**

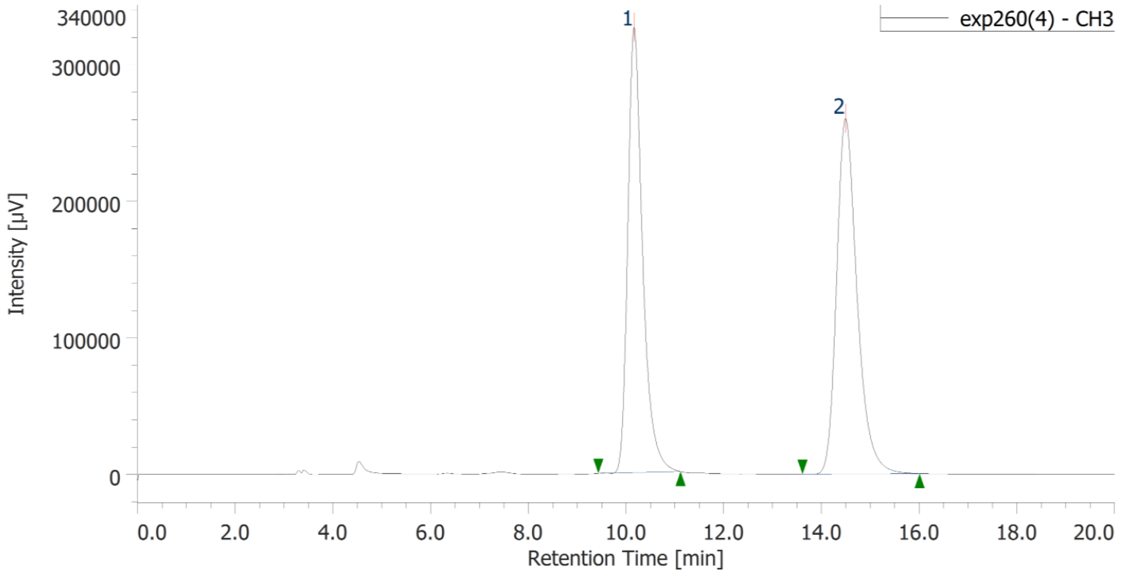

#	ピーク名	CH	tR [min]	Area [μV·sec]	Height [μV]	Area%	Height%
1	Unknown	3	10.17	6873655	325893	47.565	55.592
2	Unknown	3	14.49	7577525	260333	52.435	44.408

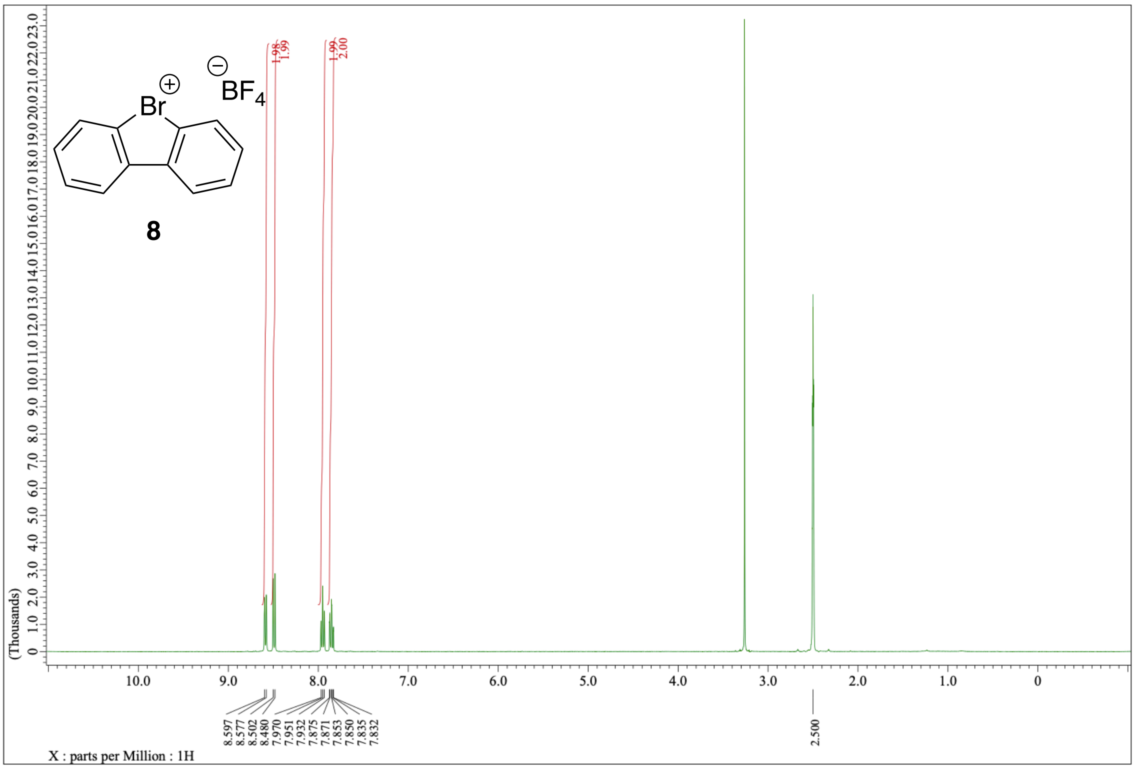

Br
BF4
8
(Thousands)
1.98
1.99
1.99
2.00
8.597
8.577
8.502
8.480
7.970
7.951
7.932
7.875
7.871
7.853
7.850
7.835
7.832
2.500
X : parts per Million : 1H

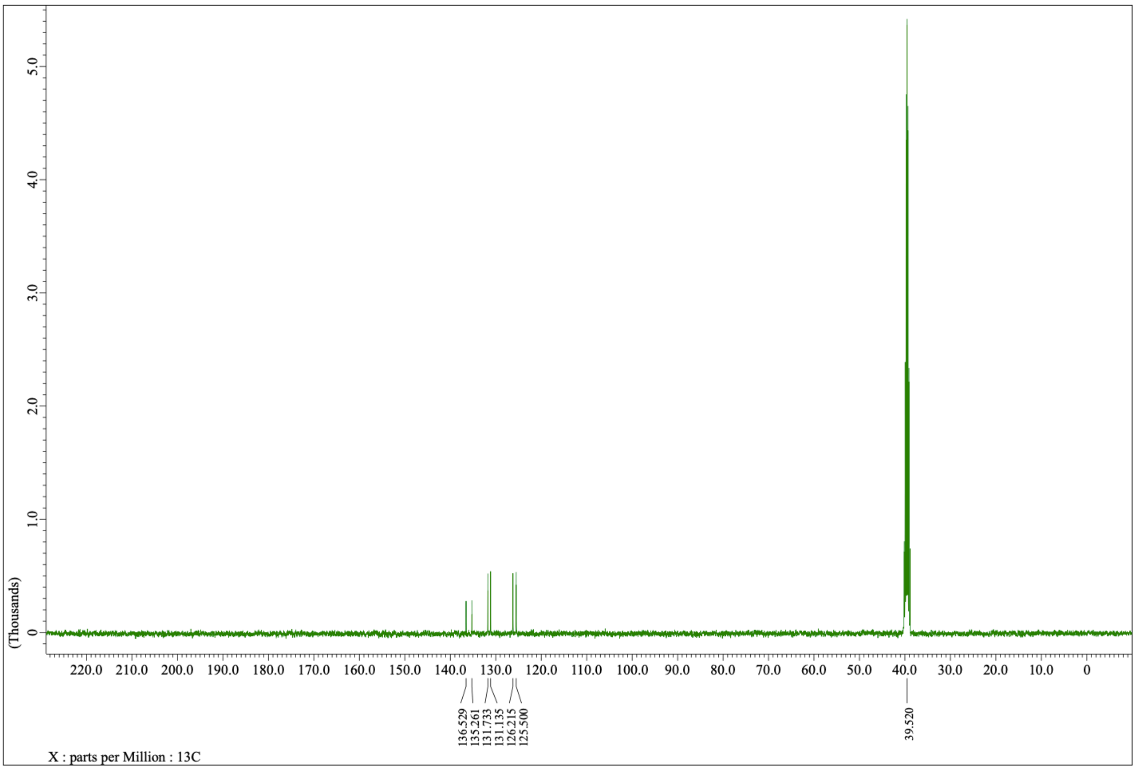

(Thousands)
136.529
135.261
131.733
131.135
126.215
125.500
39.520
X : parts per Million : 13C

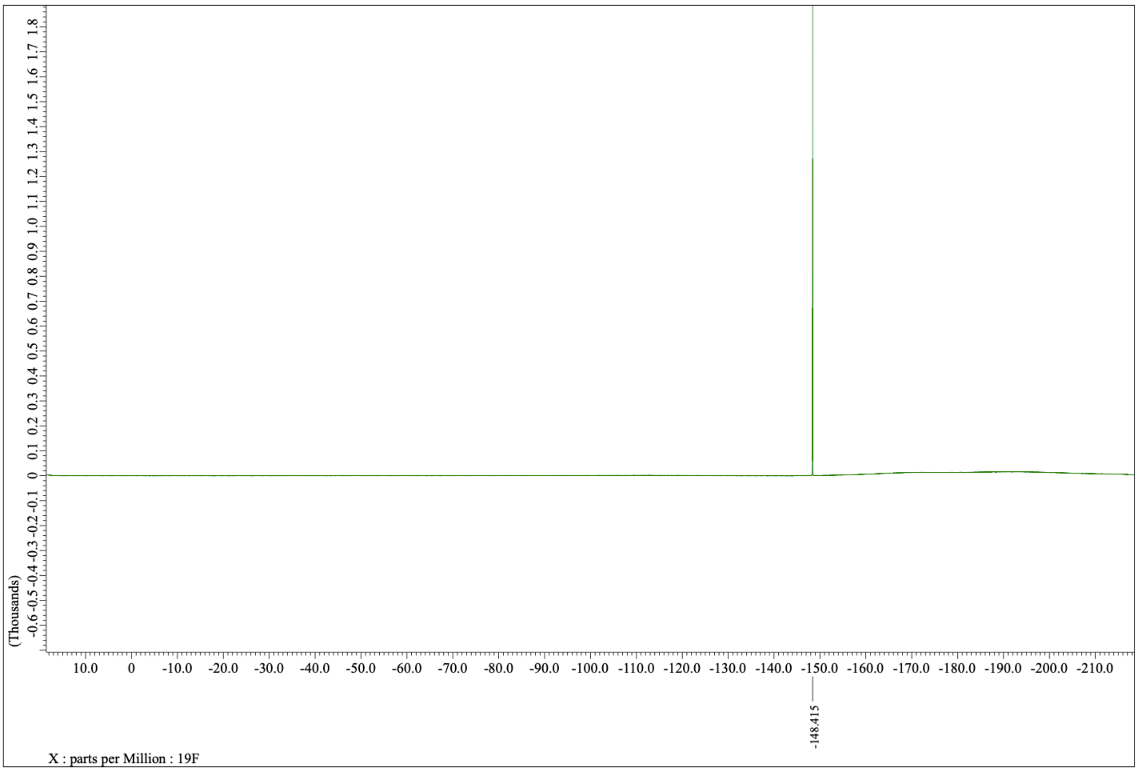
(Thousands)
-148.415
X : parts per Million : 19F

Optimized structure of **4a**.

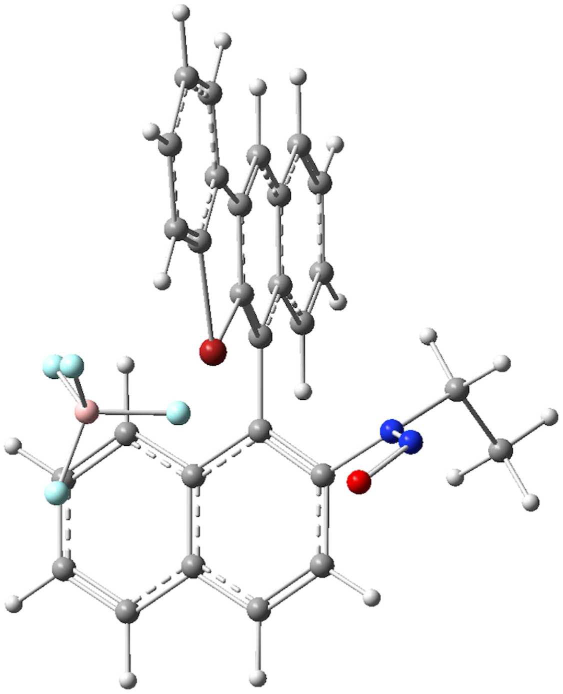

E(RM062X) = -4259.202216 Hartree

Center Number	Atomic Number	Atomic Type	Coordinates (Angstroms) X	Y	Z
1	6	0	4.130674	3.760648	-0.945786
2	6	0	4.578837	2.440055	-0.697811
3	6	0	3.681010	1.435511	-0.443777
4	6	0	2.285711	1.702871	-0.424601
5	6	0	1.831700	3.035125	-0.663726
6	6	0	2.792129	4.049155	-0.928284
7	6	0	1.330455	0.672963	-0.151911
8	6	0	0.016464	1.043108	-0.131149
9	6	0	-0.473942	2.348883	-0.364874
10	6	0	0.447005	3.333627	-0.631231
11	6	0	1.713930	-0.740613	0.113789
12	6	0	2.170565	-1.580068	-0.953689
13	6	0	2.473308	-2.941885	-0.675657
14	6	0	2.309018	-3.437455	0.645972
15	6	0	1.872696	-2.624835	1.652750

16	6	0	1.577339	-1.263997	1.383552
17	6	0	2.313062	-1.105735	-2.284521
18	6	0	2.745788	-1.943636	-3.281052
19	6	0	3.056890	-3.294384	-3.001848
20	6	0	2.920880	-3.780874	-1.727425
21	6	0	-2.616843	1.272940	-0.034828
22	6	0	-1.929775	2.457129	-0.301375
23	6	0	-3.985398	1.143517	0.065707
24	6	0	-4.714886	2.317080	-0.124930
25	6	0	-4.083169	3.532056	-0.393510
26	6	0	-2.699344	3.608692	-0.482368
27	7	0	1.179952	-0.422776	2.470928
28	1	0	4.853984	4.543762	-1.148171
29	1	0	5.642082	2.223518	-0.710941
30	1	0	4.023903	0.421915	-0.258881
31	1	0	2.440759	5.060232	-1.114027
32	1	0	0.124420	4.353506	-0.822119
33	1	0	2.533850	-4.481473	0.844071
34	1	0	1.734552	-2.998203	2.661369
35	1	0	2.067377	-0.073281	-2.511724
36	1	0	2.843557	-1.569312	-4.294909
37	1	0	3.394519	-3.945143	-3.801794
38	1	0	3.145022	-4.819882	-1.502886
39	1	0	-4.472230	0.194354	0.256648
40	1	0	-5.796703	2.267240	-0.067263
41	1	0	-4.678184	4.427455	-0.538651
42	1	0	-2.211378	4.555115	-0.693602
43	5	0	-3.973368	-2.000666	-0.592377
44	9	0	-3.284830	-1.278186	-1.583335
45	9	0	-5.293984	-1.568369	-0.522996
46	9	0	-3.855791	-3.351590	-0.770715
47	9	0	-3.350015	-1.621387	0.664929
48	35	0	-1.389483	-0.203977	0.198871
49	8	0	-0.567576	-1.632223	2.837419
50	7	0	0.115685	-0.718935	3.240072
51	6	0	2.078339	0.600145	3.002236

52	1	0	2.236296	1.368119	2.238213
53	1	0	1.532330	1.055819	3.831509
54	6	0	3.408239	0.019216	3.466227
55	1	0	3.249288	-0.729562	4.247509
56	1	0	3.940790	-0.454513	2.635855
57	1	0	4.043127	0.812980	3.868596
